# The effect of air pollution on the transcriptomics of the immune response to respiratory infection

**DOI:** 10.1038/s41598-021-98729-8

**Published:** 2021-09-30

**Authors:** Daniel P. Croft, David S. Burton, David J. Nagel, Soumyaroop Bhattacharya, Ann R. Falsey, Steve N. Georas, Philip K. Hopke, Carl J. Johnston, R. Matthew Kottmann, Augusto A. Litonjua, Thomas J. Mariani, David Q. Rich, Kelly Thevenet-Morrison, Sally W. Thurston, Mark J. Utell, Matthew N. McCall

**Affiliations:** 1 Department of Medicine, Pulmonary and Critical Care Medicine Division, University of Rochester Medical Center, 601 Elmwood Avenue Box 692, Rochester, NY 14642 USA; 2 Environmental Health Science Center, University of Rochester Medical Center, Rochester, NY USA; 3 Department of Biostatistics and Computational Biology, University of Rochester Medical Center, Rochester, NY USA; 4 Department of Pediatrics, University of Rochester Medical Center, Rochester, NY USA; 5 Department of Medicine, Infectious Diseases Division, University of Rochester Medical Center, Rochester, NY USA; 6 Department of Public Health Sciences, University of Rochester Medical Center, Rochester, NY USA; 7 Institute for a Sustainable Environment, and Center for Air Resources Engineering and Science, Clarkson University, Potsdam, NY USA; 8 Department of Biomedical Genetics, University of Rochester Medical Center, Rochester, NY USA

**Keywords:** Viral infection, Bacterial infection, Influenza virus, Atmospheric chemistry

## Abstract

Combustion related particulate matter air pollution (PM) is associated with an increased risk of respiratory infections in adults. The exact mechanism underlying this association has not been determined. We hypothesized that increased concentrations of combustion related PM would result in dysregulation of the innate immune system. This epidemiological study includes 111 adult patients hospitalized with respiratory infections who underwent transcriptional analysis of their peripheral blood. We examined the association between gene expression at the time of hospitalization and ambient measurements of particulate air pollutants in the 28 days prior to hospitalization. For each pollutant and time lag, gene-specific linear models adjusting for infection type were fit using LIMMA (Linear Models For Microarray Data), and pathway/gene set analyses were performed using the CAMERA (Correlation Adjusted Mean Rank) program. Comparing patients with viral and/or bacterial infection, the expression patterns associated with air pollution exposure differed. Adjusting for the type of infection, increased concentrations of Delta-C (a marker of biomass smoke) and other PM were associated with upregulation of iron homeostasis and protein folding. Increased concentrations of black carbon (BC) were associated with upregulation of viral related gene pathways and downregulation of pathways related to antigen presentation. The pollutant/pathway associations differed by lag time and by type of infection. This study suggests that the effect of air pollution on the pathogenesis of respiratory infection may be pollutant, timing, and infection specific.

## Introduction

Respiratory infections are a leading cause of adult morbidity and mortality in the United States^1^. Short-term increases in ambient fine particulate air pollution (≤ 2.5 µm in diameter; PM_2.5_) concentrations have been associated with increased emergency department (ED) visits for influenza and culture negative pneumonia in adults^2^. Studies of adults in the USA have also observed associations between acute increases in air pollution concentrations and an increased risk of healthcare encounters for respiratory viral infection (RVI) or other lower respiratory tract infections (LRTI), including respiratory bacterial infection (RBI)^3–6^. With over 90% of the world breathing unhealthy air^7^, and large health care burden of respiratory infections^8^, understanding the mechanisms underlying air pollution effects on the innate immune response to respiratory infections is crucial.

The association between the inflammation caused by air pollution and disruption of the lung’s innate immune system, including epithelial barrier disruption, macrophage function, and protein/cytokine response, is typically studied via in vitro human cell and in vivo rodent models^9–19^. Although the majority of studies suggest that different air pollution components might predispose patients to RVI or RBI by multiple mechanisms, laboratory-based studies are generally limited to inbred mouse strains and/or only a single pollutant or a single category of pollution (traffic pollution), and do not fully capitulate complex events occurring in naturally exposed humans. Studying natural exposures also helps characterize pollutant effects that controlled exposure studies may miss. Epidemiologic studies allow examination of health responses to single pollutants as they occur within a pollutant mixture.

By examining the association between multiple pollutants and the transcriptional profiles of the peripheral blood of patients with respiratory infection we can better understand what specific gene pathways may be driving the relationship between specific particulate pollution and different types of infection including RVI and RBI. Studying respiratory infection types in aggregate can give important insight into potential shared mechanisms between the effects of air pollution on respiratory infection in general while studying infections individually may elucidate infections specific mechanisms applicable to only RVI or RBI. Furthermore, studying the potential mechanisms of the effects of air pollution on respiratory infection may help design potential risk reduction strategies (e.g. anti-inflammatory treatment) for patients exposed to air pollution during periods of high rates of respiratory infection.

**Figure 1:**
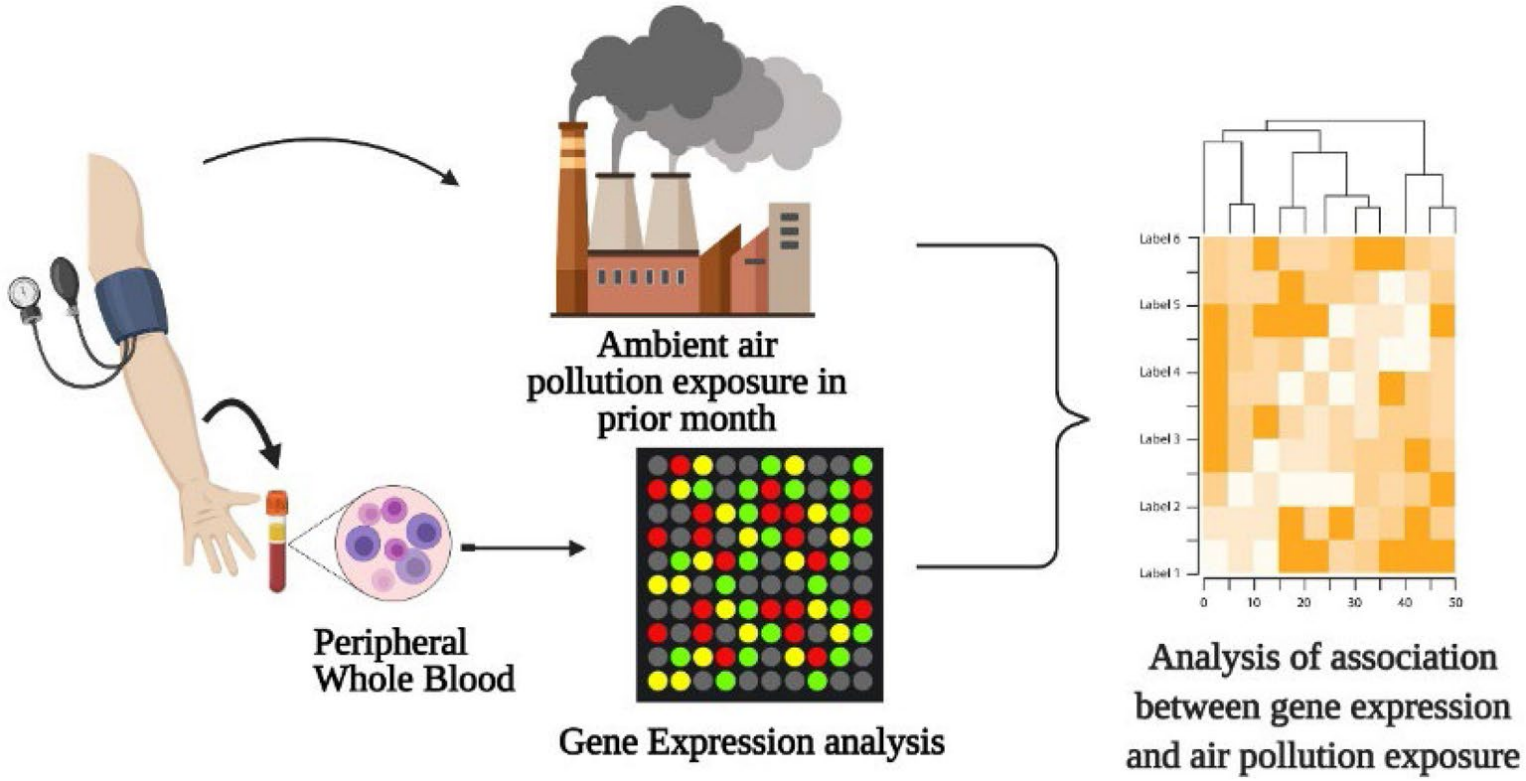
Study overview [Created with BioRender.com].

Although most literature focuses on PM_2.5_ and not its constituents, our prior source-specific study in NY State observed associations between combustion related constituents of PM_2.5_ and ED visits for influenza^20^. Among the PM_2.5_ constituent sources, we hypothesized that ambient combustion sources including traffic related air pollution (black carbon) and biomass burning (Delta-C) would be associated with dysregulation of key gene pathways within the immune response to respiratory infection. In this study, we focused on the gene expression patterns in patients with RVI as the primary outcome of interest associated with ambient air pollution. We also performed the same analysis in patients with RBI and combined viral and bacterial infection. First, we highlighted the patterns in gene expression across these three infection groups. Next, we analyzed the associations between air pollution concentrations and gene expression in all types of respiratory infection combined. We then analyzed the gene pathways associated with increases in air pollution concentrations in the aggregate group and visualized patterns of gene expression in key pathways for RVI, RBI and combined infection individually (Fig. 1). We report several associations between gene expression and air pollution exposure including several gene pathways relevant to respiratory infections, including immunity, protein folding and iron homeostasis.

## Results

### Patient characteristics

The majority of participants were white (76%), female (58%), and older, with median age range of 58–61 years old (Table 1). The majority of participants had viral infection (n = 66) and fewer patients had bacterial infection (n = 20) or coinfection (n = 25). The most common viruses included influenza (n = 22), respiratory syncytial virus (n = 19) and the most common bacterial infection was Streptococcus pneumonia (n = 12) (Supplemental Table S1 online). Over 90% of patients lived at home and the most common comorbidities included diabetes (34%), COPD (38%) and obesity (39%). Forty percent of participants were active smokers, with the lowest proportion of smokers in the viral infection group (35%) and the largest proportion of smokers in the co-infection group (52%). Inhaled steroid use was most prevalent within the bacterial infection group (65%) and least prevalent in the viral infection group (30%). Home oxygen use was most prevalent in the bacterial infection group (30%) and less common in the viral (15%) and combined infection groups 12%. Air pollution distributions for Rochester, NY during the same season as the hospitalizations are reported in Table 2. Although Rochester, NY is a medium-sized city, the average PM_2.5_ concentration is well below the EPA 24 hour fine particle standard of 35 μg/m^3^^21^. Moderate correlations (0.5 to 0.7) were observed between the pollutants (Supplemental Fig. S1 online).

**Table 1:** Patient characteristics.

Variable	Descriptions	Overall N = 111 (%)	Viral N = 66 (%)	Bacterial N = 20 (%)	Coinfection N = 25 (%)
Age		61.1 (26.1)*	59.7 (26.7)*	71.6 (25.9)*	59.6 (17.9)*
Gender	Female	64 (57.7)	41 (62.1)	10 (50)	13 (52)
	Male	47 (42.3)	25 (37.9)	10 (50)	12 (48)
Race	Black	25 (22.5)	11 (16.7)	7 (35)	7 (28)
	Other	2 (1.8)	1 (1.5)		1 (4)
	White	84 (75.7)	54 (81.8)	13 (65)	17 (68)
Ethnicity	Hispanic	17 (15.3)	10 (15.2)	2 (10)	5 (20)
	Non-Hispanic	94 (84.7)	56 (84.8)	18 (90)	20 (80)
Type of Residence	Home	103 (92.8)	61 (92.4)	19 (95)	23 (92)
Diabetes	Diabetes	38 (34.2)	24 (36.4)	6 (30)	8 (32)
CHF	Congestive heart failure	19 (17.1)	12(18.2)	3 (15)	4 (16)
COPD	COPD	42 (37.8)	22 (33.3)	9 (45)	11 (44)
CRF	Chronic renal failure	1 (0.9)			1 (4)
BMI		27.4 (13)*	27.2 (14.6)*	27.9 (11.1)*	27.4 (9.8)*
BMI category	Underweight	4 (4.6)	1 (2)	2 (11.8)	1 (4.8)
	Normal	31 (35.6)	18 (36.7)	5 (29.4)	8 (38.1)
	Overweight	18 (20.7)	11 (22.4)	3 (17.6)	4 (19)
	Obese	34 (39.1)	19 (38.8)	7 (41.2)	8 (38.1)
Smoking status	Never	30 (27)	19 (28.8)	6 (30)	5 (20)
	Active (within 3 months)	44 (39.6)	23 (34.8)	8 (40)	13 (52)
	Past	37 (33.3)	24 (36.4)	6 (30)	7 (28)
Oral Steroid	Yes	12 (10.9)	9 (13.8)	1 (5)	2 (8)
	No	98 (89.1)	56 (86.2)	19 (95)	23 (92)
Inhaled Steroid	Yes	47(42.3)	20 (30.3)	13 (65)	14 (56)
	No	64 (57.7)	46 (69.7)	7 (35)	11 (44)
Home O2	Yes	19 (17.1)	10 (15.2)	6 (30)	3 (12)
Flu vaccine	Yes	66 (62.3)	39 (61.9)	16 (84.2)	11 (45.8)
Pneumonia vaccine	Yes	56 (58.9)	32 (60.4)	13 (72.2)	11 (45.8)

*Age and BMI are Median (interquartile range).

**Table 2:** Ambient air pollution concentrations for all participants, at multiple lag times, stratified by infection type.

Pollutant	Lag period prior to diagnosis	Overall mean (Std)	Viral mean (Std)	Bacterial mean (Std)	Coinfection mean (Std)
AMP	0 to 6 days	873.22 (308.70)	902.39 (274.54)	827.48 (483.06)	832.53 (134.39)
	7 to 13 days	1008.29 (323)	984.63 (307.87)	1081.82 (381.58)	1019.45 (327.68)
	14 to 20 days	1142.28 (412.83)	1156.35 (457.73)	1156.7 (422.28)	1095.93 (276.88)
	21 to 27 days	1087.91 (451.2)	1116.54 (472.79)	1013.61 (483.29)	1064.06 (378.11)
Black carbon	0 to 6 days	0.52 (0.21)	0.52 (0.18)	0.52 (0.31)	0.51 (0.16)
	7 to 13 days	0.53 (0.16)	0.49 (0.13)	0.6 (0.19)	0.57 (0.18)
	14 to 20 days	0.56 (0.18)	0.56 (0.17)	0.54 (0.24)	0.57 (0.15)
	21 to 27 days	0.55( 0.21)	0.54 (0.2)	0.52 (0.21)	0.62 (0.22)
Delta-C	0 to 6 days	0.19 (0.11)	0.19 (0.11)	0.18 (0.11)	0.21 (0.09)
	7 to 13 days	0.21 (0.09)	0.18 (0.08)	0.24 (0.1)	0.26 (0.1)
	14 to 20 days	0.22 (0.10)	0.22 (0.09)	0.23 (0.13)	0.23 (0.09)
	21 to 27 days	0.24 (0.13)	0.23 (0.14)	0.25 (0.12)	0.28 (0.13)
PM_2.5_	0 to 6 days	7.79 (2.75)	7.66 (2.63)	8.01(3.58)	7.90 (2.34)
	7 to 13 days	8.33 (2.67)	7.72 (2.51)	9.58 (2.54)	8.80 (2.79)
	14 to 20 day	8.45 (3.05)	8.16 (3.4)	8.90 (3.12)	8.79 (1.84)
	21 to 27 days	8.15 (3.50)	7.86 (3.66)	8.62 (3.6)	8.55 (2.99)
Ultrafine particles	0 to 6 days	4611.46 (1120.64)	4594.73 (1026.12)	4890.24 (1512.57)	4365.42 (909.43)
	7 to 13 days	4835.69 (1221.7)	4815.96 (1163.35)	5186.72 (1646.82)	4619.66 (1000.51)
	14 to 20 days	5262.77 (1410.88)	5208.64 (1473.39)	5703.08 (1534.16)	5110.57 (1154.33)
	21 to 27 days	5161.78 (1595.44)	5276.92 (1604.38)	5309.07 (1799.33)	4753.53 (1425.86)

### Distinct patterns of gene expression within the highest variance genes correspond to infection type and ambient air pollution

**Figure 2:**
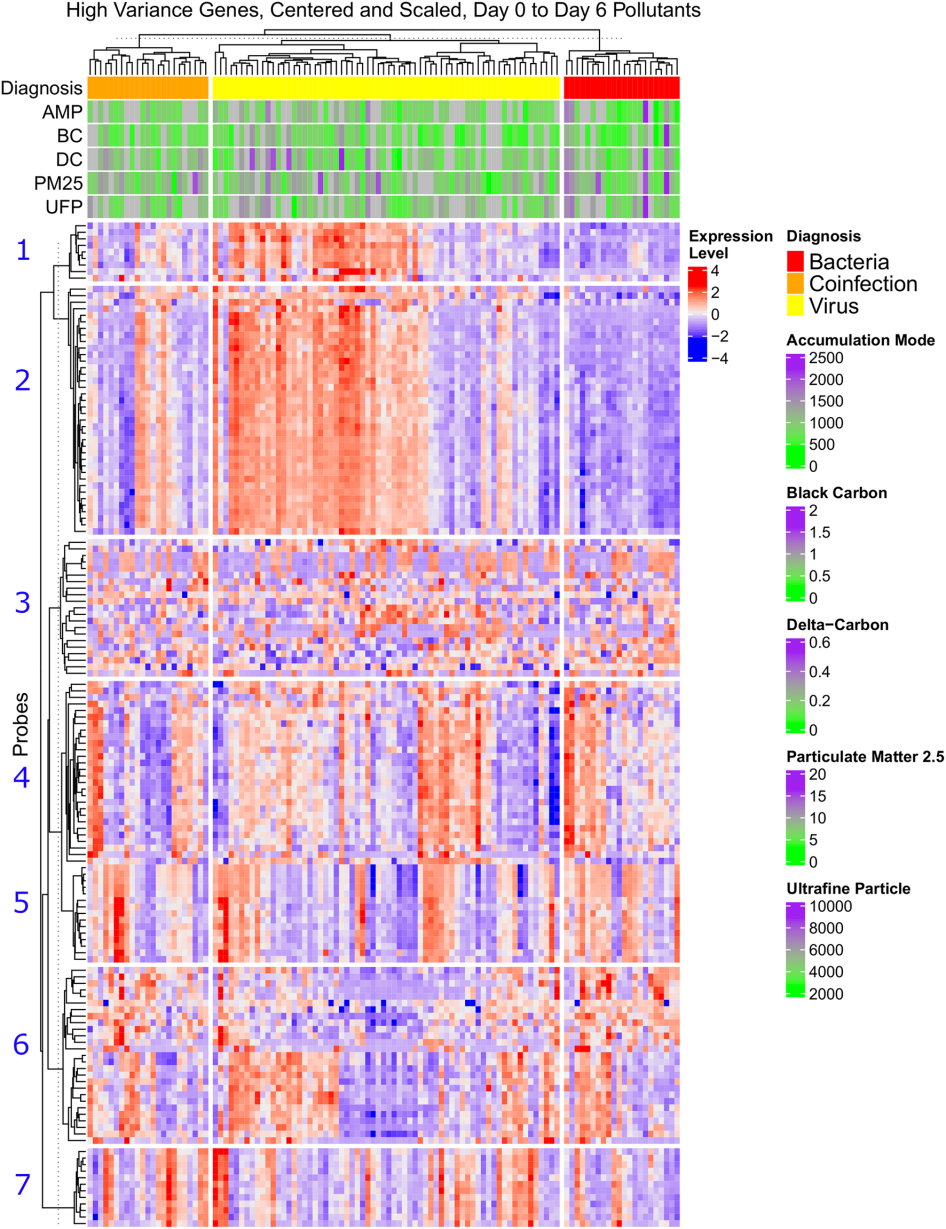
Heat map of the 150 highest variance genes and corresponding air pollution concentrations, stratified by type of respiratory infection and divided by participant (Columns) and gene cluster (Rows 1–7). The selected expression values were centered and scaled to have mean zero and variance one. The dendrograms were produced using hierarchical clustering on either genes or samples using Euclidean distance and complete linkage. A qualitative threshold (dotted line) was selected to define gene clusters based on the hierarchical clustering.

We performed an initial exploratory analysis focusing on the 150 genes with the highest variance across samples. In this analysis, we observed infection specific differences in gene expression (Fig. 2). Based on the hierarchical clustering of genes, we defined 7 distinct gene clusters. One cluster (#2) showed clear expression differences between infections, as previously shown by Suarez et al.^22^. Though air pollution modeling was not performed in this exploratory portion of the analysis, increased expression of genes in this cluster visually corresponded to high pollution levels among the viral infection samples. In contrast, elevated expression of these genes in patients with bacterial or combined infection did not clearly correspond to high pollution levels.

Within a specific type of respiratory infection, the expression levels were not consistent across all high variance genes. We observed a higher gene expression corresponding to the highest air pollution concentrations in Cluster #2 for viral infection compared with Clusters #3–6. While other gene clusters captured consistent expression patterns across subjects, these differences were not clearly associated with either infection or pollutant levels.

In the heatmap of the 150 most variable genes (Fig. 2), the 38 individual genes within Cluster #2, had the highest gene expression within the viral infection group and included a variety of immune related genes (Supplemental Table S2 online). For example, there were multiple interferon induced protein coding genes including IFI44L, OAS1 and MX1. IFI27, IFIT^1–3^ and RSAD2 participate in Interferon gamma signaling and the innate immune response. OAS2 also participates in the innate immune response by encoding for the 2-5A synthetase family. The HERC5 gene is upregulated by endothelial cell inflammation. This result suggests exposure to particulate air pollutants in the week prior to hospitalization with RVI may be associated with a more exuberant immune and inflammatory response.

### Transcriptomic analysis quantifies the associations between the expression of individual genes and both infection type and ambient air pollution

To quantify the association between expression of individual genes and air pollution measurements prior to hospitalization for individuals with a respiratory infection, we performed a LIMMA analysis controlling for sex and infection type (see “Methods” for details). This analysis was performed on all measured genes.

#### Few individual genes associated with changes in air pollution

We then analyzed the association between air pollution and gene expression in the overall study population including all infection types. The average effect of each 1 µg/m^3^ increase in DC in the prior 7 days, regardless of infection type, is a log2-fold increase of 3.5 for RNF14 and 2.4 for UBE2F, two genes participating in antigen presenting cell pathways (Supplemental Table S3 online). Other notable genes upregulated in association with increased concentrations of DC at the 7-day lag period include a 3.1 log2-foldchange increase in MAP2K3 (TLR signaling) and 2.9 log2-fold change increase in ADORA1 (pro-inflammatory monocyte activation). Two genes involved in hemoglobin synthesis (HMBS) and blood cell size (TMCC2) were also upregulated in association with increased DC concentrations in the seven days prior to hospitalization. No significant changes in genes were associated with increased concentrations of PM_2.5_, BC, UFP or AMP. To further investigate these putative associations between gene expression and air pollution, as well as others not readily apparent from the unsupervised and individual gene analysis, we completed a pathway analysis using the differentially expressed genes group by clusters.

#### Differential expression of viral versus bacterial infection to independently replicate baseline expression differences between RVI and RBI (not factoring in air pollutants)

The top genes in the individual LIMMA analyses that were differentially expressed in peripheral blood samples of patients hospitalized with viral infection vs. bacterial (baseline in the model) infection were ISG15, TIMM10, IFI27, IFI44L and OAS2 (Supplemental Table S4 online). These genes were consistently differentially expressed regardless of which pollutant was included in the model. For consistency, we specifically presented the gene expression values within the same group of participants with data for the pollutant DC at the 0–6 lag period (as in the above pollution focused individual gene analysis), There was a log2-fold increase of 3.3 for ISG15 and 2.2 for TIMM10, two genes participating in antigen presenting cell pathways. IFI 27, IFI44 (and its paralogIFI44L), are part of the interferon induced response to RVI and were observed to have a log2-fold increase in the 3-4 range. Finally, OAS-2, a part of the innate immune system, was observed to have a 2.5 log2-fold increase. Three of the genes (IFI44, OAS2 and IFI27) that we observed in this replication effort (Table S4) matched the classifier genes found in the original Suarez et al.^22^ study. Though we control for infection type in our model considering air pollution, highlighting the genes that are classifiers distinguishing RBI from RVI (independent of pollution) is helpful to understand whether or not there is overlap between the genes that are associated with air pollution in patients with respiratory infection and the genes that are associated with respiratory infection alone (without accounting for pollution).

#### Pathway analysis to characterize the broad areas of gene expression associated with different air pollutants in patients with all types of respiratory infection together and targeted infection specific heatmaps

With a signal appearing between high concentrations of air pollution and gene expression within Fig. 2, we then identified specific gene pathways that were associated with modelled increases in air pollution concentrations across all infections (Table 3 and Supplemental Tables S5–S9). When analyzing the association between air pollutants and gene pathways using the CAMERA method, we observed several broad pathways with implications on innate immunity. Then, given the differences in gene expression across infections, we constructed heatmaps of specific pathways of interest for infection specific changes at multiple pollution lags (Supplemental Fig. S2–S12 Online).

**Table 3:** Pattern of association between gene pathways and increased concentrations of multiple particulate matter pollutants at multiple lag times (bold-upregulated, italics-downregulated).

Gene pathway name	BC (lag)	DC (lag)	AMP (lag)	PM_2.5_ (lag)	UFP (lag)
Establishment_of_protein_localization_to_endoplasmic_reticulum	**21–27**	**7–13**	**0–6**	*7–13*	**14–20**
Ribosomal_subunit	**21–27**	**7–13**	**0–6**	*7–13*	**14–20**
Structural_constituent_of_ribosome	**21–27**	**7–13**	**0–6**	*7–13*	**14–20**
Specific_granule_lumen		*21–27*	*0–6*	*0–6*	*0–6*
Cotranslational_protein_targeting_to_membrane	**21–27**	**7–13**	**0–6**		**14–20**
Cytosolic_large_ribosomal_subunit	**21–27**	**7–13**	**0–6**		**7–13**
Cytosolic_ribosome	**21–27**	**7–13**	**0–6**		**14–20**
Ferric_iron_binding		**0–6**	**21–27**	**21–27**	**0–6**
Ferrous_iron_binding		**0–6**	**21–27**	**21–27**	**0–6**
Large_ribosomal_subunit	**21–27**	**7–13**	**0–6**		**14–20**
Nuclear_transcribed_mrna_catabolic_process_nonsense_mediated_decay	**21–27**	**7–13**	**0–6**		**14–20**
Protein_localization_to_endoplasmic_reticulum	**21–27**	**7–13**	**0–6**		**14–20**
Protoporphyrinogen_Ix_biosynthetic_process		**0–6**	**21–27**	**0–6**	**0–6**
Protoporphyrinogen_Ix_metabolic_process		**0–6**	**21–27**	**0–6**	**0–6**
Ribosome	**21–27**	**7–13**	**0–6**		**14–20**
Translational_initiation	**21–27**	**7–13**	**0–6**		**14–20**
Catalytic_step_2_spliceosome		*0–6*	*21–27*	*14–20*	
Mitochondrial_translation		*0–6*	*21–27*	*7–13*	
U2_type_catalytic_step_2_spliceosome		*0–6*	*21–27*	*7–13*	
U2_type_spliceosomal_complex		*0–6*	*21–27*	*7–13*	
Nuclear_transcribed_mrna_catabolic_process	**21–27**		**0–6**		**14–20**
Translation_preinitiation_complex		**14–20**	**0–6**		**7–13**
Specific_granule			*0–6*	*0–6*	*0–6*
Small_ribosomal_subunit	**21–27**			*7–13*	**14–20**
Cytoplasmic_translation	**21–27**	**14–20**			**14–20**
Cytosolic_small_ribosomal_subunit	**21–27**	**7–13**			**14–20**
Protein_targeting_to_membrane	**21–27**	**7–13**			**14–20**
Viral_gene_expression	**21–27**	**7–13**			**14–20**
Cytoplasmic_side_of_rough_endoplasmic_reticulum_membrane			*7–13*	*7–13*	
General_transcription_initiation_factor_activity			*21–27*	*21–27*	
Mitochondrial_gene_expression			*21–27*	*7–13*	
Myeloid_leukocyte_mediated_immunity			*0–6*		*7–13*
Positive_regulation_of_double_strand_break_repair_via_homologous_recombination			*21–27*	*21–27*	
Secretory_granule_membrane			*0–6*		*7–13*
Specific_granule_membrane			*0–6*		*0–6*
Superoxide_anion_generation			*0–6*		*7–13*
Tertiary_granule			*0–6*		*7–13*
Autolysosome			**21–27**	**21–27**	
Oxidoreductase_activity_oxidizing_metal_ions			**21–27**	**21–27**	
Oxidoreductase_activity_oxidizing_metal_ions_oxygen_as_acceptor			**21–27**	**21–27**	
Regulation_of_ryanodine_sensitive_calcium_release_channel_activity	*21–27*	*21–27*			
T_cell_receptor_binding	*14–20*			*14–20*	
Mda_5_signaling_pathway	**0–6**			**0–6**	
Polysomal_ribosome	**21–27**				**14–20**
Polysome	**21–27**				**21–27**
Cajal_body		**0–6**		*14–20*	
Lewy_body		**0–6**			**0–6**
Porphyrin_containing_compound_metabolic_process		**0–6**			**0–6**
Regulation_of_mitochondrial_electron_transport_nadh_to_ubiquinone		**0–6**			**0–6**
Spectrin_associated_cytoskeleton		**0–6**			**0–6**
Hemoglobin_metabolic_process				**7–13**	**0–6**
Purine_deoxyribonucleotide_binding				**0–6**	**0–6**

#### Delta-C (DC)

Multiple different gene pathways associated with red blood cell synthesis and iron handling were significantly upregulated in association with increases in DC concentrations at all lag periods (Table 3 and Supplemental Table S5 online). Gene pathways associated with endoplasmic reticulum activity (protein folding essential for cytokine production in the immune system) were also upregulated by increased DC concentrations at the 7–13, 14–20 and, 21–28 lag periods while increased concentrations of DC at the 7–13 and 14–20 lag periods were associated with upregulation of a viral replication pathway.

In the infection specific heatmaps, DC was observed to have different patterns of expression in the iron homeostasis pathway (Protoporphyrinogen IX Biosynthetic Process) when comparing infections at the 0–6 and 21–27 lag times (Supplemental Figs. S2–S7 online). While the highest gene expression in iron homeostasis was observed in patients with bacterial or viral infection at the highest concentrations of DC, the combined infection group displayed a mixed to decreased expression. There was no clear pattern of gene expression along the pollution concentration gradient when comparing infection type within an endoplasmic reticulum or viral gene expression pathway.

#### Black carbon (BC)

**Figure 3:**
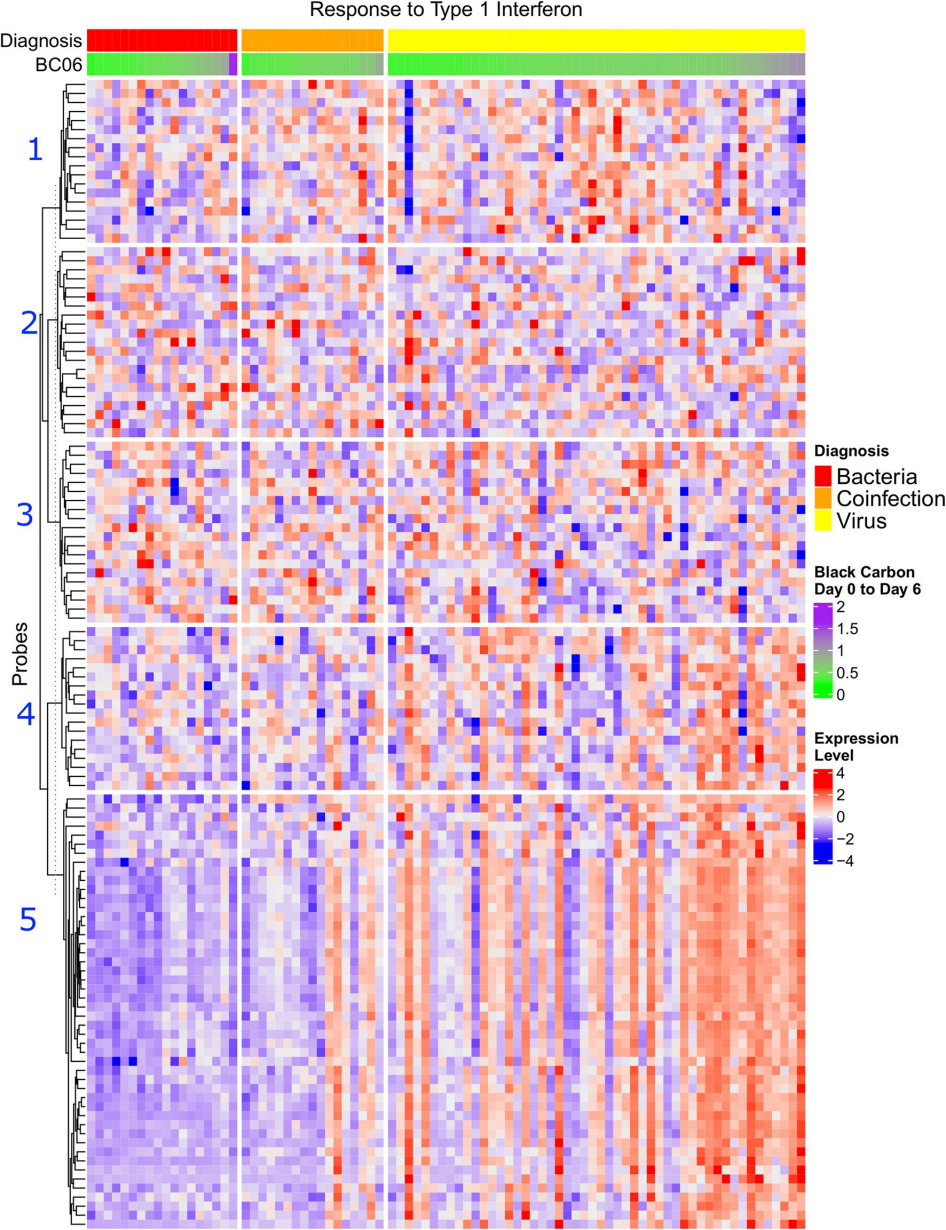
Infection specific heatmap of Type 1 Interferon pathway with corresponding black carbon concentrations divided by participant (Columns) and Cluster (Rows 1–5). Data were processed and dendrograms generated as in Fig. 2.

Increased concentrations of BC were associated with upregulation of a combination of anti-viral pathways and pro-viral pathways in the 0–13 day lag periods (Table 3 and Supplemental Table S6 online). In the 14–20 day lag period, several immune pathways were significantly down regulated in association with a one-unit increase in BC concentrations. Differences in gene expression of the type 1 interferon pathway were observed between infection types for BC (Fig. 3). For BC at the 0–6 lag period, the highest gene expression was found in Cluster #5 of genes for patients with RVI and the lowest expression was observed in this cluster for patients with respiratory bacterial infection (RBI). Patients with RVI appeared to be driving the positive association between BC and the type 1 interferon pathway.

#### PM_2.5_

In the 7–20 day lag periods, multiple ribosomal/ER related pathways were downregulated. Iron binding pathways were upregulated in the 20–27 day lag period and iron homeostasis pathways were also upregulated at all lag times (Table 3 and Supplemental Table S7 online).

For PM_2.5_ at the 0–6 and 14–20 day lag periods, the highest expression of iron homeostasis genes was observed within patients with bacterial infection (Supplemental Figs. S8–S9 online). At the 14–20 lag days for PM_2.5_, patients with RVI had the lowest gene expression within the iron homeostasis pathways. In patients with combined infection, low levels of gene expression in iron homeostasis was also observed at the 0–6 lag time for the highest concentrations of PM_2.5_. These findings indicate that the patients with RBI were driving the signal in the combined analysis of all infections.

#### Ultrafine particle (UFP)

A one-unit increase in UFP was associated with down regulation of several immune related pathways at the 7–13 day lag period, including neutrophil and myeloid activation and regulation of leukocyte degranulation (Table 3 and Supplemental Table S8 online). In the 0–13 day lag period, increased concentrations of UFP were associated with upregulation of both iron binding and heme related iron pathways. Finally, we observed upregulation of ER related pathways in the 14–27 lag time associated with a one unit increase in UFP concentrations.

In the infection specific heatmaps for UFP at the 14–20 and 21–27 lag time, we observed the highest expression of viral gene expression pathway genes in patients with viral infection, but there was no clear pattern within bacterial or combined infection (Supplemental Figs. S10–S11 online). While the infection specific heatmaps showed similar expression pattern for iron homeostasis across different infections, there were distinct expression patterns for immune specific (highest expression for viral infection) and protein folding pathways (highest for combined infection) when comparing infections.

#### Accumulation mode particles (AMP)

One unit increases in AMP were associated with upregulation of multiple ER associated pathways in the 0–6 lag period and upregulation of iron homeostasis related pathways at the 21–27 day lag period. An increased concentration of AMP was associated with downregulation of a myeloid leukocyte pathway at the 0–6 lag time (Table 3 and Supplemental Table S9 online).

In the infection specific heatmaps for AMP at the 0–6 lag period, we observed the highest level of expression in the mRNA catabolic pathway in patients with bacterial infection and lowest levels in patients with viral infection (Supplemental Fig. S12 online).

### Summary of the comparison between gene pathway expression patterns across pollutants

The pathways associated with all pollutants included genes related to the upregulation of endoplasmic reticulum related pathways (except for downregulation seen with PM_2.5_), including ribosomal synthesis (Table 3). Iron binding and hemoglobin related pathways were also commonly affected by all pollutants except for BC. Out of all the pollutants, BC and UFP were associated with the greatest number of immune and viral related gene pathways.

When broadly visualizing the patterns of relative gene expression divided by type of pollution, the most distinct differences were observed when comparing RVI and RBI. Combined infection commonly displayed a mixed (indeterminate) pattern. The strongest gene expression differences when comparing between infections occurred with BC at the 0–6 lag period for Type 1 interferon (Fig. 3). These differences associated with BC displayed decreased expression in the patients with bacterial infection and higher expression in patients with viral infection. One of the most consistent patterns of discordance across multiple pollutants was observed within an iron homeostasis pathway. The highest concentrations of pollutants including AMP, DC and UFP corresponded to higher expression of the middle to lower clusters of genes at the 0–6 and 21–27 lag periods for both viral and bacterial infection, but the relationship was inconsistent in the combined infection group. In the infection specific analysis, there were also multiple examples of low gene expression, which appeared to be independent of air pollution levels.

### Targeted exploration of individual genes within the Interferon pathway.

Given the contrast between the gene expression patterns corresponding to the highest pollutant concentrations among the types of infection within Cluster #5 of the Response to Type 1 Interferon pathway (Fig. 3), we examined the 18 individual genes presents within this cluster (Table 4). The majority of the genes in this cluster were related to the immune response to infection including OAS-1/3, IFI44L, HLA-A and HLA-DRB-1/4. Genes related to ribosomal activity (RPS4Y-1) and heme biosynthesis (ALAS2) were also present in this cluster. This review of individual genes highlights the overlap between infection related pathways (Interferon) and the ribosomal and heme related genes. OAS2 and IFI44L were also found to be expressed in a high level in patients with RVI in the analysis of 150 highest variance genes (Fig. 2). The gene expression in the other clusters in the heatmap did not appear to correspond to pollution levels when comparing across infection types. The genes in Cluster #2, for example, contains a variety of genes related to acute phase reaction (ORM1) viral replication (IFIT1) and granulocyte regulation (CD17, CLC). However, Cluster #2 also has genes that are not directly related to immunity including genes related to collagen formation (COL9A2) and the urea cycle (ARG1). In summary, the cluster which displays the greatest infection specific differences in gene expression corresponding to high pollutant concentrations (Cluster #5) has a higher proportion of immune related genes than the more diversely populated Cluster #2.

**Table 4:** Genes present within clusters 2 and 5 of the type 1 interferon/ black carbon 0–6 day lag heatmap.

Cluster 2	Cluster 5
ALAS2	ALAS2
ARG1	BPI
CD177	HLA-A
CLC	HLA-DRB1 and HLA-DRB4
COL9A2	IFI44
GBP1	IFI44L
HP	IL1R2
IFI6	MAP2K3
IFIT1	NA
IGLL1	OAS2 and OAS3
LOC644936	RETN
LY6E	RPS4Y1
NA	SPATS2L
ORM1	TUBB2A
XAF1	VAV3

Descriptions of additional sensitivity analysis on participants who are smokers (Supplemental Table S10 online) and highlighting individual genes present in the aforementioned significant gene pathways (Supplemental Tables S11–S15 online) are provided in the supplemental information.

## Discussion

In patients hospitalized with viral, bacterial and combined infection, we observed associations between gene expression and air pollution exposure in the 1 to 4 weeks prior to hospitalization, including gene pathways related to immunity, protein folding and iron homeostasis.

As hypothesized, combustion related air pollutants including DC (marker of wood burning) and BC (marker of traffic pollution) were associated with changes in gene expression of multiple pathways related to the immune response to respiratory infection. All pollutants were associated with genes involved in protein folding, and all but BC were associated with key iron homeostasis pathways. Of all pollutants, BC was associated with upregulation of the largest number of immune specific pathways in the week prior to infection. In the infection specific exploration of selected pathways, we observed similar patterns of gene expression with the overall pathway analysis at the early lag times (0–6 days) but observed different patterns at the later lag times. There appeared to be infection specific patterns of gene expression when comparing patients with RVI, RBI and combined respiratory infection. Overall, this analysis suggests that the mechanistic effects of air pollution on the pathogenesis of respiratory infection may be pollutant, timing, and infection specific.

Discerning the effect of air pollution on the normal immune response to respiratory infection is made more difficult by limitations of our use of an epidemiological design, and by the overlapping effects of both air pollution and respiratory infection on the human immune response.

To date, it is not clear by what mechanism(s) short term air pollution exposures contribute to diagnosed respiratory infection. Independent of air pollution exposure, RVIs can disrupt epithelial barriers^23–25^, activate an inflammatory cascade mediated by nuclear factor kappa-light-chain-enhancer of activated B cells (NF-κB)^26^, and activate key antiviral proteins, including Type I (e.g. interferon beta [IFN-β]) and Type II (e.g. interferon gamma [IFN-γ]) interferons)^27,28^.

Differentiating RVI from RBI using gene expression is an area of active research^29^. In a study by Tsalik et al.^29^, three externally validated, well performing (AUC 0.90–0.99), host-response classifiers were described for non-infectious disease, bacterial and viral respiratory infection respectively. Though direct comparison to genes in our study was limited, the overall theme of distinct gene profiles for viral and bacterial infection and a variable (heterogeneous) pattern for combined viral and bacterial infection was similar to the patterns observed in the exploratory heatmap (without pollution modeling) of our study (Fig. 2). In another study focused on individual genes, found that a single gene (IFI27) is able to differentiate influenza from RBI^30^. IFI27 was also identified in the Suarez study^22^ as a classifier of RBI vs. RVI and was observed in our brief replication analysis Supplemental Table S4. While distinct gene expression can exist between RVI and RBI, air pollution itself can also illicit a strong immune response independent of respiratory infection.

Independent of respiratory infection, air pollution exposure is known to broadly lead to immune dysregulation in cell and animal models through pro-inflammatory changes to lung epithelia, dysregulation of cell signaling pathways and direct effects on immune cells including macrophages, dendritic cells and granulocytes^31^. Specifically, in response to diesel exhaust particles, a NF-κB mediated inflammatory cascade is thought to occur within the lung epithelium^10^. This response has the potential to disrupt the tight junctions between epithelial cells, thereby increasing the risk for viral or bacterial penetration and subsequent infection^9,11^. We did not observe many inflammatory pathways associated with air pollution and only observed two individual inflammatory related genes in our study. ADORA1 participates in the activation of monocytes, which leads to a pro-inflammatory response. HERC5 was another example of a gene upregulated by endothelial inflammation that was differentially expressed between types of infections in our study. The paucity of observed inflammatory changes may be related to the overall low concentrations of ambient air pollution in the Rochester area compared to other more heavily polluted areas.

In terms of immune effects, decreased levels of IFN-γ (important for macrophage activation) were observed in the peripheral blood of mice exposed to traffic pollution in China^12^, and in the peripheral blood of humans exposed to diesel exhaust^32^. Further research has also observed dysregulation of the epithelial cell junction, respiratory microbiome and cytokine response as additional factors increasing pathogenic virulence in the setting of PM exposure^33^. Our analysis suggests that while BC is associated with upregulation of type 1 interferon related pathway in the two weeks prior to infection, there may also be a component of immune suppression associated with exposure to traffic pollution in the later lag periods. Specifically, in the pathway analysis (Table 3), BC was associated with upregulation with numerous immune related pathways at the 0–6 lag period and downregulation of natural killer cell activity and antigen processing in the 14–20 day lag period. BC may have unique health effects when compared to other PM due to its physical shape as a chain aggregate particle. This shape provides a large surface area and concavity between intersecting spheres (most other particles are convex/spherical) that improves the ability for BC to serve as a transport vector for other chemicals or possibly infectious organisms into the body^34^. UFP also was associated with upregulation of a viral related pathway at the 0–6 lag day period and suppression of multiple immune related pathways at the 7–13 lag day lag period. Though speculative, these finding suggest that for BC and UFP, an inflection point may exist where the effects of air pollution alone (lag days 13–28) are then comingled with the effect of acute infection during the incubation period in the 7 days preceding infection. In contrast to BC and UFP, PM_2.5_ was associated with suppression of multiple immune related pathways at the 0–6 lag day period but had no associations with immune pathways at later lag periods. Though the etiology of the gene suppression from PM_2.5_ is not clear, it may suggest that effects on immunity may be pollutant specific in addition to being timing specific. The relative importance of the directionality and timing of these immune changes for specific pollutants (and considering the composition of pollutants like PM_2.5_) in the pathogenesis of respiratory infection deserves further study in a prospective manner.

Despite our study population preceding the current COVID-19 pandemic, our participants had a high prevalence of several comorbidities that are risk factors to severe COVID-19 illness including diabetes, COPD, smoking and obesity^35^. A deficiency in the type 1 interferon response has been hypothesized to be a risk factor for a severe clinical course of COVID-19^36^. Our study observed a difference in type 1 interferon expression when comparing RVI (high expression) and RBI (low expression) in the week prior to hospitalization (Fig. 3). The most distinct differences in gene expression for type 1 interferon between RVI and RBI corresponded to the highest concentrations of black carbon. We observed that one cluster of genes within the Type 1 Interferon pathway appeared to drive the association between air pollution and gene expression. Determining the potential gene pathways and individual genes involved in the air pollution/respiratory infection association is a key area of research for the current and future pandemics. A further benefit of improved knowledge of the risk of specific air pollutants and respiratory infections could be the ability to make real time policy changes (e.g. diesel traffic modifications) during a pandemic to reduce pathogen virulence.

In addition to the immune specific pathways, there were two additional general pathways broadly related to protein folding and iron homeostasis, which were associated with changes in air pollution. All pollutants were associated with three pathways related to the endoplasmic reticulum (ER), an organelle central to protein synthesis (e.g. cytokines or other immune proteins) and transport in the body^37^. (Table 3) In a prior in vitro study of PM exposure to bronchial epithelial cells, PM increased stress on the ER (upregulation) that lead to a deleterious unfolded protein response (UPR)^38^. Influenza has also been observed to cause similar dysfunction in the ER^39^. This can impair the function of cells central to innate immunity in the lung including bronchial epithelial cells^40^, and also has the potential to lead to dysregulation in the synthesis and transport of other immune related proteins. Aside from AMP, all other pollutant/ER related pathway associated were observed at the 7–27 day lag period, with no association observed in the 0–6 day lag period. This may suggest that the ER (protein folding) related dysregulation precedes the time point of acute infection for infections with incubation periods under 7 days (e.g. Influenza)^41^. While all other pollutants led to upregulation of ER related pathways, PM_2.5_ was associated with suppression of the ER pathways. While both upregulation and suppression could lead to dysregulation, upregulation may be more detrimental due to the risk of the UPR.

Iron homeostasis plays an important role in the clinical course of RBI^42,^ and is increasingly recognized as an important factor in RVI^43^ as well. Air pollution is known to induce a relative iron deficiency through dysregulation of iron homeostasis through mechanisms of chelation and/or sequestration^44^. Aside from BC, all other pollutants were observed to upregulate multiple pathways of iron homeostasis (Table 3). The association between multiple pollutants and an upregulation of iron related pathways is consistent with the relative iron deficient state induced by air pollution. Intracellular iron deficiency can lead to an increased oxidative state and inflammation within the host. Whether the observed changes in iron homeostasis are protective (immune priming), deleterious (immunosuppressive), or related to changes in the proportion of blood cells remains unclear. The effect of air pollution on iron homeostasis pathways in viral infection is deserving of further study.

### Limitations

Our study results should be interpreted in light of several limitations. First, our study focused on a small group of severely ill patients requiring hospitalization, though the final specific strata of severity within the hospital were not recorded as participants were enrolled upon hospital arrival. Our cohort was also older with multiple comorbidities, potentially limiting the generalizability to younger patients with respiratory infection. Second, the lack of a control population limited the pathway and LIMMA analysis to comparisons between types of infection. Furthermore, in the use of alternative gene-list based gene set enrichment algorithms, such as Enricher^45,46^, was not possible in the study due to the relatively small number of marginally statistically significant genes identified. Third, this analysis was not able to correct for the blood cell proportions in the peripheral whole blood of our samples. In theory the lack of inclusion of blood cell proportions should only minimally change the magnitude of effect estimates and would result in a larger standard error and reduced statistical significance. Fourth, the epidemiological design does not allow for causal link between air pollution and immune response to respiratory infection. Fifth, as there was no external validation with gene sets outside of our study, the generalizability of our findings are reduced. Sixth, there was likely an element of exposure misclassification given central site monitor estimated pollution, which likely reduced the magnitude of the observed effects. Finally, Rochester, NY has a relatively low average concentration of PM air pollution so generalizability to areas of higher pollution may be limited if dose thresholds exist in the pathogenic response to PM. Future studies can improve exposure assessment by using land use regression techniques, account for multipollutant mixtures and improve overall generalizability by including patients with mild infection and non-infected controls.

## Conclusions

Overall, this epidemiological study suggests that combustion related pollution, particularly BC, is associated with changes in gene expression within innate immune pathways. Increased concentrations in the majority of pollutants also appear to correspond to changes in expression to protein folding and iron homeostasis. Distinct from other pollutants, PM_2.5_ was associated with downregulation of immune and protein folding pathways. The relatively low pollution in the study region may explain the lack of inflammatory changes accompanying the changes in the immune pathways. Future controlled exposure studies informed by epidemiological studies are needed to further explore the relationship between inflammatory and immune responses to particulate air pollution in patients with respiratory infection.

## Methods

We used existing data from 111 patients originally enrolled in the study by Falsey et al.^47^, who underwent transcriptional profiling as detailed in the study by Suarez et al.^22^ included adults over the age of 21 years with symptoms compatible with acute respiratory tract infection admitted through the emergency department at Rochester General Hospital (RGH), Rochester, NY from 2008 to 2011. As detailed in Falsey et al.^47^, each patient was assigned an admitting diagnosis by a pulmonary specialist after examination of each subject and review of laboratory, microbiologic and radiographic data. All ethical approvals, guidelines and consent were provided in this previous study. Subjects had comprehensive microbiologic testing and cases were adjudicated by specialists as viral alone, bacterial alone, or mixed viral-bacterial infection. From this population, 1–3 ml of peripheral whole blood RNA was collected from 118 patients in Tempus tubes and hybridized using an Illumina Human HT-12 v4 BeadChip kit. Transcripts from the Illumina GenomeStudio based analysis were included if they were present in 10% or more of the samples and if they exhibited a minimum of a twofold expression change. As 7 of the 118 patients had missing pollution data, we analyzed the data of 111 patients who had a transcriptional analysis of peripheral blood performed in our current study on the association between air pollution and gene expression.

### Air pollution data

Ambient air pollution concentrations were measured at a central site monitor in Rochester, NY, and all patients living in Monroe County, NY were assigned pollutant concentrations from this monitor. The daily ambient air pollutant concentrations in the 28 days prior to the date of each participant’s hospital admission were matched to each participant, as an estimate of the patient’s air pollution exposure in those 28 days. Specifically, measurements of particle number concentrations in the size range of 10–500 nm are made continuously and sequentially at the New York State Department of Environmental Conservation (NYS DEC) site in Rochester, NY^48^. From 2004 to the present, measurements have been made at the NYS DEC primary site (latitude 43°09′56″ N, longitude 77°33′15″ W) on the eastside of Rochester, NY. This sampling site is close to two major interstates (I-490 and I-590) as well as NY route 96, a major route carrying traffic traveling to and from downtown Rochester. Hourly PM_2.5_ mass, wind speed and wind direction, ambient temperature and relative humidity are also measured at the above-mentioned site. Size distribution measurements are made using a scanning mobility particle sizer (SMPS, TSI Inc.) system consisting of an electrostatic classifier (TSI model 3071), with an impactor having an orifice size of 0.0457 cm, an 85Kr aerosol neutralizer (TSI model 3077), and a condensation particle counter (CPC; TSI model 3010). The size range bounds are 10.4 nm (lower) and 0.542 µm (upper) leading to measurement of mid-point particle sizes ranging from 11.1 nm to 0.47 µm (32 channels per decade) at a total scan time of 5 min per sample. Routine maintenance such as calibrating rates is performed once a week to ensure that the system is functioning properly. PM_2.5_ is measured with a TEOM (model 1400ab, Thermo Fisher Scientific Inc., USA). Black carbon (BC) was measured with a 2-wavelength aethalometer. Delta-C (DC) is the difference between BC measured at 370 and 880 nm and has been shown to be a marker for biomass burning^49^. Pollution measurements were taken from Nov 1st, 2008 to May 31st, 2011.

### Microarray data acquisition and processing

This dataset, GSE60244 on the Gene Expression Omnibus, contains background corrected, non-normalized whole-blood genome data from microarrays run on the Illumina HumanHT-12 V4.0 expression BeadChip platform. The reported detection p-values were used to infer the mean and variance of the negative control probe intensities in order to perform background correction using the normal exponential convolution model. This method prevents negative values that arise from subtraction-based background correction. Quantile normalization was used after background correction in order to minimize variation between arrays. Specifically, we used the neqc function in LIMMA to perform adaptive background correction based on each array in order to account for background intensity around each feature and control for variability between arrays. The neqc function uses both negative and positive controls for normalization ^50^. The probes were matched to gene names using the annotation package IluminaHumanV4 ^51^. Unidentified and non-detected genes were removed.

### Statistics

#### Exploratory analysis

After preprocessing, the top 150 high variance genes were selected for initial exploratory analyses. The selected expression values were centered and scaled to have mean zero and variance one. We performed hierarchical clustering with Euclidean distance and complete linkage on both the genes and samples. The resulting sample dendrogram was qualitatively compared to ambient air pollution levels in the weeks prior to infection diagnosis obtained from a prior study^22^. Finally, the ambient pollution values for each patient were overlaid from the day 0 to day 6 time period preceding diagnosis of infection. The program ComplexHeatmap was used to generate the heatmaps in this study^52^.

### Individual gene analysis

The Linear Models for MicroArray (LIMMA) package^53^ in R (version 4.03)^54^ was used to test hypotheses about the effect of ambient air pollution levels in the weeks prior to infection diagnosis on patients’ gene expression using all 47,231 probes available in the microarray platform.

Each of the five pollutant exposures was tested individually by fitting a separate linear model for each of the four exposure time intervals: 0–6 days, 7–13 days, 14–20 days, and 21–27 days prior to date of diagnosis. This resulted in fitting a total of 20 models of the following form:

$${E[Y}_{ij}]={\beta }_{0j}+{\beta }_{1j}I({Viral}_{i})+{\beta }_{2j}I({Coinfection}_{i})+{\beta }_{3j}I({Female}_{i})+{\beta }_{4j}{x}_{i}^{pollutant}$$

Here ${Y}_{ij}$ is the gene expression in subject i for gene probe *j*. Since no pollution exposure data was available for the gene expression control group, the bacterial infection group was set as the baseline category; therefore, the difference in expected gene expression between viral and bacterial infection and between coinfection and bacterial infection are ${\beta }_{1j}$ and ${\beta }_{2j}$, respectively. The difference in expected gene expression between female and male subjects is ${\beta }_{3j}$. Sex was selected as a covariate of interest as it was predictive of respiratory viral infection and had a potential effect on gene expression. None of the other covariates tested, including race, smoking, COPD, diabetes, congestive heart failure, white blood cell count, oxygen requirement, chronic renal failure, statin and obesity were predictive of respiratory viral infection and were therefore not included in the model. Ambient pollutant levels measured for AMP, PM _2.5_, UFP, DC, and BC in four different time lags are each used separately as the pollution value ${x}_{i}^{pollutant}$, and ${\beta }_{4j}$ is the change in expected gene expression for a one unit increase in a given pollutant. Standard errors for these coefficients were calculated using the empirical Bayes method^55^ central to the LIMMA method. Differential expression was determined by using a false discovery rate (FDR) threshold of 0.1.

### Pathway analysis

In order to test the effect of pollutants on gene pathways we used the CAMERA algorithm^56^. CAMERA implements a competitive gene set test comparing each pathway against all other genes not in the pathway. Such tests focus on identifying the most important biological processes relative to all other processes. Gene-wise moderated t-statistics are used as in LIMMA, but here the goal is to determine if the mean of the gene-wise statistics differ between the pathway of interest and all other genes. The key feature of CAMERA is that it accounts for inter-gene correlation in order to better control type I error. In this work, we used a false discovery rate threshold of 0.1.

Pathway definitions came from the Molecular Signatures Database version 7.1 GO: Gene Ontology gene sets listing of 10,192 pathways. The pathways defined here are derived from the Gene Ontology (GO) resource, and they were compiled into R data files which mapped probes to gene symbols, which were subsequently used to define pathway membership. We cross referenced the significant LIMMA results against the significant CAMERA results to limit our scope to only the pathways found significant in CAMERA which also contained individual genes found to be significant in the LIMMA analysis. From this subset of 448 pathways, we chose those known to be associated with infection and clustered those gene sets as in the unsupervised analysis to visually examine trends across patients and expression levels compared with pollutant levels.

## Supplementary Information

Supplementary Information 1.

Supplementary Information 2.

## References

[1] Xu J, Kochanek KD, Murphy SL, Arias E (2012). Mortality in the United States. NCHS Data Brief.

[2] Croft DP, Zhang WJ, Lin S, Thurston SW, Hopke PK, Masiol M (2019). The association between respiratory infection and air pollution in the setting of air quality policy and economic change. Ann. Am. Thorac. Soc..

[3] Ciencewicki J, Jaspers I (2007). Air pollution and respiratory viral infection. Inhal. Toxicol..

[4] Horne BD, Joy EA, Hofmann MG, Gesteland PH, Cannon JB, Lefler JS (2018). Short-term elevation of fine particulate matter air pollution and acute lower respiratory infection. Am. J. Respir. Crit. Care Med..

[5] Pirozzi CS, Jones BE, VanDerslice JA, Zhang Y, Paine R, Dean NC (2018). Short-term air pollution and incident pneumonia: A case-crossover study. Ann. Am. Thorac. Soc..

[6] Krall JR, Mulholland JA, Russell AG, Balachandran S, Winquist A, Tolbert PE (2017). Associations between source-specific fine particulate matter and emergency department visits for respiratory disease in four US cities. Environ. Health Perspect..

[7] WHO. *Ambient Air Pollution: A Global Assessment of Exposure and Burden of Disease*. http://www.who.int/phe/publications/air-pollution-global-assessment/en/ (2016).

[8] CDC. *Stats of the State of New York 2018* [updated 4/10/2018]. Accessed 1 Nov 2019.

[9] Bayram H, Rusznak C, Khair OA, Sapsford RJ, Abdelaziz MM (2002). Effect of ozone and nitrogen dioxide on the permeability of bronchial epithelial cell cultures of non-asthmatic and asthmatic subjects. Clin. Exp. Allergy..

[10] Weng CM, Lee MJ, He JR, Chao MW, Wang CH, Kuo HP (2018). Diesel exhaust particles up-regulate interleukin-17A expression via ROS/NF-kappaB in airway epithelium. Biochem. Pharmacol..

[11] Yu XY, Takahashi N, Croxton TL, Spannhake EW (1994). Modulation of bronchial epithelial cell barrier function by in vitro ozone exposure. Environ. Health Perspect..

[12] Yang J, Chen Y, Yu Z, Ding H, Ma Z (2018). Changes in gene expression in lungs of mice exposed to traffic-related air pollution. Mol. Cell. Probes.

[13] Sigaud S, Goldsmith CA, Zhou H, Yang Z, Fedulov A, Imrich A (2007). Air pollution particles diminish bacterial clearance in the primed lungs of mice. Toxicol. Appl. Pharmacol..

[14] Lee GI, Saravia J, You D, Shrestha B, Jaligama S, Hebert VY (2014). Exposure to combustion generated environmentally persistent free radicals enhances severity of influenza virus infection. Part. Fibre Toxicol..

[15] Borcherding JA, Chen H, Caraballo JC, Baltrusaitis J, Pezzulo AA, Zabner J (2013). Coal fly ash impairs airway antimicrobial peptides and increases bacterial growth. PLoS ONE.

[16] Harrod KS, Jaramillo RJ, Rosenberger CL, Wang SZ, Berger JA, McDonald JD (2003). Increased susceptibility to RSV infection by exposure to inhaled diesel engine emissions. Am. J. Respir. Cell Mol. Biol..

[17] Jaspers I, Ciencewicki JM, Zhang W, Brighton LE, Carson JL, Beck MA (2005). Diesel exhaust enhances influenza virus infections in respiratory epithelial cells. Toxicol. Sci..

[18] Bauer RN, Diaz-Sanchez D, Jaspers I (2012). Effects of air pollutants on innate immunity: The role of Toll-like receptors and nucleotide-binding oligomerization domain-like receptors. J. Allergy Clin. Immunol..

[19] Smyth T, Veazey J, Eliseeva S, Chalupa D, Elder A, Georas SN (2020). Diesel exhaust particle exposure reduces expression of the epithelial tight junction protein Tricellulin. Part. Fibre Toxicol..

[20] Croft DP, Zhang W, Lin S, Thurston SW, Hopke PK, van Wijngaarden E (2020). Associations between source-specific particulate matter and respiratory infections in New York state adults. Environ. Sci. Technol..

[21] Office USGP. *Electronic Code of Federal Regulations 2021* [updated 3/11/2021]. https://www.ecfr.gov/cgi-bin/text-idx?node=pt40.2.50&rgn=div5#se40.2.50_17. Accessed 13 mar 2021.

[22] Suarez NM, Bunsow E, Falsey AR, Walsh EE, Mejias A, Ramilo O (2015). Superiority of transcriptional profiling over procalcitonin for distinguishing bacterial from viral lower respiratory tract infections in hospitalized adults. J. Infect. Dis..

[23] Comstock AT, Ganesan S, Chattoraj A, Faris AN, Margolis BL, Hershenson MB (2011). Rhinovirus-induced barrier dysfunction in polarized airway epithelial cells is mediated by NADPH oxidase 1. J. Virol..

[24] Rezaee F, DeSando SA, Ivanov AI, Chapman TJ, Knowlden SA, Beck LA (2013). Sustained protein kinase D activation mediates respiratory syncytial virus-induced airway barrier disruption. J. Virol..

[25] Coyne CB, Shen L, Turner JR, Bergelson JM (2007). Coxsackievirus entry across epithelial tight junctions requires occludin and the small GTPases Rab34 and Rab5. Cell Host Microbe..

[26] Garcia MA, Gallego P, Campagna M, Gonzalez-Santamaria J, Martinez G, Marcos-Villar L (2009). Activation of NF-kB pathway by virus infection requires Rb expression. PLoS ONE.

[27] Li SF, Gong MJ, Zhao FR, Shao JJ, Xie YL, Zhang YG (2018). Type I interferons: Distinct biological activities and current applications for viral infection. Cell. Physiol. Biochem..

[28] Platanias LC (2005). Mechanisms of type-I- and type-II-interferon-mediated signalling. Nat. Rev. Immunol..

[29] Tsalik EL, Henao R, Nichols M, Burke T, Ko ER, McClain MT (2016). Host gene expression classifiers diagnose acute respiratory illness etiology. Sci. Transl. Med..

[30] Tang BM, Shojaei M, Parnell GP, Huang S, Nalos M, Teoh S (2017). A novel immune biomarker IFI27 discriminates between influenza and bacteria in patients with suspected respiratory infection. Eur. Respir. J..

[31] Glencross DA, Ho T-R, Camiña N, Hawrylowicz CM, Pfeffer PE (2020). Air pollution and its effects on the immune system. Free Radical Biol. Med..

[32] Estrella B, Naumova EN, Cepeda M, Voortman T, Katsikis PD, Drexhage HA (2019). Effects of air pollution on lung innate lymphoid cells: Review of in vitro and in vivo experimental studies. Int. J. Environ. Res. Public Health..

[33] Pompilio A, Di Bonaventura G (2020). Ambient air pollution and respiratory bacterial infections, a troubling association: Epidemiology, underlying mechanisms, and future challenges. Crit. Rev. Microbiol..

[34] World Health Organization (2012). Health Effects of Black Carbon.

[35] Ejaz H, Alsrhani A, Zafar A, Javed H, Junaid K, Abdalla AE (2020). COVID-19 and comorbidities: Deleterious impact on infected patients. J. Infect. Public Health.

[36] Hadjadj J, Yatim N, Barnabei L, Corneau A, Boussier J, Smith N (2020). Impaired type I interferon activity and inflammatory responses in severe COVID-19 patients. Science.

[37] Schwarz DS, Blower MD (2016). The endoplasmic reticulum: Structure, function and response to cellular signaling. Cell. Mol. Life Sci..

[38] Watterson TL, Hamilton B, Martin R, Coulombe RA (2009). Urban particulate matter causes ER stress and the unfolded protein response in human lung cells. Toxicol. Sci..

[39] Zhang K, Kaufman RJ (2006). The unfolded protein response. Neurology.

[40] Chen ACH, Burr L, McGuckin MA (2018). Oxidative and endoplasmic reticulum stress in respiratory disease. Clin. Transl. Immunol..

[41] Lessler J, Reich NG, Brookmeyer R, Perl TM, Nelson KE, Cummings DA (2009). Incubation periods of acute respiratory viral infections: A systematic review. Lancet Infect Dis..

[42] Gomes AC, Moreira AC, Mesquita G, Gomes MS (2018). Modulation of iron metabolism in response to infection: Twists for all tastes. Pharmaceuticals.

[43] Liu W, Zhang S, Nekhai S, Liu S (2020). Depriving iron supply to the virus represents a promising adjuvant therapeutic against viral survival. Curr. Clin. Microbiol. Rep..

[44] Ghio AJ, Soukup JM, Dailey LA, Madden MC (2020). Air pollutants disrupt iron homeostasis to impact oxidant generation, biological effects, and tissue injury. Free Radic. Biol. Med..

[45] Chen EY, Tan CM, Kou Y, Duan Q, Wang Z, Meirelles GV (2013). Enrichr: Interactive and collaborative HTML5 gene list enrichment analysis tool. BMC Bioinform..

[46] Kuleshov MV, Jones MR, Rouillard AD, Fernandez NF, Duan Q, Wang Z (2016). Enrichr: A comprehensive gene set enrichment analysis web server 2016 update. Nucleic Acids Res..

[47] Falsey AR, Becker KL, Swinburne AJ, Nylen ES, Formica MA, Hennessey PA (2013). Bacterial complications of respiratory tract viral illness: a comprehensive evaluation. J. Infect. Dis..

[48] Masiol M, Squizzato S, Chalupa DC, Utell MJ, Rich DQ, Hopke PK (2018). Long-term trends in submicron particle concentrations in a metropolitan area of the northeastern United States. Sci. Total Environ..

[49] Wang Y, Hopke PK, Rattigan OV, Chalupa DC, Utell MJ (2012). Multiple-year black carbon measurements and source apportionment using delta-C in Rochester, New York. J. Air Waste Manag. Assoc..

[50] Shi W, Oshlack A, Smyth GK (2010). Optimizing the noise versus bias trade-off for Illumina whole genome expression BeadChips. Nucleic Acids Res..

[51] Dunning, M., Lynch, A. & Eldridge, M. *Illuminahumanv4. db: Illumina HumanHT12v4 Annotation Data (chip illuminaHumanv4)*. (R package version, 2015).

[52] Gu Z, Eils R, Schlesner M (2016). Complex heatmaps reveal patterns and correlations in multidimensional genomic data. Bioinformatics.

[53] Ritchie ME, Phipson B, Wu D, Hu Y, Law CW, Shi W (2015). limma powers differential expression analyses for RNA-sequencing and microarray studies. Nucleic Acids Res..

[54] R Foundation for Statistical Computing.* R: A Language and Environment for Statistical Computing*. (R Foundation for Statistical Computing, 2020). https://www.R-project.org/.

[55] Law CW, Chen Y, Shi W, Smyth GK (2014). voom: Precision weights unlock linear model analysis tools for RNA-seq read counts. Genome Biol..

[56] Wu D, Smyth GK (2012). Camera: A competitive gene set test accounting for inter-gene correlation. Nucleic Acids Res..

