# Supplementary material for: The effect of air pollution on the transcriptomics of the immune response to respiratory infection
Source: Sci Rep. 2021 Sep 30;11:19436. doi: 10.1038/s41598-021-98729-8 (PMC8484285; doi:10.1038/s41598-021-98729-8)

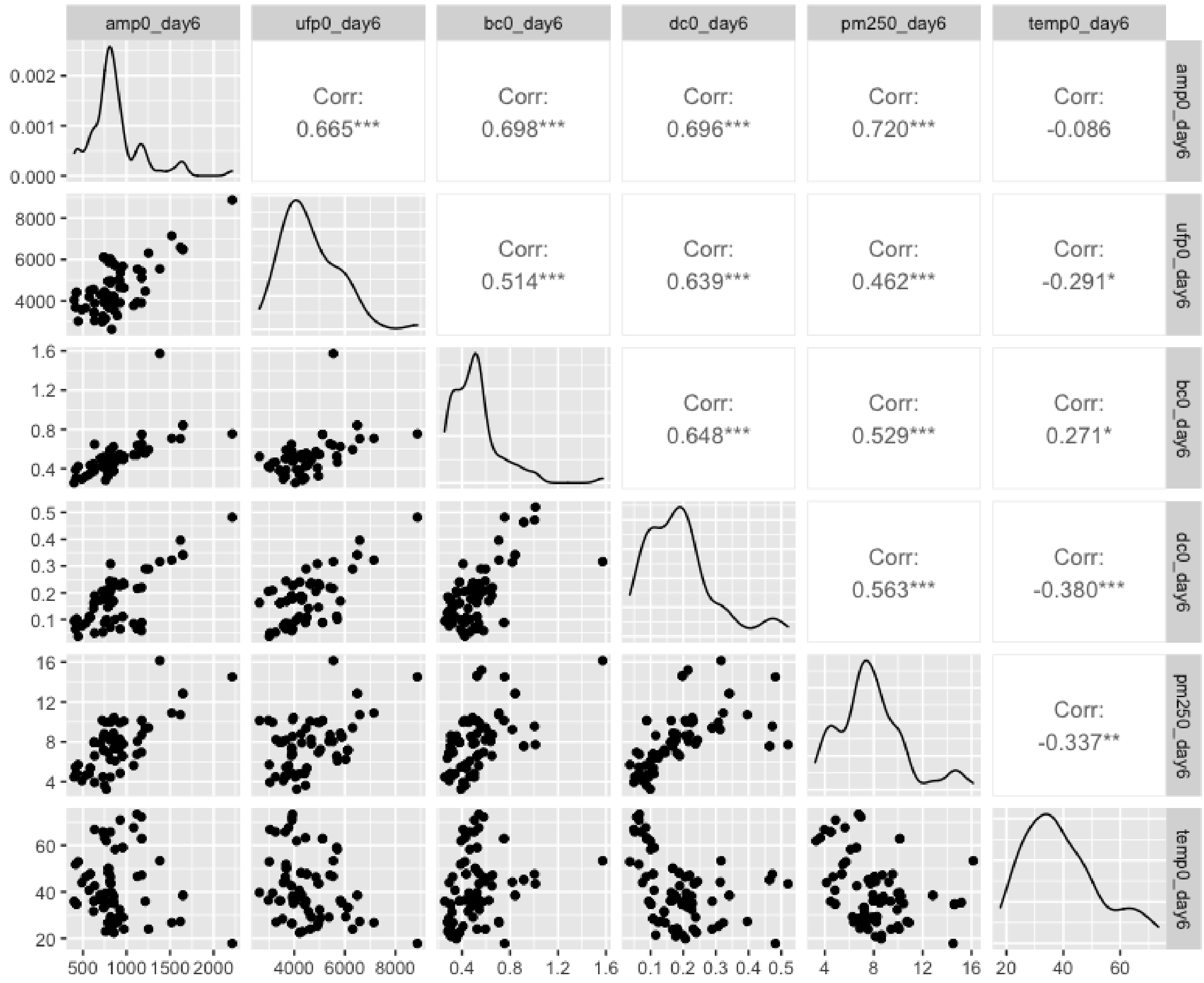

Figure S1: Correlation matrix of pollutants and temperature at the 0-6 day lag period.
* p < 0.05 ** p < 0.01 *** p < 0.001

Figure S2: Gene expression heatmap of the Protoporphyrinogen IX Biosynthetic Process pathway sorted by diagnosis and concentration of Delta-C during lag days 0-6

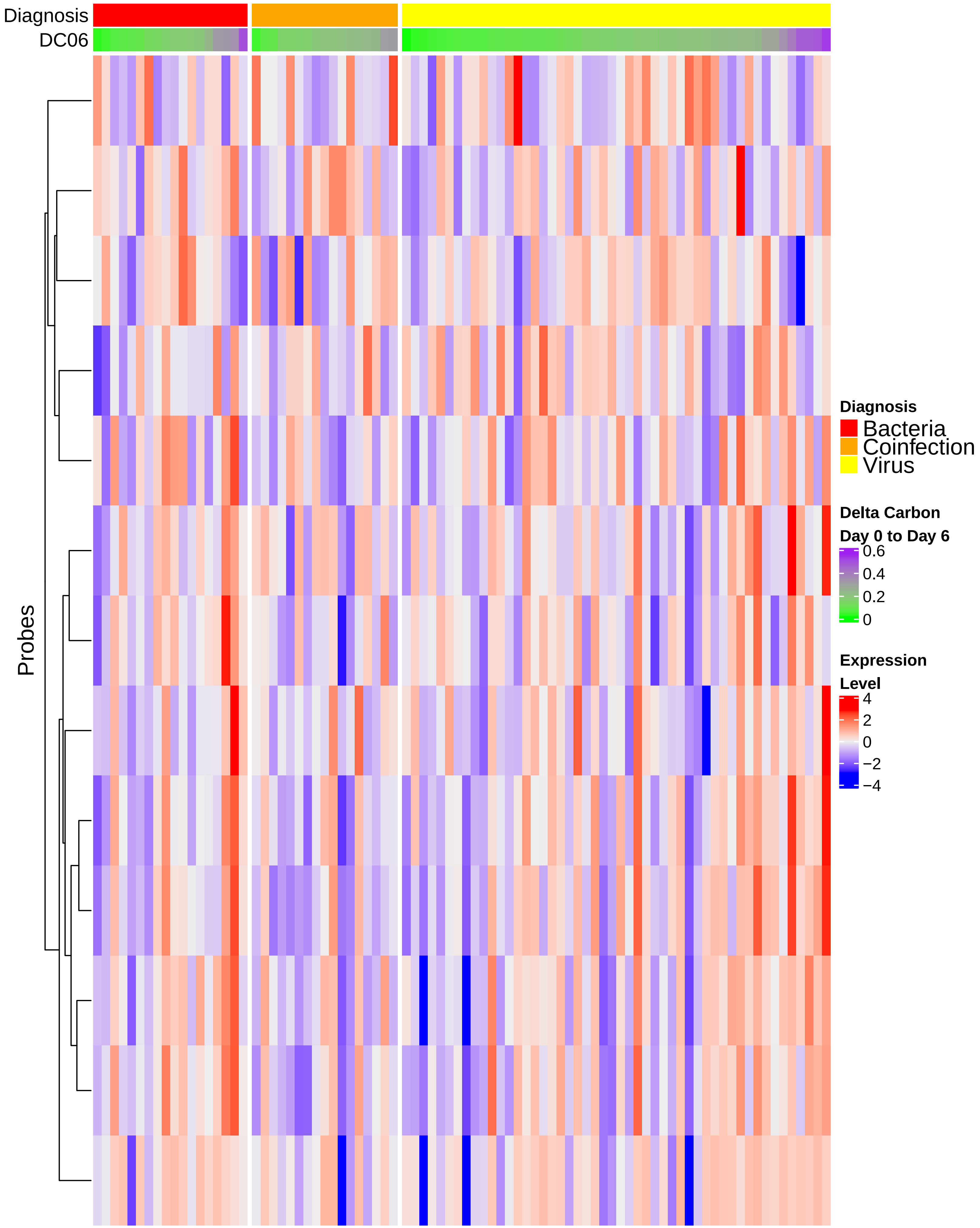

Figure S3: Gene expression heatmap of the Protoporphyrinogen IX Biosynthetic Process pathway sorted by diagnosis and concentration of Delta-C during lag days 21-27

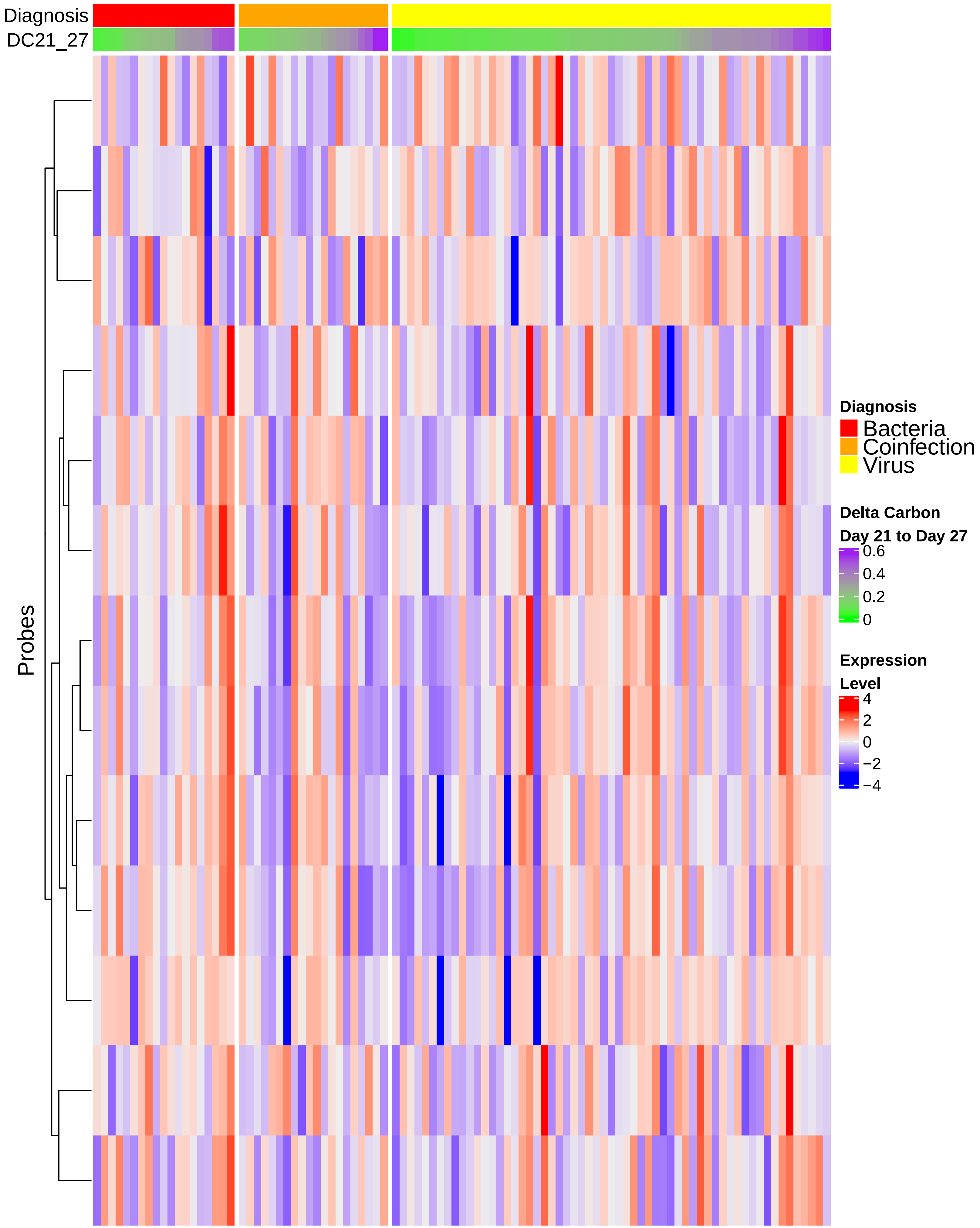

Figure S4: Gene expression heatmap of the Structural Constituent of Ribosome pathway sorted by diagnosis and concentration of Delta-C during lag days 7-13

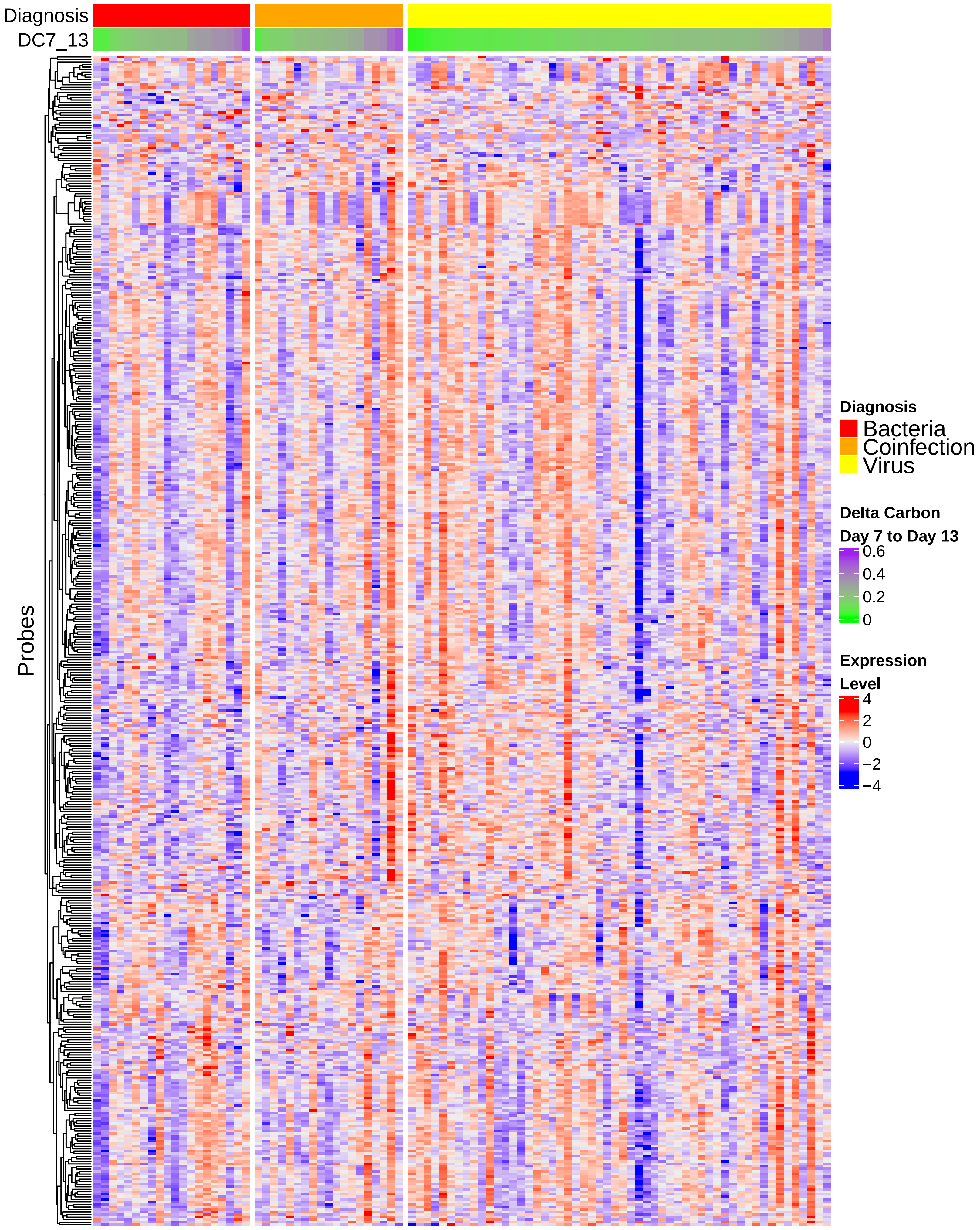

Figure S5: Gene expression heatmap of the Structural Constituent of Ribosome pathway sorted by diagnosis and concentration of Delta-C during lag days 14-20

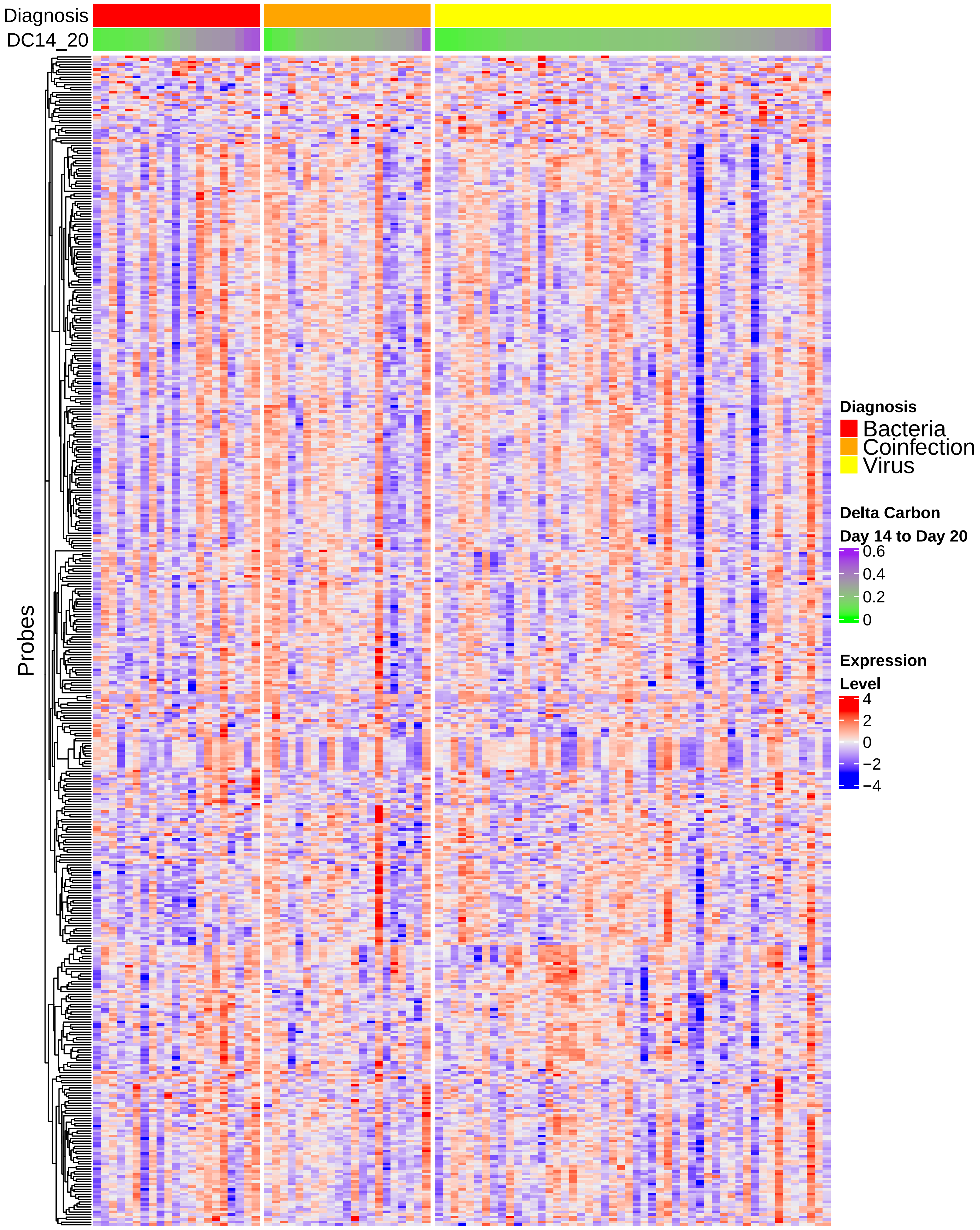

Figure S6: Gene expression heatmap of the Viral Gene Expression pathway sorted by diagnosis and concentration of Delta-C during lag days 7-13

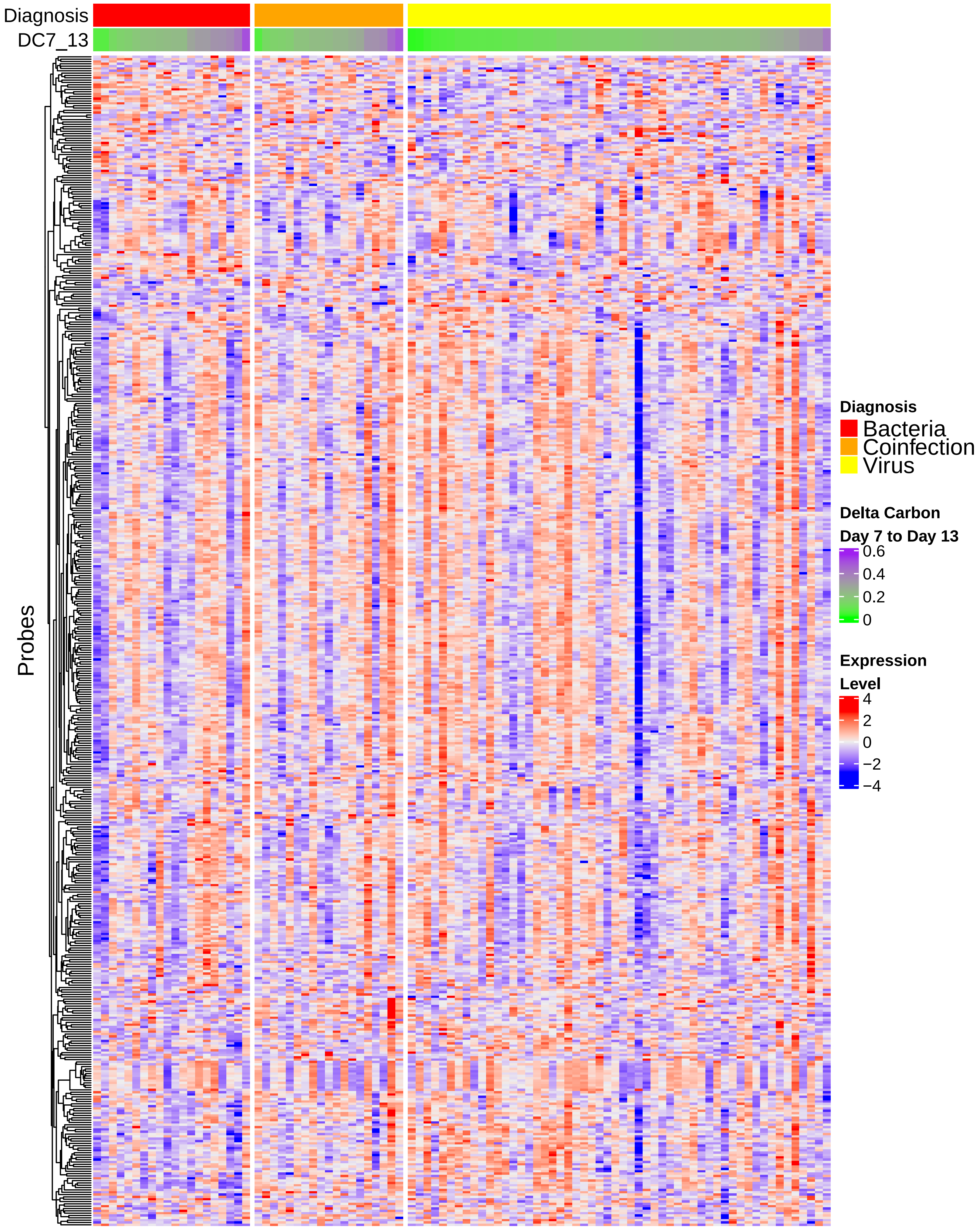

Figure S7: Gene expression heatmap of the Viral Gene Expression pathway sorted by diagnosis and concentration of Delta-C during lag days 14-20

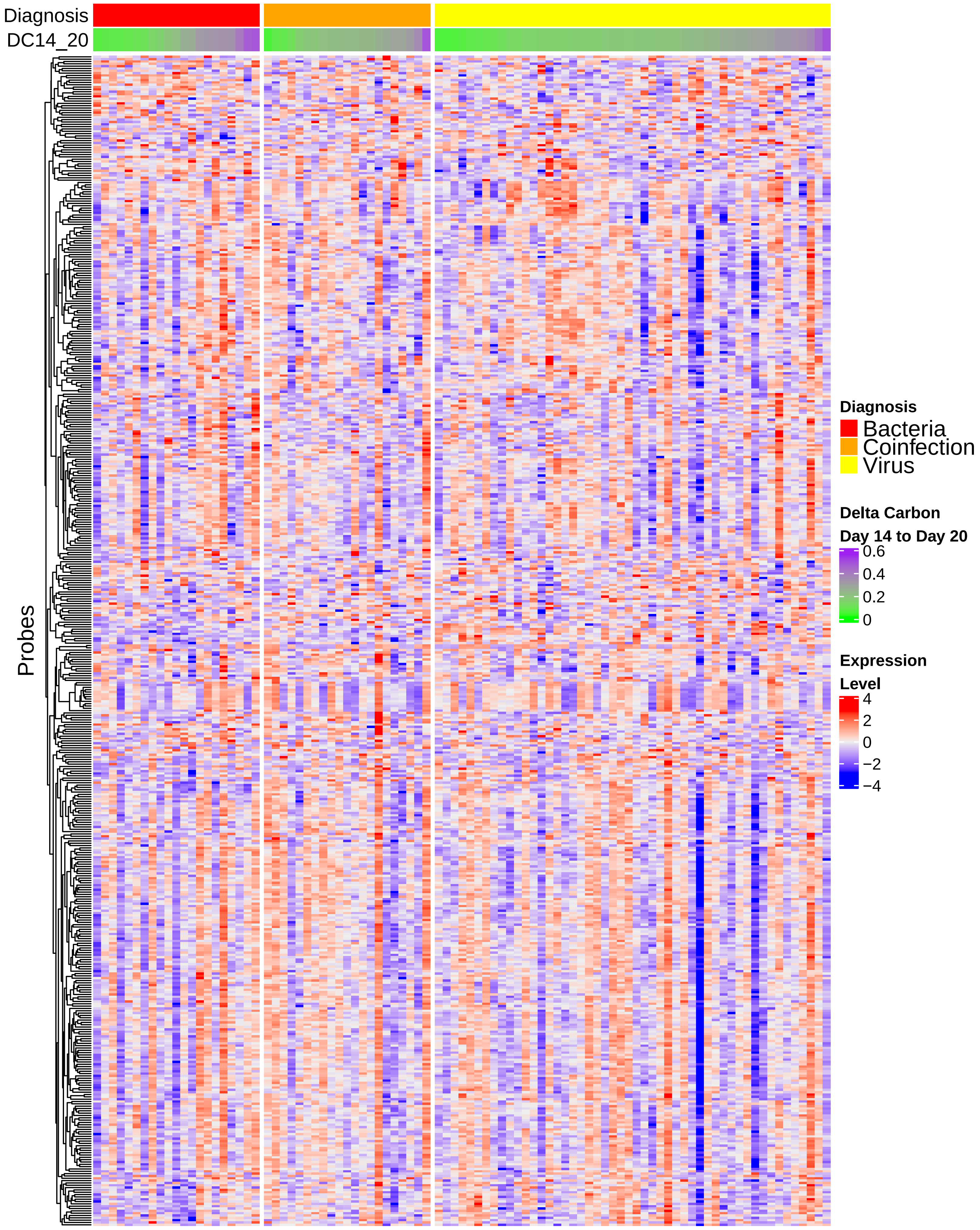

Figure S8: Gene expression heatmap of the Protoporphyrinogen IX Biosynthetic Process pathway sorted by diagnosis and concentration of $PM_{2.5}$ during lag days 0-6

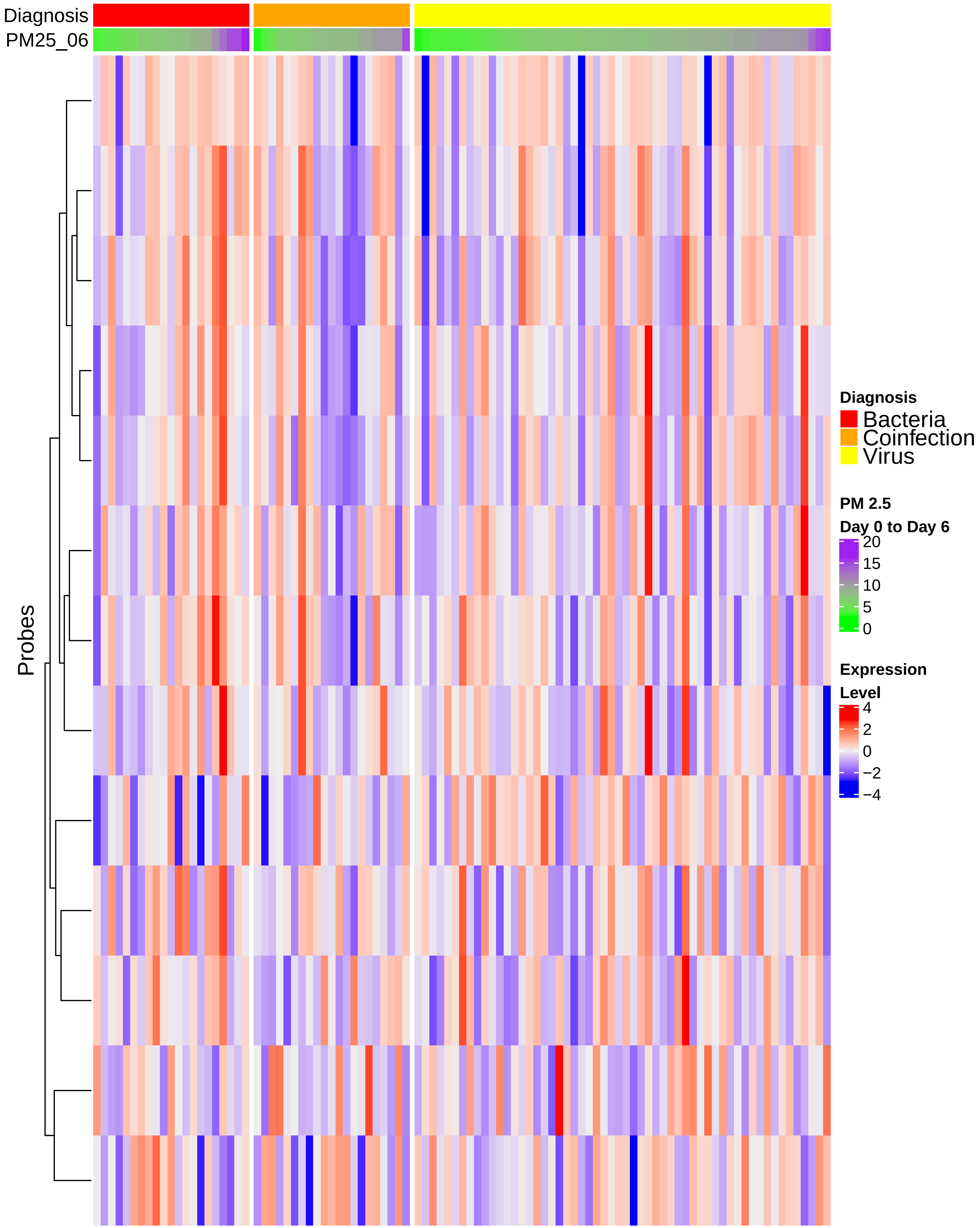

Figure S9: Gene expression heatmap of the Protoporphyrinogen IX Biosynthetic Process pathway sorted by diagnosis and concentration of $PM_{2.5}$ during lag days 14-20

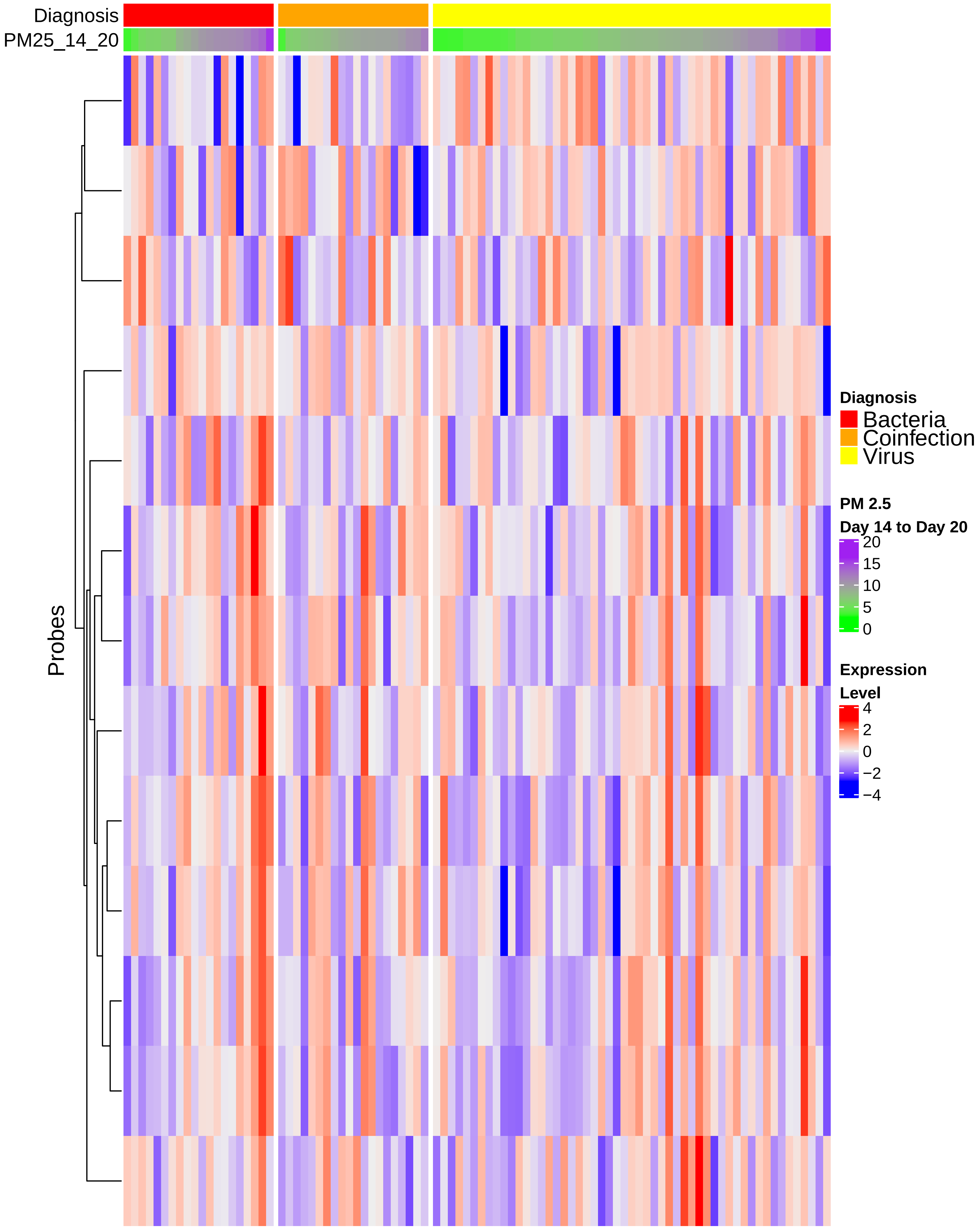

Figure S10: Gene expression heatmap of the Viral Gene Expression pathway sorted by diagnosis and concentration of Ultrafine particles (UFP) during lag days 14-20

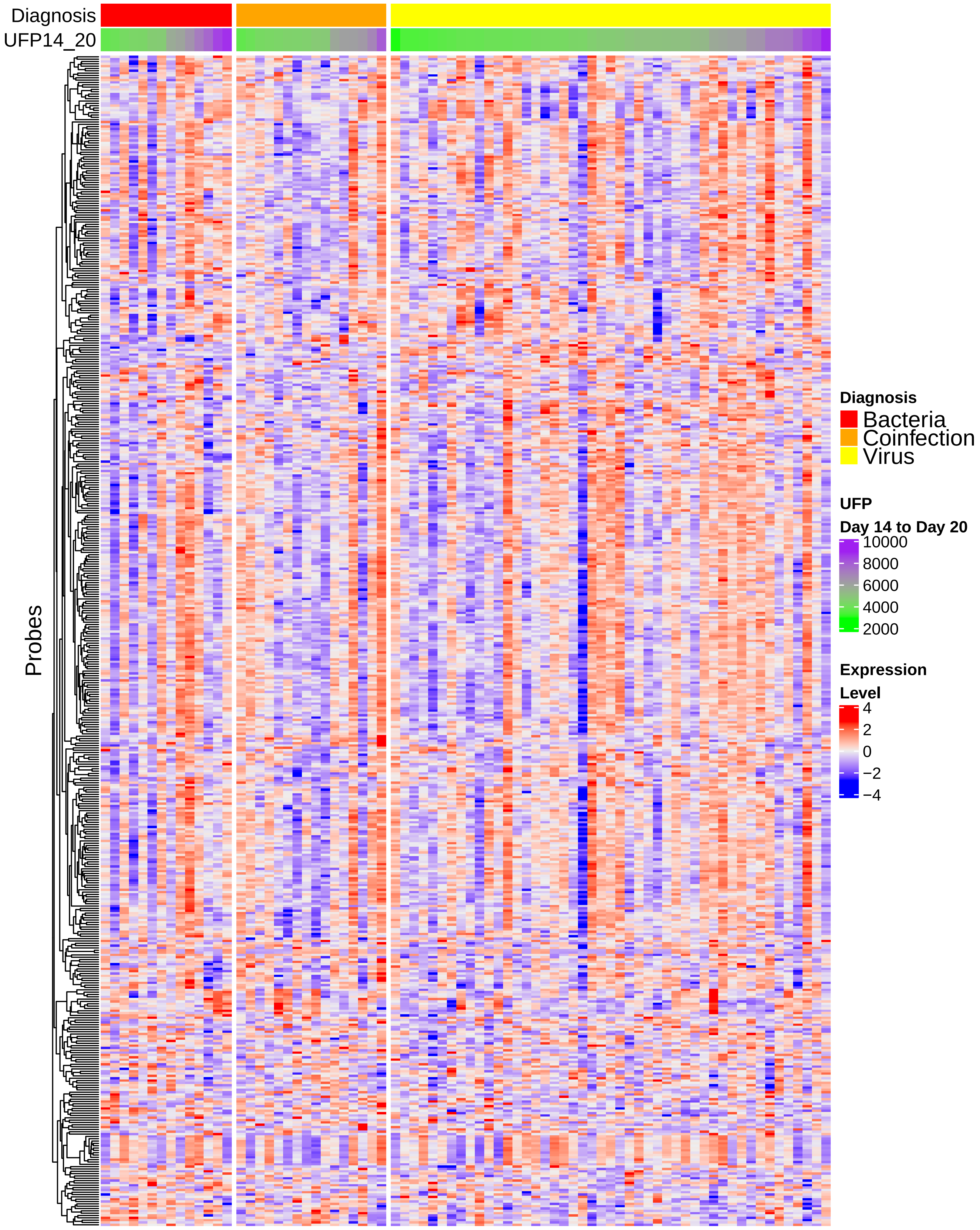

Figure S11: Gene expression heatmap of the Viral Gene Expression pathway sorted by diagnosis and concentration of Ultrafine particles (UFP) during lag days 21-27

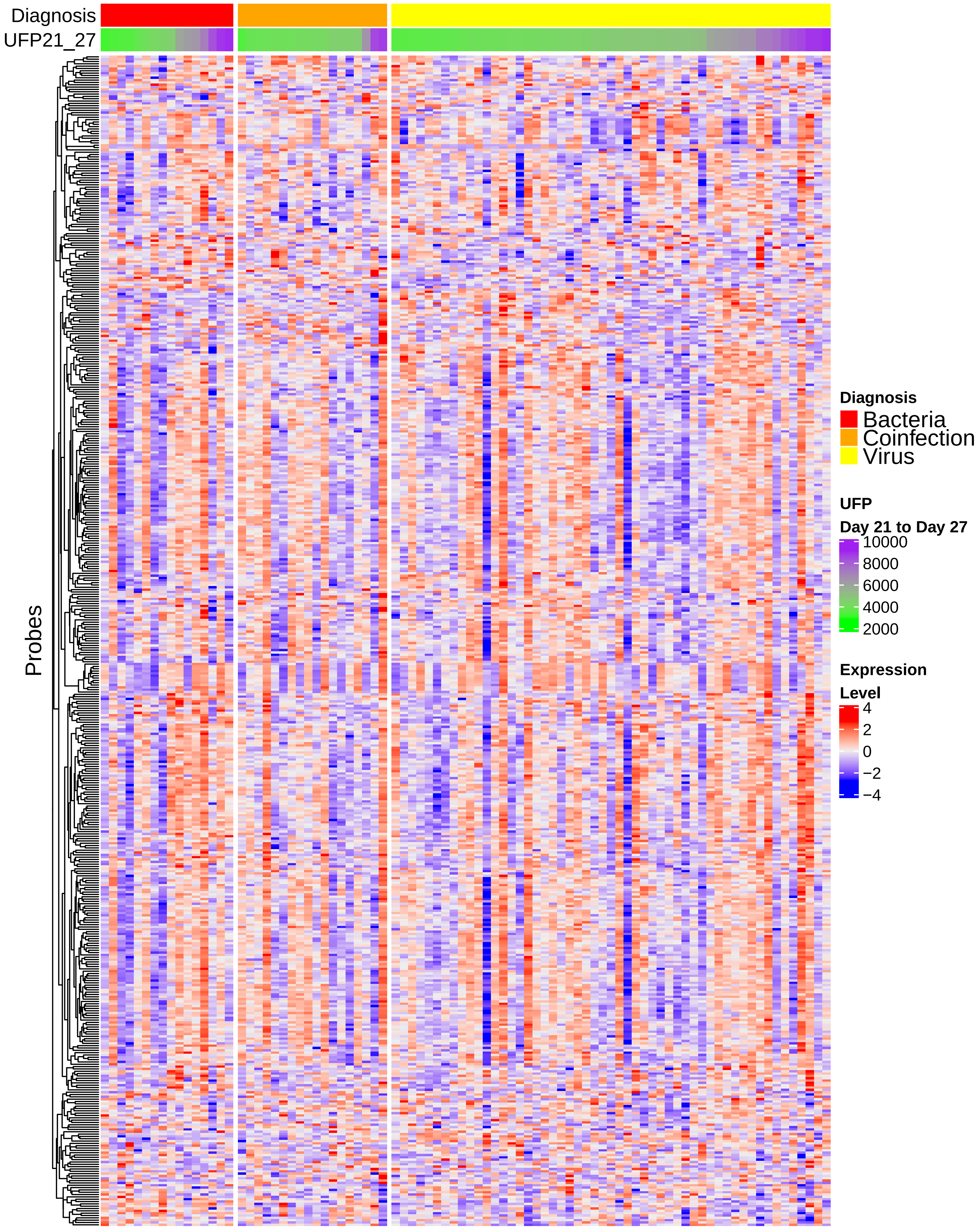

Figure S12: Gene expression heatmap of the Nuclear Transcribed mRNA Catabolic Process pathway sorted by diagnosis and concentration of accumulation mode particles (AMP) during lag days 0-6

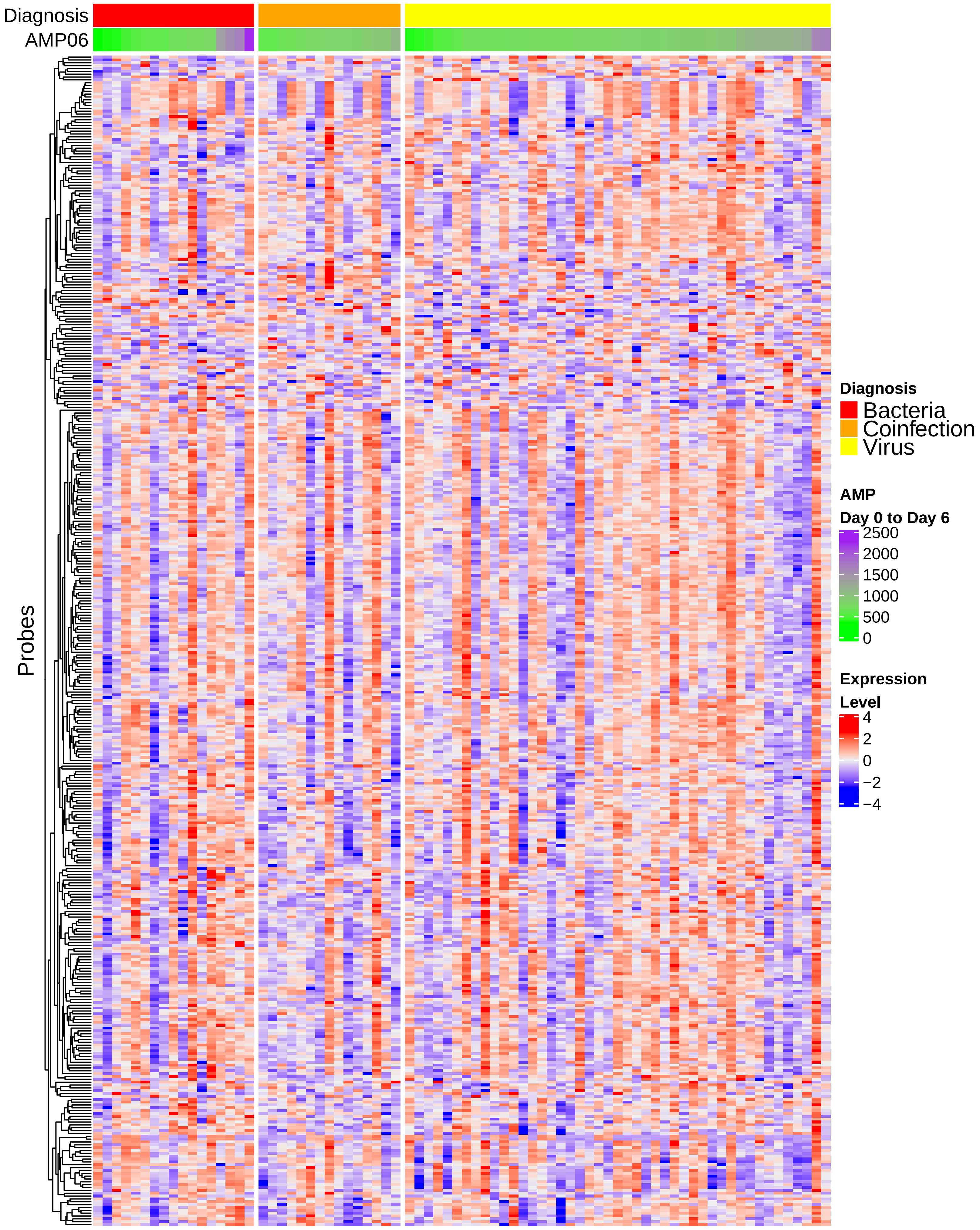

Supplement: Supplementary file 1 — Supplementary Information 1. [file 41598_2021_98729_MOESM1_ESM.pdf]
