# Supplementary material for: The effect of air pollution on the transcriptomics of the immune response to respiratory infection
Source: Sci Rep. 2021 Sep 30;11:19436. doi: 10.1038/s41598-021-98729-8 (PMC8484285; doi:10.1038/s41598-021-98729-8)
Supplement: Supplementary file 2 — Supplementary Information 2. [file 41598_2021_98729_MOESM2_ESM.docx]

**Supplemental File**

**The Effect of Air Pollution on the Transcriptomics of the**

**Immune Response to Respiratory Infection**

*Daniel P. Croft^1,2^, David S. Burton^3^, David J. Nagel^1,2^, Soumyaroop Bhattacharya^2,4^, Ann R. Falsey^5^, Steve N. Georas^1,2^, Philip K. Hopke^2,6,7^, Carl J. Johnston^2,4^, R. Matthew Kottmann^1,2^, Augusto A. Litonjua^1,2^, Thomas J. Mariani ^2,4,8^, David Q. Rich^1,2,4,6^, Kelly Thevenet-Morrison^6^, Sally W. Thurston^2,3^, Mark J. Utell^1,2^, Matthew N. McCall^2,3^

1. Department of Medicine, Pulmonary and Critical Care Medicine Division. University of Rochester Medical Center, Rochester, New York.
2. Environmental Health Science Center, University of Rochester Medical Center, Rochester, New York.
3. Department of Biostatistics and Computational Biology, University of Rochester Medical Center, Rochester, New York
4. Department of Pediatrics, University of Rochester Medical Center, Rochester, New York.
5. Department of Medicine, Infectious Diseases Division. University of Rochester Medical Center, Rochester, New York.
6. Department of Public Health Sciences, University of Rochester Medical Center, Rochester, New York
7. Institute for a Sustainable Environment, and Center for Air Resources Engineering and Science, Clarkson University, Potsdam, New York
8. Department of Biomedical Genetics. University of Rochester Medical Center, Rochester, New York.

***Corresponding author:**

Daniel P. Croft MD, MPH

University of Rochester Medical Center

Pulmonary and Critical Care Division

601 Elmwood Avenue Box 692

Rochester, NY 14642

Phone: 585 275 4161

Fax: 585 271 1171

Email: daniel_croft@urmc.rochester.edu

**Sensitivity Analysis of Smokers**

As a sensitivity analysis, we reran the individual gene analysis for Delta C at 0-6 hours stratified by smoking status. The differences observed when accounting for smoking were small. For example, the only change observed in smokers was that TUT4 (mRNA decay in oocytes) and not POC1B (centrosome proteins related to cilia formation) was significantly associated with increased concentrations of Delta-C and gene expression.(**Supplemental Table S10**)

**Sensitivity Analysis of Individual Genes within Significant Pathways**

We then performed an exploratory cross-reference analysis to understand which differentially expressed genes were present within the gene pathways associated with specific pollutants.

We observed multiple differentially expressed genes associated with increased air pollution concentrations, which were also present in the gene pathways associated with specific pollutants. For example, for increased concentrations of Delta-C at the 0-6 lag day, upregulation in the gene HMBS participates in multiple upregulated gene pathways pertaining to iron homeostasis. At later lag times, RPS4Y1 (codes for ribosomal subunit) was the most common gene upregulated across multiple pathways pertaining to ribosomal structure and function at multiple lag times. Finally, for increases in Delta-C concentrations in the 0-6 lag days, there were associations with multiple genes participating in the negative regulation of the viral life cycle were also observed (TRIM8, ISG15, OAS2) **(Supplemental Table S11).**

Though there were no individual genes associated with increases in BC, there were several gene pathways associated with increases in BC (**Table 3**). We then explored which differentially expressed genes were present in specific gene pathways **(Supplemental Table S12).** For example, for exposure to BC at 0-6 days, there were multiple differentially expressed genes present in both the specific pathway, Response To Type I Interferon and individual gene analysis, including IFI27, ISG15, OAS2, and OAS1. For BC at 0-28 days, several gene pathways related to protein folding (endoplasmic reticulum) shared a common gene, RPS4Y1. For PM_2.5_ at the 7-13 and 14-20 lag day, the RPS4Y1 gene was also observed within the ER specific pathways (**Supplemental Table S13**). For the later lag times of UFP (lag days 14-28), the RPS4Y1 gene was also present in the ER related pathways along with a variety of immune related genes (**Supplemental Table S14**). For all lag times of UFP, the gene STXBP2, which regulates cytotoxic granule exocytosis in natural killer cells, was the dominant gene present in immune pathways. For AMP, there were multiple genes across a variety of pathways (ER and immune) represented within the 0-6 and 21-28 day lag times. For AMP, iron homeostasis genes were also present at the 21-28 day lag period (**Supplemental Table S15**). Although there were common pathways associated with multiple different pollutants, each pollutant appeared to have unique patterns of association.

By comparing the differentially expressed genes from the individual gene analysis (LIMMA) with the significant gene pathways from the CAMERA analysis we could identify key genes that may be involved in the overall air pollution/respiratory infection association (Supplemental Tables S11-15). An iron homeostasis gene (HBMS) was the most frequently associated with increased concentrations of DC. Despite observing no individual genes associated with increases in BC concentrations, several genes related to the immune response to respiratory infection were identified as part of the individual gene/pathway cross-analysis. One particular gene, IFI27, appears to be differentially expressed in association with RVI, as well as in viral related pathways in association with BC exposure. What remains unclear is whether the expression of IFI27, or other genes, are part of the mechanistic pathway for the effect of air pollution on the immune response to RVI, or whether these genes are simply expressed in response to both RVI and air pollution. In this way, our analytic approach can help spotlight certain genes and pathways for further exploration in prospective exposure studies. In addition to focusing on specific immune related gene responses, future research will also need to focus on broader areas that could impair immune responses such as the aforementioned protein folding (ER) and iron homeostasis pathways.

**Table S1. Description of Respiratory Infections**

| **Infection Type** |  | **N(%)** |
| --- | --- | --- |
| **Viral infections** | |  |
| Influenza A | | 22(33.3) |
| Influenza B | | 5(7.6) |
| RSV (Respiratory Syncytial Virus Infection) | | 19(28.8) |
| Other viruses (Parainfluenza Virus (1-3), Human Metapneumovirus, H1N1 virus (swine flu), Rhinovirus, Coronavirus OC43) | | 20(30.3) |
| **Bacteria** | |  |
| Streptococcus pneumonia | | 12(60.0) |
| Other bacteria (Haemophilus influenzae, Moraxella catarrhalis, Mycoplasma pneumonia, Staphylococcus aureus) | | 8(40.0) |

**Table S2. Individual genes in Cluster 2 of the 150 most differentially expressed genes**

| Illumina Number | Gene name | Illumina Number | Gene name |
| --- | --- | --- | --- |
| ILMN_1733811 | JUP | ILMN_2239754 | IFIT3 |
| ILMN_2058782 | IFI27 | ILMN_1664543 | IFIT3 |
| ILMN_1658247 | OAS1 | ILMN_1674811 | OASL |
| ILMN_1663347 | OTOF | ILMN_1675640 | OAS1 |
| ILMN_1699331 | NA | ILMN_2410826 | OAS1 |
| ILMN_1783621 | CMPK2 | ILMN_1760062 | IFI44 |
| ILMN_1835092 | IFI44L | ILMN_1723912 | IFI44L |
| ILMN_1795181 | DDX60 | ILMN_1745397 | OAS3 |
| ILMN_1736729 | OAS2 | ILMN_2054019 | ISG15 |
| ILMN_2248970 | OAS2 | ILMN_1674063 | OAS2 |
| ILMN_2173975 | RTP4 | ILMN_1742618 | XAF1 |
| ILMN_1683678 | SPATS2L | ILMN_1657871 | RSAD2 |
| ILMN_1678422 | DHX58 | ILMN_1695404 | LY6E |
| ILMN_1718558 | PARP12 | ILMN_2388547 | EPSTI1 |
| ILMN_1654639 | HERC6 | ILMN_1662358 | MX1 |
| ILMN_2370573 | XAF1 | ILMN_1707695 | IFIT1 |
| ILMN_1701789 | IFIT3 | ILMN_1729749 | HERC5 |
| ILMN_1739428 | IFIT2 | ILMN_2347798 | IFI6 |

**Table S3 Association between pollution and gene expression.** Log fold change of gene expression in all patients with respiratory infection (all types combined) associated with a one unit increase of Delta Carbon concentrations at the 0-6 day lag period.

| **Illumina ID** | **Gene** | **Log Fold Change** | **Average**  **Expression** | ***Adjusted p-value** | **Beta** | **Standard Deviation** |
| --- | --- | --- | --- | --- | --- | --- |
| ILMN_1659024 | TMCC2 | 5.2 | 5.8 | 0.004 | 6.6 | 0.9 |
| ILMN_1714461 | RNF14 | 3.5 | 7.3 | 0.004 | 5.6 | 0.6 |
| ILMN_2164242 | UBE2F | 2.4 | 9.8 | 0.004 | 5.5 | 0.4 |
| ILMN_1747227 | ADORA1 | 2.9 | 5.9 | 0.004 | 5.1 | 0.6 |
| ILMN_2351241 | RNF14 | 3.2 | 6.6 | 0.004 | 5.1 | 0.6 |
| ILMN_1726308 | ST13P4 | 3.5 | 8.0 | 0.004 | 5.0 | 0.7 |
| ILMN_2174884 | XPO7 | 3.3 | 7.3 | 0.004 | 5.0 | 0.6 |
| ILMN_1694476 | HMBS | 5.2 | 6.5 | 0.005 | 4.5 | 1.0 |
| ILMN_1783156 | MAP2K3 | 3.1 | 10.3 | 0.006 | 4.2 | 0.6 |
| ILMN_1667201 | POC1B | 2.6 | 7.0 | 0.006 | 4.2 | 0.5 |
| *Adjusted p value uses the FDR metric to control for testing thousands of genes simultaneously | | | | | | |

**Table S4 Individual gene analysis** (Gene expression from infection alone-independent of air pollution).

| **Illumina ID** | **Gene** | **Log Fold change** | **Average Expression** | ***Adjusted**  **p- value** | **Beta** | **Standard Deviation** |
| --- | --- | --- | --- | --- | --- | --- |
| ILMN_2054019 | ISG15 | 3.3 | 10.5 | <0.001 | 14.4 | 1.6 |
| ILMN_1765332 | TIMM10 | 2.2 | 7.4 | <0.001 | 14.3 | 1.1 |
| ILMN_1660079 | RNF44 | -0.7 | 7.9 | <0.001 | 13.6 | 0.3 |
| ILMN_1723912 | IFI44L | 4.0 | 8.9 | <0.001 | 12.6 | 2.1 |
| ILMN_1760062 | IFI44 | 2.8 | 9.2 | <0.001 | 12.4 | 1.4 |
| ILMN_1674063 | OAS2 | 2.5 | 9.5 | <0.001 | 12.1 | 1.3 |
| ILMN_2058782 | IFI27 | 3.9 | 11.2 | <0.001 | 11.6 | 2.1 |
| ILMN_1679929 | KLF13 | -0.6 | 8.5 | <0.001 | 11.4 | 0.3 |
| ILMN_1703263 | SP140 | 1.2 | 5.6 | <0.001 | 11.4 | 0.7 |
| ILMN_1708991 | CCNY | -0.8 | 6.9 | <0.001 | 11.2 | 0.4 |
| *Adjusted p value uses the FDR metric to control for testing thousands of genes simultaneously | | | | | | |

**Supplemental Table S5 Timing and pattern of association between gene pathways and increased concentrations of Delta-C (red-upregulated, blue-downregulated).**

| **DC** | **Gene Pathway Name** |
| --- | --- |
| 0-6 | GO_SPLICEOSOMAL_COMPLEX |
| 21-27 | GO_NEGATIVE_REGULATION_OF_CALCIUM_ION_TRANSPORT_INTO_CYTOSOL |
| 21-27 | GO_NEGATIVE_REGULATION_OF_RYANODINE_SENSITIVE_CALCIUM_RELEASE_CHANNEL_ACTIVITY |
| 21-27 | GO_POSITIVE_REGULATION_OF_SEQUESTERING_OF_CALCIUM_ION |
| 0-6 | GO_HEME_METABOLIC_PROCESS |
| 0-6 | GO_HEMOGLOBIN_COMPLEX |
| 0-6 | GO_NEURAL_PLATE_ANTERIOR_POSTERIOR_REGIONALIZATION |
| 0-6 | GO_NEURAL_PLATE_PATTERN_SPECIFICATION |
| 0-6 | GO_NEURAL_PLATE_REGIONALIZATION |
| 0-6 | GO_PROTEIN_GLUTAMINE_GAMMA_GLUTAMYLTRANSFERASE_ACTIVITY |
| 7-13 | GO_HYDROGEN_PEROXIDE_CATABOLIC_PROCESS |
| 7-13 | GO_NASCENT_POLYPEPTIDE_ASSOCIATED_COMPLEX |
| 7-13 | GO_THIOREDOXIN_PEROXIDASE_ACTIVITY |
| 14-20 | GO_EUKARYOTIC_48S_PREINITIATION_COMPLEX |
| 14-20 | GO_EUKARYOTIC_TRANSLATION_INITIATION_FACTOR_3_COMPLEX |
| 14-20 | GO_EUKARYOTIC_TRANSLATION_INITIATION_FACTOR_3_COMPLEX_EIF3M |
| 14-20 | GO_FORMATION_OF_CYTOPLASMIC_TRANSLATION_INITIATION_COMPLEX |
| 14-20 | GO_TRANSLATION_ELONGATION_FACTOR_ACTIVITY |

**Supplemental Table S6. Timing and pattern of association between gene pathways and increased concentrations of Black carbon (red-upregulated, blue-downregulated).**

| **BC** | **Gene Pathway Name** |
| --- | --- |
| 7-13 | GO_MHC_CLASS_I_PROTEIN_COMPLEX_BINDING |
| 14-20 | GO_ANTIGEN_PROCESSING_AND_PRESENTATION_OF_EXOGENOUS_PEPTIDE_ANTIGEN_VIA_MHC_  CLASS_I_TAP_INDEPENDENT |
| 14-20 | GO_MHC_CLASS_I_PROTEIN_COMPLEX |
| 14-20 | GO_NATURAL_KILLER_CELL_CYTOKINE_PRODUCTION |
| 14-20 | GO_POSITIVE_REGULATION_OF_NATURAL_KILLER_CELL_MEDIATED_IMMUNITY |
| 0-6 | GO_DEFENSE_RESPONSE_TO_VIRUS |
| 0-6 | GO_INTERFERON_GAMMA_MEDIATED_SIGNALING_PATHWAY |
| 0-6 | GO_ISG15_PROTEIN_CONJUGATION |
| 0-6 | GO_NADPLUS_ADP_RIBOSYLTRANSFERASE_ACTIVITY |
| 0-6 | GO_NEGATIVE_REGULATION_OF_MULTI_ORGANISM_PROCESS |
| 0-6 | GO_NEGATIVE_REGULATION_OF_VIRAL_GENOME_REPLICATION |
| 0-6 | GO_NEGATIVE_REGULATION_OF_VIRAL_LIFE_CYCLE |
| 0-6 | GO_NEGATIVE_REGULATION_OF_VIRAL_PROCESS |
| 0-6 | GO_POSITIVE_REGULATION_OF_ENDOTHELIAL_CELL_CHEMOTAXIS |
| 0-6 | GO_POSITIVE_REGULATION_OF_TYPE_I_INTERFERON_MEDIATED_SIGNALING_PATHWAY |
| 0-6 | GO_PROTEIN_ADP_RIBOSYLASE_ACTIVITY |
| 0-6 | GO_PROTEIN_AUTO_ADP_RIBOSYLATION |
| 0-6 | GO_REGULATION_OF_RIBONUCLEASE_ACTIVITY |
| 0-6 | GO_REGULATION_OF_VIRAL_GENOME_REPLICATION |
| 0-6 | GO_REGULATION_OF_VIRAL_LIFE_CYCLE |
| 0-6 | GO_RESPONSE_TO_INTERFERON_ALPHA |
| 0-6 | GO_RESPONSE_TO_TYPE_I_INTERFERON |
| 0-6 | GO_RESPONSE_TO_VIRUS |
| 0-6 | GO_VIRAL_GENOME_REPLICATION |
| 7-13 | GO_CCR2_CHEMOKINE_RECEPTOR_BINDING |
| 7-13 | GO_EOSINOPHIL_ACTIVATION |
| 7-13 | GO_HEMATOPOIETIC_STEM_CELL_MIGRATION |
| 7-13 | GO_REGULATION_OF_LEUKOCYTE_TETHERING_OR_ROLLING |

**Supplemental Table S7. Timing and pattern of association between gene pathways and increased concentrations of PM_2.5_ (red-upregulated, blue-downregulated).**

| **PM25** | **Gene Pathway Name** |
| --- | --- |
| **0-6** | **GO_CYTOLYSIS_IN_OTHER_ORGANISM** |
| **0-6** | **GO_DISRUPTION_BY_HOST_OF_SYMBIONT_CELLS** |
| **0-6** | **GO_DISRUPTION_OF_CELLS_OF_OTHER_ORGANISM_INVOLVED_IN_SYMBIOTIC_INTERACTION** |
| **0-6** | **GO_NADPH_OXIDASE_COMPLEX** |
| **0-6** | **GO_PEROXISOME_TARGETING_SEQUENCE_BINDING** |
| **0-6** | **GO_PHAGOLYSOSOME** |
| **0-6** | **GO_SUPEROXIDE_GENERATING_NADPH_OXIDASE_ACTIVATOR_ACTIVITY** |
| **7-13** | **GO_MITOCHONDRIAL_PROTEIN_COMPLEX** |
| **7-13** | **GO_MITOCHONDRIAL_SMALL_RIBOSOMAL_SUBUNIT** |
| **7-13** | **GO_MITOCHONDRIAL_TRANSLATIONAL_TERMINATION** |
| **7-13** | **GO_ORGANELLAR_RIBOSOME** |
| **7-13** | **GO_RIBOSOME_ASSEMBLY** |
| **7-13** | **GO_RIBOSOME_BIOGENESIS** |
| **7-13** | **GO_ROUGH_ENDOPLASMIC_RETICULUM_MEMBRANE** |
| **7-13** | **GO_RRNA_METABOLIC_PROCESS** |
| **7-13** | **GO_TRANSLATIONAL_TERMINATION** |
| **14-20** | **GO_CYTOPLASMIC_SIDE_OF_ENDOPLASMIC_RETICULUM_MEMBRANE** |
| **14-20** | **GO_MITOCHONDRIAL_LARGE_RIBOSOMAL_SUBUNIT** |
| **14-20** | **GO_NCRNA_METABOLIC_PROCESS** |
| **14-20** | **GO_NCRNA_PROCESSING** |
| **14-20** | **GO_POSITIVE_REGULATION_OF_CELL_CELL_ADHESION_MEDIATED_BY_INTEGRIN** |
| **14-20** | **GO_PRERIBOSOME** |
| **21-27** | **GO_ADENYL_NUCLEOTIDE_EXCHANGE_FACTOR_ACTIVITY** |
| **21-27** | **GO_BETA_CATENIN_TCF_COMPLEX_ASSEMBLY** |
| **21-27** | **GO_POST_MRNA_RELEASE_SPLICEOSOMAL_COMPLEX** |
| **21-27** | **GO_RNA_POLYMERASE_II_GENERAL_TRANSCRIPTION_INITIATION_FACTOR_ACTIVITY** |
| **0-6** | **GO_CELLULAR_RESPONSE_TO_COPPER_ION** |
| **0-6** | **GO_DETOXIFICATION_OF_INORGANIC_COMPOUND** |
| **0-6** | **GO_MINERALOCORTICOID_SECRETION** |
| **0-6** | **GO_REGULATION_OF_MDA_5_SIGNALING_PATHWAY** |

**Supplemental Table S8 Timing and pattern of association between gene pathways and increased concentrations of ultrafine particles (red-upregulated, blue-downregulated).**

| **UFP** | **Gene Pathway Name** |
| --- | --- |
| 7-13 | GO_FICOLIN_1_RICH_GRANULE_MEMBRANE |
| 7-13 | GO_GLUCOSYLCERAMIDE_METABOLIC_PROCESS |
| 7-13 | GO_MYELOID_LEUKOCYTE_ACTIVATION |
| 7-13 | GO_PODOSOME |
| 7-13 | GO_REGULATION_OF_LEUKOCYTE_DEGRANULATION |
| 7-13 | GO_REGULATION_OF_NEUTROPHIL_ACTIVATION |
| 7-13 | GO_REGULATION_OF_SUPEROXIDE_ANION_GENERATION |
| 7-13 | GO_TERTIARY_GRANULE_MEMBRANE |
| 0-6 | GO_1_PHOSPHATIDYLINOSITOL_4_PHOSPHATE_5_KINASE_ACTIVITY |
| 0-6 | GO_2_IRON_2_SULFUR_CLUSTER_BINDING |
| 0-6 | GO_ANTERIOR_HEAD_DEVELOPMENT |
| 0-6 | GO_CELLULAR_MAGNESIUM_ION_HOMEOSTASIS |
| 0-6 | GO_REGULATION_BY_VIRUS_OF_VIRAL_PROTEIN_LEVELS_IN_HOST_CELL |
| 0-6 | GO_REGULATION_OF_MITOCHONDRIAL_ATP_SYNTHESIS_COUPLED_ELECTRON_TRANSPORT |
| 0-6 | GO_TETRAPYRROLE_BIOSYNTHETIC_PROCESS |
| 7-13 | GO_METAL_CLUSTER_BINDING |
| 14-20 | GO_RIBOSOMAL_LARGE_SUBUNIT_BIOGENESIS |
| 14-20 | GO_RRNA_BINDING |

**Supplemental Table S9 Timing and pattern of association between gene pathways and increased concentrations of Accumulation mode particles (red-upregulated, blue-downregulated).**

| **AMP** | **Gene Pathway Name** |
| --- | --- |
| 14-20 | GO_POSITIVE_REGULATION_OF_PHOSPHOLIPID_TRANSPORT |
| 21-27 | GO_AZUROPHIL_GRANULE_LUMEN |
| 21-27 | GO_RNA_METHYLATION |
| 21-27 | GO_VESICLE_LUMEN |
| 21-27 | GO_SEQUESTERING_OF_IRON_ION |
| 21-27 | GO_SEQUESTERING_OF_METAL_ION |

**Table S10 Pollution effect of DC 0-6 lag period, with Smoking Status**

Log fold change of gene expression in all patients with respiratory infection (all types combined) associated with a one unit increase of Delta Carbon concentrations at the 0-6 day lag period. These results control for smoking status of patient.

| **Illumina ID** | **Gene** | **Log Fold Change** | **Average**  **Expression** | ***Adjusted p-value** | **Beta** | **Standard**  **Deviation** |
| --- | --- | --- | --- | --- | --- | --- |
| ILMN_1659024 | TMCC2 | 5.2 | 5.8 | 0.007 | 6.1 | 0.9 |
| ILMN_1747227 | ADORA1 | 2.9 | 5.9 | 0.007 | 4.9 | 0.6 |
| ILMN_1714461 | RNF14 | 3.4 | 7.3 | 0.007 | 4.8 | 0.6 |
| ILMN_1726308 | ST13P4 | 3.6 | 8.0 | 0.007 | 4.8 | 0.7 |
| ILMN_2164242 | UBE2F | 2.3 | 9.8 | 0.007 | 4.7 | 0.4 |
| ILMN_1694476 | HMBS | 5.3 | 6.5 | 0.007 | 4.4 | 1.0 |
| ILMN_2174884 | XPO7 | 3.3 | 7.3 | 0.007 | 4.3 | 0.6 |
| ILMN_2351241 | RNF14 | 3.1 | 6.6 | 0.007 | 4.3 | 0.6 |
| ILMN_1655137 | TUT4 | -1.9 | 6.1 | 0.008 | 3.8 | 0.4 |
| ILMN_1783156 | MAP2K3 | 3.0 | 10.3 | 0.008 | 3.8 | 0.6 |
| *Adjusted p value uses the FDR metric to control for testing thousands of genes simultaneously | | | | | | |

**Table S11 Differentially expressed genes present in specific gene pathways associated with increased concentrations of Delta-C at multiple lag periods**

| **DC 0-6** |  |  |  |  |  |
| --- | --- | --- | --- | --- | --- |
| **Gene Symbols** | **Gene Pathway** | **NGenes** | **Direction** | **PValue** | **FDR** |
| HMBS | GO_TETRAPYRROLE_BIOSYNTHETIC_PROCESS | 37 | Up | <0.0001 | 0.0077 |
| HMBS | GO_PORPHYRIN_CONTAINING_COMPOUND_METABOLIC_PROCESS | 41 | Up | <0.0001 | 0.0018 |
| HMBS | GO_TETRAPYRROLE_METABOLIC_PROCESS | 59 | Up | 0.0018 | 0.0962 |
| HMBS | GO_HEME_BIOSYNTHETIC_PROCESS | 30 | Up | <0.0001 | 0.0067 |
| XPO7 | GO_RNA_LOCALIZATION | 328 | Down | 0.0002 | 0.0274 |
| FABP5 SERPING1 | GO_VESICLE_LUMEN | 397 | Down | 0.0011 | 0.0748 |
| DERL2 RPS4Y1 RPS4Y2 | GO_RIBONUCLEOPROTEIN_COMPLEX | 1147 | Down | 0.001 | 0.072 |
| TIMM10 | GO_MITOCHONDRIAL_PROTEIN_COMPLEX | 298 | Down | <0.0001 | 0.008 |
| EIF1AY RPS4Y1 RPS4Y2 OAS2 | GO_RNA_BINDING | 2299 | Down | 0.0013 | 0.0804 |
| HMBS | GO_PROTOPORPHYRINOGEN_IX_BIOSYNTHETIC_PROCESS | 13 | Up | <0.0001 | <0.0001 |
| HMBS | GO_PROTEIN_COFACTOR_LINKAGE | 15 | Up | 0.0009 | 0.0692 |
| XPO7 | GO_ESTABLISHMENT_OF_RNA_LOCALIZATION | 277 | Down | 0.0011 | 0.0741 |
| HMBS | GO_HEME_METABOLIC_PROCESS | 34 | Up | <0.0001 | 0.0046 |
| HMBS | GO_PROTOPORPHYRINOGEN_IX_METABOLIC_PROCESS | 17 | Up | <0.0001 | <0.0001 |
| TRIM8 ISG15 OAS2 | GO_NEGATIVE_REGULATION_OF_VIRAL_LIFE_CYCLE | 107 | Up | 0.001 | 0.0705 |
| **DC 7-13** |  |  |  |  |  |
| **Gene Symbols** | **Gene Pathway** | **NGenes** | **Direction** | **PValue** | **FDR** |
| EIF1AY RPS4Y1 | GO_TRANSLATIONAL_INITIATION | 497 | Up | <0.0001 | 0.0004 |
| RPS4Y1 | GO_ESTABLISHMENT_OF_PROTEIN_LOCALIZATION_TO_ENDOPLASMIC_RETICULUM | 396 | Up | <0.0001 | <0.0001 |
| RPS4Y1 | GO_PROTEIN_LOCALIZATION_TO_ENDOPLASMIC_RETICULUM | 423 | Up | <0.0001 | 0.0002 |
| RPS4Y1 | GO_PROTEIN_TARGETING_TO_MEMBRANE | 473 | Up | <0.0001 | 0.001 |
| RPS4Y1 | GO_NUCLEAR_TRANSCRIBED_MRNA_CATABOLIC_PROCESS_NONSENSE_MEDIATED_DECAY | 411 | Up | <0.0001 | <0.0001 |
| RPS4Y1 RPS4Y2 | GO_RIBOSOME | 536 | Up | <0.0001 | 0.001 |
| RPS4Y1 RPS4Y2 | GO_CYTOSOLIC_SMALL_RIBOSOMAL_SUBUNIT | 174 | Up | <0.0001 | 0.001 |
| RPS4Y1 RPS4Y2 | GO_SMALL_RIBOSOMAL_SUBUNIT | 210 | Up | <0.0001 | 0.0168 |
| STXBP2 | GO_SPECIFIC_GRANULE | 214 | Down | <0.0001 | 0.0123 |
| RPS4Y1 RPS4Y2 | GO_RIBOSOMAL_SUBUNIT | 490 | Up | <0.0001 | 0.0002 |
| RPS4Y1 RPS4Y2 | GO_CYTOSOLIC_RIBOSOME | 384 | Up | <0.0001 | <0.0001 |
| RPS4Y1 RPS4Y2 | GO_STRUCTURAL_CONSTITUENT_OF_RIBOSOME | 462 | Up | <0.0001 | 0.0002 |
| RPS4Y1 RPS4Y2 | GO_STRUCTURAL_MOLECULE_ACTIVITY | 791 | Up | 0.0003 | 0.0612 |
| RPS4Y1 | GO_NUCLEAR_TRANSCRIBED_MRNA_CATABOLIC_PROCESS | 513 | Up | <0.0001 | 0.0046 |
| RPS4Y1 | GO_COTRANSLATIONAL_PROTEIN_TARGETING_TO_MEMBRANE | 382 | Up | <0.0001 | <0.0001 |
| TRIM8 RPS4Y1 | GO_VIRAL_GENE_EXPRESSION | 503 | Up | <0.0001 | 0.0008 |
| **DC 14-20** |  |  |  |  |  |
| **Gene Symbols** | **Gene Pathway** | **NGenes** | **Direction** | **PValue** | **FDR** |
| EIF1AY RPS4Y1 | GO_TRANSLATIONAL_INITIATION | 497 | Up | <0.0001 | <0.0001 |
| OAS2 ISG15 OAS1 RSAD2 | GO_RESPONSE_TO_TYPE_I_INTERFERON | 125 | Down | 0.0006 | 0.0937 |
| RPS4Y1 | GO_ESTABLISHMENT_OF_PROTEIN_LOCALIZATION_TO_ENDOPLASMIC_RETICULUM | 396 | Up | <0.0001 | <0.0001 |
| RPS4Y1 | GO_PROTEIN_LOCALIZATION_TO_ENDOPLASMIC_RETICULUM | 423 | Up | <0.0001 | 0.0001 |
| RPS4Y1 | GO_PROTEIN_TARGETING_TO_MEMBRANE | 473 | Up | <0.0001 | 0.0024 |
| RPS4Y1 | GO_NUCLEAR_TRANSCRIBED_MRNA_CATABOLIC_PROCESS_NONSENSE_MEDIATED_DECAY | 411 | Up | <0.0001 | <0.0001 |
| RPS4Y1 RPS4Y2 | GO_RIBOSOME | 536 | Up | <0.0001 | 0.0004 |
| RPS4Y1 RPS4Y2 | GO_CYTOSOLIC_SMALL_RIBOSOMAL_SUBUNIT | 174 | Up | <0.0001 | 0.0011 |
| RPS4Y1 RPS4Y2 | GO_SMALL_RIBOSOMAL_SUBUNIT | 210 | Up | <0.0001 | 0.0212 |
| RPS4Y1 | GO_POLYSOME | 179 | Up | <0.0001 | 0.008 |
| RPS4Y1 RPS4Y2 | GO_RIBOSOMAL_SUBUNIT | 490 | Up | <0.0001 | 0.0004 |
| RPS4Y1 RPS4Y2 | GO_CYTOSOLIC_RIBOSOME | 384 | Up | <0.0001 | <0.0001 |
| RPS4Y1 RPS4Y2 | GO_STRUCTURAL_CONSTITUENT_OF_RIBOSOME | 462 | Up | <0.0001 | 0.0002 |
| EIF1AY | GO_TRANSLATION_FACTOR_ACTIVITY_RNA_BINDING | 128 | Up | 0.0001 | 0.0278 |
| RPS4Y1 RPS4Y2 | GO_RRNA_BINDING | 119 | Up | 0.0003 | 0.0662 |
| EIF1AY | GO_TRANSLATION_REGULATOR_ACTIVITY | 200 | Up | 0.0002 | 0.046 |
| EIF1AY | GO_TRANSLATION_REGULATOR_ACTIVITY_NUCLEIC_ACID_BINDING | 152 | Up | 0.0004 | 0.0695 |
| RPS4Y1 RPS4Y2 | GO_STRUCTURAL_MOLECULE_ACTIVITY | 791 | Up | 0.0003 | 0.0608 |
| RPS4Y1 | GO_NUCLEAR_TRANSCRIBED_MRNA_CATABOLIC_PROCESS | 513 | Up | <0.0001 | 0.0033 |
| RPS4Y1 | GO_COTRANSLATIONAL_PROTEIN_TARGETING_TO_MEMBRANE | 382 | Up | <0.0001 | <0.0001 |
| TRIM8 RPS4Y1 | GO_VIRAL_GENE_EXPRESSION | 503 | Up | <0.0001 | 0.0001 |
| **DC 21-27** |  |  |  |  |  |
| **Gene Symbols** | **Gene Pathway** | **NGenes** | **Direction** | **PValue** | **FDR** |
| EIF1AY RPS4Y1 | GO_TRANSLATIONAL_INITIATION | 497 | Up | <0.0001 | 0.002 |
| RPS4Y1 | GO_ESTABLISHMENT_OF_PROTEIN_LOCALIZATION_TO_ENDOPLASMIC_RETICULUM | 396 | Up | <0.0001 | 0.0027 |
| RPS4Y1 | GO_PROTEIN_LOCALIZATION_TO_ENDOPLASMIC_RETICULUM | 423 | Up | <0.0001 | 0.0111 |
| RPS4Y1 | GO_PROTEIN_TARGETING_TO_MEMBRANE | 473 | Up | 0.0001 | 0.0284 |
| RPS4Y1 | GO_NUCLEAR_TRANSCRIBED_MRNA_CATABOLIC_PROCESS_NONSENSE_MEDIATED_DECAY | 411 | Up | <0.0001 | 0.0009 |
| STXBP2 SERPING1 | GO_PLATELET_DEGRANULATION | 120 | Down | 0.0003 | 0.0542 |
| RPS4Y1 RPS4Y2 | GO_RIBOSOME | 536 | Up | 0.0001 | 0.0269 |
| RPS4Y1 RPS4Y2 | GO_CYTOSOLIC_SMALL_RIBOSOMAL_SUBUNIT | 174 | Up | <0.0001 | 0.0009 |
| RPS4Y1 RPS4Y2 | GO_SMALL_RIBOSOMAL_SUBUNIT | 210 | Up | <0.0001 | 0.019 |
| SERPING1 | GO_PLATELET_ALPHA_GRANULE_LUMEN | 59 | Down | 0.0007 | 0.0844 |
| STXBP2 | GO_SPECIFIC_GRANULE | 214 | Down | <0.0001 | 0.0087 |
| RPS4Y1 | GO_POLYSOME | 179 | Up | 0.0002 | 0.0302 |
| RPS4Y1 RPS4Y2 | GO_RIBOSOMAL_SUBUNIT | 490 | Up | <0.0001 | 0.0237 |
| RPS4Y1 RPS4Y2 | GO_CYTOSOLIC_RIBOSOME | 384 | Up | <0.0001 | 0.0005 |
| RPS4Y1 RPS4Y2 | GO_STRUCTURAL_CONSTITUENT_OF_RIBOSOME | 462 | Up | <0.0001 | 0.019 |
| EIF1AY | GO_TRANSLATION_FACTOR_ACTIVITY_RNA_BINDING | 128 | Up | 0.0003 | 0.0459 |
| EIF1AY | GO_TRANSLATION_REGULATOR_ACTIVITY | 200 | Up | 0.0009 | 0.0926 |
| EIF1AY | GO_TRANSLATION_REGULATOR_ACTIVITY_NUCLEIC_ACID_BINDING | 152 | Up | 0.0005 | 0.0718 |
| RPS4Y1 | GO_NUCLEAR_TRANSCRIBED_MRNA_CATABOLIC_PROCESS | 513 | Up | 0.0001 | 0.0273 |
| RPS4Y1 | GO_COTRANSLATIONAL_PROTEIN_TARGETING_TO_MEMBRANE | 382 | Up | <0.0001 | 0.0009 |
| TRIM8 RPS4Y1 | GO_VIRAL_GENE_EXPRESSION | 503 | Up | <0.0001 | 0.015 |
| STXBP2 | GO_CYTOLYTIC_GRANULE | 7 | Down | 0.0002 | 0.0425 |

**Table S12 Differentially expressed genes present in specific gene pathways associated with increased concentrations of black carbon at multiple lag periods**

| **BC 0-6** |  |  |  |  |  |
| --- | --- | --- | --- | --- | --- |
| **GeneSymbols** | **Gene Pathway** | **NGenes** | **Direction** | **PValue** | **FDR** |
| TRIM8 ISG15 OAS2 RSAD2 OAS1 | GO_NEGATIVE_REGULATION_OF_VIRAL_PROCESS | 124 | Up | <0.0001 | <0.0001 |
| IFI27 ISG15 OAS2 RSAD2 OAS1 | GO_RESPONSE_TO_TYPE_I_INTERFERON | 125 | Up | <0.0001 | <0.0001 |
| IFI27 ISG15 OAS2 IFI44L RSAD2 OAS1 | GO_DEFENSE_RESPONSE_TO_VIRUS | 296 | Up | <0.0001 | <0.0001 |
| IFI27 TRIM8 ISG15 OAS2 RSAD2 OAS1 | GO_REGULATION_OF_SYMBIOSIS_ENCOMPASSING_MUTUALISM_THROUGH_PARASITISM | 282 | Up | 0.0005 | 0.0975 |
| IFI27 ISG15 OAS2 RSAD2 OAS1 | GO_VIRAL_GENOME_REPLICATION | 184 | Up | <0.0001 | 0.0029 |
| TRIM8 OAS2 OAS1 | GO_INTERFERON_GAMMA_MEDIATED_SIGNALING_PATHWAY | 111 | Up | <0.0001 | 0.0002 |
| TRIM8 OAS2 OAS1 | GO_RESPONSE_TO_INTERFERON_GAMMA | 214 | Up | 0.0004 | 0.0884 |
| SERPING1 TRIM8 ISG15 OAS2 RSAD2 OAS1 | GO_NEGATIVE_REGULATION_OF_MULTI_ORGANISM_PROCESS | 260 | Up | <0.0001 | 0.0015 |
| IFI27 ISG15 OAS2 RSAD2 OAS1 | GO_REGULATION_OF_VIRAL_GENOME_REPLICATION | 139 | Up | <0.0001 | <0.0001 |
| ISG15 OAS2 RSAD2 OAS1 | GO_NEGATIVE_REGULATION_OF_VIRAL_GENOME_REPLICATION | 81 | Up | <0.0001 | <0.0001 |
| TRIM8 | GO_REGULATION_OF_VIRAL_ENTRY_INTO_HOST_CELL | 33 | Up | <0.0001 | 0.012 |
| IFI27 ISG15 OAS2 IFI44 IFI44L RSAD2 OAS1 | GO_RESPONSE_TO_VIRUS | 398 | Up | <0.0001 | 0.0002 |
| OAS2 OAS1 | GO_ADENYLYLTRANSFERASE_ACTIVITY | 32 | Up | <0.0001 | 0.0073 |
| OAS2 OAS1 | GO_DOUBLE_STRANDED_RNA_BINDING | 101 | Up | 0.0002 | 0.0451 |
| ISG15 | GO_ISG15_PROTEIN_CONJUGATION | 8 | Up | <0.0001 | 0.0012 |
| RSAD2 | GO_TOLL_LIKE_RECEPTOR_7_SIGNALING_PATHWAY | 15 | Up | 0.0001 | 0.0397 |
| OAS2 OAS1 | GO_REGULATION_OF_RIBONUCLEASE_ACTIVITY | 22 | Up | <0.0001 | <0.0001 |
| IFI27 TRIM8 ISG15 OAS2 RSAD2 OAS1 | GO_REGULATION_OF_VIRAL_LIFE_CYCLE | 192 | Up | <0.0001 | <0.0001 |
| CARD17 | GO_CASPASE_BINDING | 10 | Up | <0.0001 | 0.0262 |
| TRIM8 ISG15 OAS2 RSAD2 OAS1 | GO_NEGATIVE_REGULATION_OF_VIRAL_LIFE_CYCLE | 107 | Up | <0.0001 | <0.0001 |
| **BC 21-27** |  |  |  |  |  |
| **GeneSymbols** | **Gene Pathway** | **NGenes** | **Direction** | **PValue** | **FDR** |
| EIF1AY RPS4Y1 | GO_TRANSLATIONAL_INITIATION | 497 | Up | <0.0001 | <0.0001 |
| RPS4Y1 TIMM10 | GO_PROTEIN_TARGETING | 739 | Up | 0.0003 | 0.0627 |
| RPS4Y1 | GO_ESTABLISHMENT_OF_PROTEIN_LOCALIZATION_TO_ENDOPLASMIC_RETICULUM | 396 | Up | <0.0001 | <0.0001 |
| RPS4Y1 | GO_PROTEIN_LOCALIZATION_TO_ENDOPLASMIC_RETICULUM | 423 | Up | <0.0001 | <0.0001 |
| RPS4Y1 | GO_PROTEIN_TARGETING_TO_MEMBRANE | 473 | Up | <0.0001 | <0.0001 |
| RPS4Y1 TIMM10 | GO_ESTABLISHMENT_OF_PROTEIN_LOCALIZATION_TO_MEMBRANE | 626 | Up | <0.0001 | 0.0332 |
| RPS4Y1 | GO_NUCLEAR_TRANSCRIBED_MRNA_CATABOLIC_PROCESS_NONSENSE_MEDIATED_DECAY | 411 | Up | <0.0001 | <0.0001 |
| ISG15 OAS2 OAS1 | GO_NEGATIVE_REGULATION_OF_VIRAL_GENOME_REPLICATION | 81 | Up | 0.0003 | 0.0635 |
| RPS4Y1 RPS4Y2 | GO_RIBOSOME | 536 | Up | <0.0001 | <0.0001 |
| RPS4Y1 RPS4Y2 | GO_CYTOSOLIC_SMALL_RIBOSOMAL_SUBUNIT | 174 | Up | <0.0001 | <0.0001 |
| RPS4Y1 RPS4Y2 | GO_SMALL_RIBOSOMAL_SUBUNIT | 210 | Up | <0.0001 | <0.0001 |
| STXBP2 | GO_SPECIFIC_GRANULE | 214 | Down | 0.0001 | 0.0378 |
| RPS4Y1 | GO_POLYSOME | 179 | Up | <0.0001 | 0.0052 |
| RPS4Y1 RPS4Y2 | GO_RIBOSOMAL_SUBUNIT | 490 | Up | <0.0001 | <0.0001 |
| RPS4Y1 RPS4Y2 | GO_CYTOSOLIC_RIBOSOME | 384 | Up | <0.0001 | <0.0001 |
| RPS4Y1 RPS4Y2 | GO_STRUCTURAL_CONSTITUENT_OF_RIBOSOME | 462 | Up | <0.0001 | <0.0001 |
| RPS4Y1 | GO_NUCLEAR_TRANSCRIBED_MRNA_CATABOLIC_PROCESS | 513 | Up | <0.0001 | <0.0001 |
| RPS4Y1 | GO_COTRANSLATIONAL_PROTEIN_TARGETING_TO_MEMBRANE | 382 | Up | <0.0001 | <0.0001 |
| TRIM8 RPS4Y1 | GO_VIRAL_GENE_EXPRESSION | 503 | Up | <0.0001 | <0.0001 |
| OAS2 OAS1 | GO_REGULATION_OF_RIBONUCLEASE_ACTIVITY | 22 | Up | 0.0001 | 0.0403 |
| STXBP2 | GO_CYTOLYTIC_GRANULE | 7 | Down | 0.0002 | 0.0501 |

**Table S13. Differentially expressed genes present in specific gene pathways associated with increased concentrations of PM_2.5_ at multiple lag periods**

| **PM25 0-6** |  |  |  |  |  |
| --- | --- | --- | --- | --- | --- |
| **GeneSymbols** | **Differentially Expressed Gene** | **NGenes** | **Direction** | **PValue** | **FDR** |
| ORM1 STXBP2 | GO_SPECIFIC_GRANULE | 214 | Down | <0.0001 | 0.0808 |
| ORM1 | GO_SPECIFIC_GRANULE_LUMEN | 73 | Down | <0.0001 | 0.0102 |
| **PM25 7-13** |  |  |  |  |  |
| **GeneSymbols** | **Differentially Expressed Gene** | **NGenes** | **Direction** | **PValue** | **FDR** |
| ISG15 | GO_MYELOID_CELL_HOMEOSTASIS | 204 | Up | 0.0019 | 0.0955 |
| RPS4Y1 EIF1AY | GO_TRANSLATIONAL_INITIATION | 497 | Down | 0.0001 | 0.0199 |
| MRPS34 | GO_MITOCHONDRIAL_TRANSLATION | 154 | Down | <0.0001 | 0.0001 |
| RPS4Y1 | GO_ESTABLISHMENT_OF_PROTEIN_LOCALIZATION_TO_ENDOPLASMIC_RETICULUM | 396 | Down | <0.0001 | 0.0014 |
| TSPO | GO_TETRAPYRROLE_BIOSYNTHETIC_PROCESS | 37 | Up | 0.0006 | 0.0528 |
| MRPS34 | GO_TRANSLATIONAL_TERMINATION | 129 | Down | <0.0001 | 0.0005 |
| TSPO | GO_PORPHYRIN_CONTAINING_COMPOUND_METABOLIC_PROCESS | 41 | Up | <0.0001 | 0.0076 |
| RPS4Y1 | GO_PROTEIN_LOCALIZATION_TO_ENDOPLASMIC_RETICULUM | 423 | Down | <0.0001 | 0.0039 |
| RPS4Y1 | GO_PROTEIN_TARGETING_TO_MEMBRANE | 473 | Down | 0.0002 | 0.024 |
| RPS4Y1 TIMM10 | GO_ESTABLISHMENT_OF_PROTEIN_LOCALIZATION_TO_MEMBRANE | 626 | Down | 0.0016 | 0.0879 |
| MRPS34 | GO_TRANSLATIONAL_ELONGATION | 187 | Down | <0.0001 | 0.0057 |
| RPS4Y1 | GO_NUCLEAR_TRANSCRIBED_MRNA_CATABOLIC_PROCESS_NONSENSE_MEDIATED_DECAY | 411 | Down | <0.0001 | 0.0086 |
| TSPO | GO_HEME_BIOSYNTHETIC_PROCESS | 30 | Up | <0.0001 | 0.0122 |
| RPS4Y1 OAS2 | GO_RNA_CATABOLIC_PROCESS | 768 | Down | 0.0016 | 0.0879 |
| MRPS34 RPS4Y1 RPS4Y2 | GO_RIBOSOME | 536 | Down | <0.0001 | 0.0028 |
| USPL1 | GO_CAJAL_BODY | 67 | Down | 0.0004 | 0.0362 |
| RPS4Y1 RPS4Y2 | GO_CYTOSOLIC_SMALL_RIBOSOMAL_SUBUNIT | 174 | Down | <0.0001 | 0.0076 |
| MRPS34 RPS4Y1 RPS4Y2 | GO_SMALL_RIBOSOMAL_SUBUNIT | 210 | Down | <0.0001 | 0.0012 |
| MRPS34 | GO_ORGANELLAR_RIBOSOME | 103 | Down | <0.0001 | 0.0001 |
| TIMM10 | GO_INNER_MITOCHONDRIAL_MEMBRANE_PROTEIN_COMPLEX | 143 | Down | <0.0001 | 0.0144 |
| RPS4Y1 | GO_POLYSOME | 179 | Down | 0.0009 | 0.0629 |
| MRPS34 RPS4Y1 RPS4Y2 | GO_RIBOSOMAL_SUBUNIT | 490 | Down | <0.0001 | 0.0009 |
| MRPS34 RPS4Y1 RPS4Y2 | GO_RIBONUCLEOPROTEIN_COMPLEX | 1147 | Down | 0.0001 | 0.0181 |
| MRPS34 TIMM10 | GO_MITOCHONDRIAL_PROTEIN_COMPLEX | 298 | Down | <0.0001 | 0.0006 |
| RPS4Y1 RPS4Y2 | GO_CYTOSOLIC_RIBOSOME | 384 | Down | <0.0001 | 0.0076 |
| MRPS34 RPS4Y1 RPS4Y2 | GO_STRUCTURAL_CONSTITUENT_OF_RIBOSOME | 462 | Down | <0.0001 | 0.0006 |
| RPS4Y1 RPS4Y2 | GO_RRNA_BINDING | 119 | Down | 0.0002 | 0.024 |
| RPS4Y1 | GO_NUCLEAR_TRANSCRIBED_MRNA_CATABOLIC_PROCESS | 513 | Down | <0.0001 | 0.0086 |
| RPS4Y1 | GO_COTRANSLATIONAL_PROTEIN_TARGETING_TO_MEMBRANE | 382 | Down | <0.0001 | 0.0026 |
| TRIM8 RPS4Y1 | GO_VIRAL_GENE_EXPRESSION | 503 | Down | 0.0004 | 0.0419 |
| MRPS34 RPS4Y1 EIF1AY RPS4Y2 | GO_PEPTIDE_BIOSYNTHETIC_PROCESS | 1052 | Down | 0.0012 | 0.0735 |
| MRPS34 | GO_MITOCHONDRIAL_TRANSLATIONAL_TERMINATION | 109 | Down | <0.0001 | 0.0001 |
| MRPS34 | GO_MITOCHONDRIAL_GENE_EXPRESSION | 190 | Down | <0.0001 | 0.0006 |
| TSPO | GO_HEME_METABOLIC_PROCESS | 34 | Up | <0.0001 | 0.0118 |
| TSPO | GO_NEGATIVE_REGULATION_OF_ATP_METABOLIC_PROCESS | 27 | Up | 0.0017 | 0.0898 |
| MRPS34 | GO_MITOCHONDRIAL_SMALL_RIBOSOMAL_SUBUNIT | 33 | Down | <0.0001 | 0.0016 |
| **PM25 14-20** |  |  |  |  |  |
| **GeneSymbols** | **Gene Pathway** | **NGenes** | **Direction** | **PValue** | **FDR** |
| RPS4Y1 RPS4Y2 | GO_RIBOSOME | 536 | Down | 0.0012 | 0.0923 |
| RPS4Y1 RPS4Y2 | GO_CYTOSOLIC_SMALL_RIBOSOMAL_SUBUNIT | 174 | Down | 0.0006 | 0.0622 |
| RPS4Y1 RPS4Y2 | GO_SMALL_RIBOSOMAL_SUBUNIT | 210 | Down | <0.0001 | 0.0186 |
| RPS4Y1 RPS4Y2 | GO_RIBOSOMAL_SUBUNIT | 490 | Down | 0.0004 | 0.0523 |
| RPS4Y1 RPS4Y2 | GO_RIBONUCLEOPROTEIN_COMPLEX | 1147 | Down | <0.0001 | 0.0196 |
| RPS4Y1 RPS4Y2 | GO_STRUCTURAL_CONSTITUENT_OF_RIBOSOME | 462 | Down | 0.0007 | 0.0625 |
| EIF1AY RPS4Y1 RPS4Y2 | GO_RNA_BINDING | 2299 | Down | 0.0004 | 0.0512 |

**Table S14. Differentially expressed genes present in specific gene pathways associated with increased concentrations of ultrafine particles at multiple lag periods**

| **UFP 0-6** |  |  |  |  |  |
| --- | --- | --- | --- | --- | --- |
| **GeneSymbols** | **Gene Pathway** | **NGenes** | **Direction** | **PValue** | **FDR** |
| STXBP2 | GO_MYELOID_LEUKOCYTE_MEDIATED_IMMUNITY | 760 | Down | 0.0003 | 0.0577 |
| STXBP2 | GO_REGULATION_OF_LEUKOCYTE_DEGRANULATION | 65 | Down | 0.0005 | 0.0858 |
| STXBP2 | GO_SPECIFIC_GRANULE | 214 | Down | <0.0001 | 0.0022 |
| STXBP2 | GO_AZUROPHIL_GRANULE | 209 | Down | 0.0005 | 0.0858 |
| STXBP2 | GO_TERTIARY_GRANULE | 224 | Down | 0.0003 | 0.0653 |
| **UFP 7-13** |  |  |  |  |  |
| **GeneSymbols** | **Gene Pathway** | **NGenes** | **Direction** | **PValue** | **FDR** |
| STXBP2 RARA | GO_CELL_ACTIVATION_INVOLVED_IN_IMMUNE_RESPONSE | 928 | Down | 0.0002 | 0.0573 |
| STXBP2 | GO_MYELOID_LEUKOCYTE_ACTIVATION | 867 | Down | <0.0001 | 0.0422 |
| STXBP2 | GO_MYELOID_LEUKOCYTE_MEDIATED_IMMUNITY | 760 | Down | <0.0001 | 0.013 |
| STXBP2 | GO_REGULATION_OF_LEUKOCYTE_DEGRANULATION | 65 | Down | <0.0001 | 0.0449 |
| STXBP2 LHFPL2 | GO_EXOCYTOSIS | 1042 | Down | 0.0001 | 0.0512 |
| STXBP2 LHFPL2 | GO_SECRETORY_GRANULE | 918 | Down | 0.0003 | 0.0641 |
| STXBP2 | GO_SPECIFIC_GRANULE | 214 | Down | <0.0001 | <0.0001 |
| STXBP2 LHFPL2 | GO_SECRETORY_GRANULE_MEMBRANE | 361 | Down | <0.0001 | 0.0049 |
| RPS4Y1 RPS4Y2 | GO_CYTOSOLIC_RIBOSOME | 384 | Up | 0.0001 | 0.0512 |
| MAPK3 | GO_REGULATION_OF_CYCLASE_ACTIVITY | 30 | Down | 0.0002 | 0.052 |
| STXBP2 | GO_AZUROPHIL_GRANULE | 209 | Down | 0.0002 | 0.0512 |
| STXBP2 | GO_TERTIARY_GRANULE | 224 | Down | <0.0001 | 0.0024 |
| **UFP 14-20** |  |  |  |  |  |
| GeneSymbols | Gene Pathway | NGenes | Direction | PValue | FDR |
| SHKBP1 | GO_REGULATION_OF_ERBB_SIGNALING_PATHWAY | 116 | Down | 0.0055 | 0.0971 |
| SCARB2 | GO_ENDOSOME_ORGANIZATION | 77 | Down | 0.0004 | 0.0187 |
| SCARB2 | GO_VESICLE_ORGANIZATION | 359 | Down | 0.0003 | 0.0143 |
| STXBP2 | GO_REGULATION_OF_MAST_CELL_ACTIVATION_INVOLVED_IN_IMMUNE_RESPONSE | 34 | Down | 0.0005 | 0.0211 |
| EIF1AY RPS4Y1 | GO_TRANSLATIONAL_INITIATION | 497 | Up | <0.0001 | <0.0001 |
| SCARB2 RPS4Y1 TIMM10 | GO_PROTEIN_TARGETING | 739 | Up | <0.0001 | <0.0001 |
| STXBP2 | GO_REGULATION_OF_EXOCYTOSIS | 190 | Down | <0.0001 | 0.0002 |
| OAS2 | GO_REGULATION_OF_BODY_FLUID_LEVELS | 452 | Down | 0.0002 | 0.0113 |
| RPS4Y1 | GO_ESTABLISHMENT_OF_PROTEIN_LOCALIZATION_TO_ENDOPLASMIC_RETICULUM | 396 | Up | <0.0001 | <0.0001 |
| ORM1 STXBP2 | GO_CELL_ACTIVATION | 1616 | Down | 0.0007 | 0.0282 |
| SCARB2 | GO_REGULATION_OF_VACUOLE_ORGANIZATION | 61 | Down | <0.0001 | 0.006 |
| SCARB2 RPS4Y1 TIMM10 | GO_ESTABLISHMENT_OF_PROTEIN_LOCALIZATION_TO_ORGANELLE | 904 | Up | <0.0001 | 0.0011 |
| ORM1 | GO_INTERLEUKIN_1_PRODUCTION | 127 | Down | 0.0003 | 0.0146 |
| ORM1 BTN3A3 STXBP2 | GO_LEUKOCYTE_MEDIATED_IMMUNITY | 988 | Down | <0.0001 | 0.0035 |
| STXBP2 | GO_REGULATION_OF_VESICLE_MEDIATED_TRANSPORT | 511 | Down | 0.0001 | 0.0083 |
| ORM1 | GO_NEGATIVE_REGULATION_OF_INTERLEUKIN_6_PRODUCTION | 49 | Down | 0.0007 | 0.0268 |
| RPS4Y1 | GO_PROTEIN_LOCALIZATION_TO_ENDOPLASMIC_RETICULUM | 423 | Up | <0.0001 | <0.0001 |
| EIF1AY RPS4Y1 RPS4Y2 | GO_PEPTIDE_METABOLIC_PROCESS | 1190 | Up | <0.0001 | <0.0001 |
| ORM1 OAS2 STXBP2 ISG15 | GO_SECRETION | 1645 | Down | 0.0003 | 0.0138 |
| ORM1 STXBP2 | GO_CELL_ACTIVATION_INVOLVED_IN_IMMUNE_RESPONSE | 928 | Down | <0.0001 | 0.0028 |
| SCARB2 | GO_CELLULAR_CARBOHYDRATE_CATABOLIC_PROCESS | 50 | Down | 0.0004 | 0.0179 |
| STXBP2 | GO_MAST_CELL_ACTIVATION | 63 | Down | 0.0003 | 0.0152 |
| RPS4Y1 | GO_PROTEIN_TARGETING_TO_MEMBRANE | 473 | Up | <0.0001 | <0.0001 |
| RPS4Y1 TIMM10 | GO_PROTEIN_LOCALIZATION_TO_MEMBRANE | 896 | Up | 0.0004 | 0.0175 |
| OAS2 ISG15 IFI44 | GO_RESPONSE_TO_BACTERIUM | 543 | Down | 0.0017 | 0.0504 |
| ORM1 OAS2 STXBP2 | GO_REGULATION_OF_SECRETION | 701 | Down | 0.0051 | 0.0941 |
| ORM1 | GO_ACUTE_INFLAMMATORY_RESPONSE | 88 | Down | <0.0001 | 0.0019 |
| ORM1 STXBP2 | GO_MYELOID_LEUKOCYTE_ACTIVATION | 867 | Down | <0.0001 | 0.0002 |
| TIMM10 | GO_INNER_MITOCHONDRIAL_MEMBRANE_ORGANIZATION | 58 | Up | 0.0002 | 0.0102 |
| EIF1AY RPS4Y1 RPS4Y2 | GO_CELLULAR_AMIDE_METABOLIC_PROCESS | 1444 | Up | <0.0001 | 0.0046 |
| STXBP2 | GO_LYSOSOME_LOCALIZATION | 87 | Down | <0.0001 | 0.0019 |
| STXBP2 | GO_REGULATION_OF_MAST_CELL_ACTIVATION | 41 | Down | <0.0001 | 0.0063 |
| EIF1AY RPS4Y1 RPS4Y2 | GO_AMIDE_BIOSYNTHETIC_PROCESS | 1194 | Up | <0.0001 | <0.0001 |
| ORM1 | GO_POSITIVE_REGULATION_OF_INTERLEUKIN_1_PRODUCTION | 69 | Down | 0.0024 | 0.0597 |
| RPS4Y1 TIMM10 | GO_ESTABLISHMENT_OF_PROTEIN_LOCALIZATION_TO_MEMBRANE | 626 | Up | <0.0001 | <0.0001 |
| ORM1 | GO_INFLAMMATORY_RESPONSE | 698 | Down | 0.0005 | 0.0214 |
| STXBP2 | GO_REGULATION_OF_REGULATED_SECRETORY_PATHWAY | 137 | Down | <0.0001 | 0.0007 |
| ORM1 | GO_POSITIVE_REGULATION_OF_CYTOKINE_SECRETION | 151 | Down | 0.0024 | 0.0604 |
| ORM1 STXBP2 | GO_MYELOID_LEUKOCYTE_MEDIATED_IMMUNITY | 760 | Down | <0.0001 | <0.0001 |
| ORM1 ISG15 | GO_CYTOKINE_SECRETION | 250 | Down | 0.0011 | 0.0374 |
| OAS2 RPS4Y1 | GO_ORGANIC_CYCLIC_COMPOUND_CATABOLIC_PROCESS | 968 | Up | <0.0001 | 0.0005 |
| TRIM8 OAS2 SCARB2 RPS4Y1 ISG15 | GO_INTERSPECIES_INTERACTION_BETWEEN_ORGANISMS | 1432 | Up | 0.0018 | 0.0517 |
| TRIM8 SCARB2 RPS4Y1 TIMM10 | GO_PROTEIN_LOCALIZATION_TO_ORGANELLE | 1306 | Up | 0.003 | 0.0685 |
| STXBP2 | GO_MAST_CELL_MEDIATED_IMMUNITY | 55 | Down | 0.0008 | 0.0295 |
| SCARB2 | GO_LYTIC_VACUOLE_ORGANIZATION | 69 | Down | 0.0053 | 0.0947 |
| RPS4Y1 | GO_MRNA_METABOLIC_PROCESS | 1313 | Up | 0.0008 | 0.029 |
| EIF1AY RPS4Y1 RPS4Y2 | GO_ORGANONITROGEN_COMPOUND_BIOSYNTHETIC_PROCESS | 2133 | Up | 0.001 | 0.0359 |
| RPS4Y1 | GO_NUCLEAR_TRANSCRIBED_MRNA_CATABOLIC_PROCESS_NONSENSE_MEDIATED_DECAY | 411 | Up | <0.0001 | <0.0001 |
| STXBP2 | GO_REGULATION_OF_LEUKOCYTE_DEGRANULATION | 65 | Down | <0.0001 | <0.0001 |
| ORM1 STXBP2 | GO_EXOCYTOSIS | 1042 | Down | <0.0001 | 0.0003 |
| STXBP2 | GO_VESICLE_DOCKING | 68 | Down | 0.002 | 0.0542 |
| SCARB2 | GO_RECEPTOR_MEDIATED_ENDOCYTOSIS | 234 | Down | 0.001 | 0.0351 |
| ORM1 STXBP2 | GO_PLATELET_DEGRANULATION | 120 | Down | <0.0001 | 0.0003 |
| ISG15 | GO_INTEGRIN_MEDIATED_SIGNALING_PATHWAY | 121 | Down | <0.0001 | 0.0013 |
| OAS2 RPS4Y1 | GO_RNA_CATABOLIC_PROCESS | 768 | Up | <0.0001 | <0.0001 |
| SCARB2 | GO_ENDOCYTOSIS | 522 | Down | 0.0021 | 0.0551 |
| ORM1 | GO_POSITIVE_REGULATION_OF_SECRETION | 387 | Down | 0.0043 | 0.0849 |
| ISG15 | GO_DEFENSE_RESPONSE_TO_BACTERIUM | 177 | Down | 0.004 | 0.0809 |
| ORM1 | GO_ACUTE_PHASE_RESPONSE | 34 | Down | 0.0001 | 0.0069 |
| ORM1 BTN3A3 OAS2 STXBP2 ISG15 IFIT3 | GO_IMMUNE_EFFECTOR_PROCESS | 1454 | Down | 0.0052 | 0.0946 |
| STXBP2 | GO_PHAGOCYTIC_VESICLE | 166 | Down | <0.0001 | <0.0001 |
| ORM1 STXBP2 | GO_SECRETORY_GRANULE | 918 | Down | <0.0001 | 0.0006 |
| STXBP2 SCARB2 | GO_VESICLE_MEMBRANE | 828 | Down | <0.0001 | 0.0048 |
| ORM1 | GO_PLATELET_ALPHA_GRANULE | 82 | Down | 0.0001 | 0.0069 |
| RPS4Y1 RPS4Y2 | GO_RIBOSOME | 536 | Up | <0.0001 | <0.0001 |
| ORM1 STXBP2 | GO_SECRETORY_VESICLE | 1039 | Down | <0.0001 | 0.0012 |
| ORM1 | GO_VESICLE_LUMEN | 397 | Down | 0.0019 | 0.0525 |
| RPS4Y1 RPS4Y2 | GO_CYTOSOLIC_SMALL_RIBOSOMAL_SUBUNIT | 174 | Up | <0.0001 | <0.0001 |
| TIMM10 | GO_ORGANELLE_INNER_MEMBRANE | 537 | Up | 0.0041 | 0.0824 |
| RPS4Y1 RPS4Y2 | GO_SMALL_RIBOSOMAL_SUBUNIT | 210 | Up | <0.0001 | <0.0001 |
| ORM1 | GO_PLATELET_ALPHA_GRANULE_LUMEN | 59 | Down | 0.0004 | 0.0179 |
| ORM1 STXBP2 | GO_SPECIFIC_GRANULE | 214 | Down | <0.0001 | <0.0001 |
| SCARB2 | GO_VACUOLAR_LUMEN | 201 | Down | 0.0054 | 0.0963 |
| TIMM10 | GO_INNER_MITOCHONDRIAL_MEMBRANE_PROTEIN_COMPLEX | 143 | Up | 0.0013 | 0.0422 |
| STXBP2 | GO_SECRETORY_GRANULE_MEMBRANE | 361 | Down | <0.0001 | <0.0001 |
| RPS4Y1 | GO_POLYSOME | 179 | Up | <0.0001 | <0.0001 |
| RPS4Y1 RPS4Y2 | GO_RIBOSOMAL_SUBUNIT | 490 | Up | <0.0001 | <0.0001 |
| NARF KDM5D | GO_NUCLEOLUS | 1177 | Up | 0.0049 | 0.0914 |
| RPS4Y1 RPS4Y2 | GO_RIBONUCLEOPROTEIN_COMPLEX | 1147 | Up | <0.0001 | <0.0001 |
| TIMM10 | GO_MITOCHONDRIAL_PROTEIN_COMPLEX | 298 | Up | <0.0001 | 0.0013 |
| RPS4Y1 RPS4Y2 | GO_CYTOSOLIC_RIBOSOME | 384 | Up | <0.0001 | <0.0001 |
| EIF1AY | GO_TRANSLATION_INITIATION_FACTOR_ACTIVITY | 71 | Up | 0.0007 | 0.0279 |
| RPS4Y1 RPS4Y2 | GO_STRUCTURAL_CONSTITUENT_OF_RIBOSOME | 462 | Up | <0.0001 | <0.0001 |
| EIF1AY | GO_TRANSLATION_FACTOR_ACTIVITY_RNA_BINDING | 128 | Up | <0.0001 | 0.0001 |
| SCARB2 | GO_CARGO_RECEPTOR_ACTIVITY | 51 | Down | 0.003 | 0.0682 |
| RPS4Y1 RPS4Y2 | GO_RRNA_BINDING | 119 | Up | <0.0001 | <0.0001 |
| EIF1AY | GO_TRANSLATION_REGULATOR_ACTIVITY | 200 | Up | <0.0001 | 0.0003 |
| EIF1AY | GO_TRANSLATION_REGULATOR_ACTIVITY_NUCLEIC_ACID_BINDING | 152 | Up | <0.0001 | 0.0003 |
| EFEMP2 RPS4Y1 RPS4Y2 | GO_STRUCTURAL_MOLECULE_ACTIVITY | 791 | Up | <0.0001 | <0.0001 |
| OAS2 EIF1AY RPS4Y1 RPS4Y2 | GO_RNA_BINDING | 2299 | Up | 0.0003 | 0.016 |
| RPS4Y1 | GO_NUCLEAR_TRANSCRIBED_MRNA_CATABOLIC_PROCESS | 513 | Up | <0.0001 | <0.0001 |
| STXBP2 | GO_REGULATION_OF_MYELOID_LEUKOCYTE_MEDIATED_IMMUNITY | 71 | Down | <0.0001 | 0.0008 |
| RPS4Y1 | GO_COTRANSLATIONAL_PROTEIN_TARGETING_TO_MEMBRANE | 382 | Up | <0.0001 | <0.0001 |
| TRIM8 RPS4Y1 | GO_VIRAL_GENE_EXPRESSION | 503 | Up | <0.0001 | <0.0001 |
| ORM1 | GO_INTERLEUKIN_1_BETA_PRODUCTION | 114 | Down | 0.0008 | 0.0293 |
| ORM1 | GO_INTERLEUKIN_6_PRODUCTION | 170 | Down | 0.0006 | 0.023 |
| EIF1AY RPS4Y1 RPS4Y2 | GO_PEPTIDE_BIOSYNTHETIC_PROCESS | 1052 | Up | <0.0001 | <0.0001 |
| ORM1 | GO_INTERLEUKIN_1_SECRETION | 76 | Down | 0.0014 | 0.0443 |
| STXBP2 | GO_EXOCYTIC_PROCESS | 77 | Down | 0.0024 | 0.0604 |
| TIMM10 | GO_TIM23_MITOCHONDRIAL_IMPORT_INNER_MEMBRANE_TRANSLOCASE_COMPLEX | 18 | Up | 0.0012 | 0.0388 |
| ORM1 | GO_SPECIFIC_GRANULE_LUMEN | 73 | Down | <0.0001 | <0.0001 |
| STXBP2 | GO_AZUROPHIL_GRANULE | 209 | Down | <0.0001 | 0.0015 |
| ORM1 STXBP2 | GO_TERTIARY_GRANULE | 224 | Down | <0.0001 | <0.0001 |
| STXBP2 SCARB2 | GO_WHOLE_MEMBRANE | 1768 | Down | 0.0052 | 0.0947 |
| ORM1 | GO_TERTIARY_GRANULE_LUMEN | 76 | Down | <0.0001 | 0.0044 |
| OAS2 RPS4Y1 | GO_CELLULAR_NITROGEN_COMPOUND_CATABOLIC_PROCESS | 943 | Up | <0.0001 | 0.0004 |
| ORM1 | GO_REGULATION_OF_INTERLEUKIN_1_SECRETION | 64 | Down | 0.001 | 0.0341 |
| **UFP 21-27** |  |  |  |  |  |
| **GeneSymbols** | **Gene Pathway** | **NGenes** | **Direction** | **PValue** | **FDR** |
| SERPING1 | GO_REGULATION_OF_WOUND_HEALING | 134 | Down | 0.0022 | 0.0901 |
| SERPING1 | GO_RESPONSE_TO_WOUNDING | 627 | Down | 0.0014 | 0.0703 |
| EIF1AY RPS4Y1 | GO_TRANSLATIONAL_INITIATION | 497 | Up | <0.0001 | <0.0001 |
| RPS4Y1 TIMM10 | GO_PROTEIN_TARGETING | 739 | Up | <0.0001 | <0.0001 |
| STXBP2 | GO_REGULATION_OF_EXOCYTOSIS | 190 | Down | <0.0001 | 0.0025 |
| OAS2 SERPING1 | GO_REGULATION_OF_BODY_FLUID_LEVELS | 452 | Down | 0.0023 | 0.0932 |
| RPS4Y1 | GO_ESTABLISHMENT_OF_PROTEIN_LOCALIZATION_TO_ENDOPLASMIC_RETICULUM | 396 | Up | <0.0001 | <0.0001 |
| STXBP2 | GO_CELL_ACTIVATION | 1616 | Down | 0.0017 | 0.0775 |
| RPS4Y1 TIMM10 | GO_ESTABLISHMENT_OF_PROTEIN_LOCALIZATION_TO_ORGANELLE | 904 | Up | <0.0001 | 0.0087 |
| STXBP2 BTN3A3 SERPING1 | GO_LEUKOCYTE_MEDIATED_IMMUNITY | 988 | Down | 0.0003 | 0.0243 |
| STXBP2 | GO_REGULATION_OF_VESICLE_MEDIATED_TRANSPORT | 511 | Down | 0.0002 | 0.02 |
| SERPING1 | GO_WOUND_HEALING | 520 | Down | 0.0006 | 0.0386 |
| RPS4Y1 | GO_PROTEIN_LOCALIZATION_TO_ENDOPLASMIC_RETICULUM | 423 | Up | <0.0001 | <0.0001 |
| SERPING1 | GO_NEGATIVE_REGULATION_OF_COAGULATION | 44 | Down | 0.0025 | 0.0997 |
| EIF1AY RPS4Y1 RPS4Y2 | GO_PEPTIDE_METABOLIC_PROCESS | 1190 | Up | 0.0001 | 0.0117 |
| STXBP2 OAS2 SERPING1 ISG15 | GO_SECRETION | 1645 | Down | 0.0024 | 0.0955 |
| STXBP2 | GO_CELL_ACTIVATION_INVOLVED_IN_IMMUNE_RESPONSE | 928 | Down | 0.0002 | 0.0175 |
| RPS4Y1 | GO_PROTEIN_TARGETING_TO_MEMBRANE | 473 | Up | <0.0001 | <0.0001 |
| RPS4Y1 TIMM10 | GO_PROTEIN_LOCALIZATION_TO_MEMBRANE | 896 | Up | 0.0004 | 0.0319 |
| STXBP2 | GO_MYELOID_LEUKOCYTE_ACTIVATION | 867 | Down | <0.0001 | 0.0094 |
| EIF1AY RPS4Y1 RPS4Y2 | GO_CELLULAR_AMIDE_METABOLIC_PROCESS | 1444 | Up | 0.0021 | 0.0881 |
| EIF1AY RPS4Y1 RPS4Y2 | GO_AMIDE_BIOSYNTHETIC_PROCESS | 1194 | Up | <0.0001 | 0.0096 |
| RPS4Y1 TIMM10 | GO_ESTABLISHMENT_OF_PROTEIN_LOCALIZATION_TO_MEMBRANE | 626 | Up | <0.0001 | <0.0001 |
| STXBP2 | GO_REGULATION_OF_REGULATED_SECRETORY_PATHWAY | 137 | Down | <0.0001 | 0.0043 |
| STXBP2 | GO_MYELOID_LEUKOCYTE_MEDIATED_IMMUNITY | 760 | Down | <0.0001 | 0.0025 |
| OAS2 RPS4Y1 | GO_ORGANIC_CYCLIC_COMPOUND_CATABOLIC_PROCESS | 968 | Up | <0.0001 | 0.0089 |
| RPS4Y1 | GO_NUCLEAR_TRANSCRIBED_MRNA_CATABOLIC_PROCESS_NONSENSE_MEDIATED_DECAY | 411 | Up | <0.0001 | <0.0001 |
| STXBP2 | GO_REGULATION_OF_LEUKOCYTE_DEGRANULATION | 65 | Down | <0.0001 | 0.0002 |
| STXBP2 SERPING1 | GO_EXOCYTOSIS | 1042 | Down | <0.0001 | 0.0034 |
| STXBP2 SERPING1 | GO_PLATELET_DEGRANULATION | 120 | Down | <0.0001 | 0.0048 |
| ISG15 | GO_INTEGRIN_MEDIATED_SIGNALING_PATHWAY | 121 | Down | <0.0001 | 0.0116 |
| OAS2 RPS4Y1 | GO_RNA_CATABOLIC_PROCESS | 768 | Up | <0.0001 | 0.0003 |
| STXBP2 | GO_PHAGOCYTIC_VESICLE | 166 | Down | <0.0001 | 0.0066 |
| STXBP2 SERPING1 | GO_SECRETORY_GRANULE | 918 | Down | 0.0002 | 0.0185 |
| STXBP2 | GO_VESICLE_MEMBRANE | 828 | Down | 0.0009 | 0.0541 |
| SERPING1 | GO_PLATELET_ALPHA_GRANULE | 82 | Down | 0.0025 | 0.0989 |
| RPS4Y1 RPS4Y2 | GO_RIBOSOME | 536 | Up | <0.0001 | <0.0001 |
| STXBP2 SERPING1 | GO_SECRETORY_VESICLE | 1039 | Down | 0.0003 | 0.0233 |
| SERPING1 | GO_VESICLE_LUMEN | 397 | Down | 0.0005 | 0.0331 |
| RPS4Y1 RPS4Y2 | GO_CYTOSOLIC_SMALL_RIBOSOMAL_SUBUNIT | 174 | Up | <0.0001 | <0.0001 |
| RPS4Y1 RPS4Y2 | GO_SMALL_RIBOSOMAL_SUBUNIT | 210 | Up | <0.0001 | <0.0001 |
| STXBP2 | GO_SPECIFIC_GRANULE | 214 | Down | <0.0001 | <0.0001 |
| STXBP2 | GO_SECRETORY_GRANULE_MEMBRANE | 361 | Down | <0.0001 | 0.0003 |
| RPS4Y1 | GO_POLYSOME | 179 | Up | <0.0001 | <0.0001 |
| RPS4Y1 RPS4Y2 | GO_RIBOSOMAL_SUBUNIT | 490 | Up | <0.0001 | <0.0001 |
| RPS4Y1 RPS4Y2 | GO_RIBONUCLEOPROTEIN_COMPLEX | 1147 | Up | <0.0001 | 0.0074 |
| RPS4Y1 RPS4Y2 | GO_CYTOSOLIC_RIBOSOME | 384 | Up | <0.0001 | <0.0001 |
| RPS4Y1 RPS4Y2 | GO_STRUCTURAL_CONSTITUENT_OF_RIBOSOME | 462 | Up | <0.0001 | <0.0001 |
| RPS4Y1 RPS4Y2 | GO_RRNA_BINDING | 119 | Up | <0.0001 | <0.0001 |
| RPS4Y1 RPS4Y2 | GO_STRUCTURAL_MOLECULE_ACTIVITY | 791 | Up | <0.0001 | <0.0001 |
| RPS4Y1 | GO_NUCLEAR_TRANSCRIBED_MRNA_CATABOLIC_PROCESS | 513 | Up | <0.0001 | <0.0001 |
| STXBP2 | GO_REGULATION_OF_MYELOID_LEUKOCYTE_MEDIATED_IMMUNITY | 71 | Down | <0.0001 | 0.0082 |
| RPS4Y1 | GO_COTRANSLATIONAL_PROTEIN_TARGETING_TO_MEMBRANE | 382 | Up | <0.0001 | <0.0001 |
| TRIM8 RPS4Y1 | GO_VIRAL_GENE_EXPRESSION | 503 | Up | <0.0001 | <0.0001 |
| EIF1AY RPS4Y1 RPS4Y2 | GO_PEPTIDE_BIOSYNTHETIC_PROCESS | 1052 | Up | <0.0001 | 0.0012 |
| SERPING1 | GO_COAGULATION | 334 | Down | 0.0004 | 0.0303 |
| STXBP2 | GO_AZUROPHIL_GRANULE | 209 | Down | <0.0001 | 0.0036 |
| STXBP2 | GO_TERTIARY_GRANULE | 224 | Down | <0.0001 | 0.0018 |
| OAS2 RPS4Y1 | GO_CELLULAR_NITROGEN_COMPOUND_CATABOLIC_PROCESS | 943 | Up | <0.0001 | 0.0077 |

**Table S15. Differentially expressed genes present in specific gene pathways associated with increased concentrations of accumulation mode particles at multiple lag periods**

| **AMP 0-6** |  |  |  |  |  |
| --- | --- | --- | --- | --- | --- |
| **GeneSymbols** | **Gene Pathway** | **NGenes** | **Direction** | **PValue** | **FDR** |
| TRIM8 ISG15 RSAD2 | GO_NEGATIVE_REGULATION_OF_VIRAL_PROCESS | 124 | Up | 0.0015 | 0.0625 |
| EIF1AY RPS4Y1 | GO_TRANSLATIONAL_INITIATION | 497 | Up | <0.0001 | <0.0001 |
| IFI27 ISG15 RSAD2 | GO_RESPONSE_TO_TYPE_I_INTERFERON | 125 | Up | 0.0022 | 0.0803 |
| RPS4Y1 | GO_PROTEIN_TARGETING | 739 | Up | 0.0003 | 0.0232 |
| MAPK3 | GO_LUNG_MORPHOGENESIS | 47 | Down | 0.0002 | 0.0191 |
| RPS4Y1 | GO_ESTABLISHMENT_OF_PROTEIN_LOCALIZATION_TO_ENDOPLASMIC_RETICULUM | 396 | Up | <0.0001 | <0.0001 |
| IFI27 ISG15 RSAD2 | GO_DEFENSE_RESPONSE_TO_VIRUS | 296 | Up | 0.0015 | 0.0625 |
| RPS4Y1 | GO_ESTABLISHMENT_OF_PROTEIN_LOCALIZATION_TO_ORGANELLE | 904 | Up | 0.0011 | 0.0519 |
| FABP5 STXBP2 RSAD2 | GO_LEUKOCYTE_MEDIATED_IMMUNITY | 988 | Down | 0.0002 | 0.014 |
| RPS4Y1 | GO_PROTEIN_LOCALIZATION_TO_ENDOPLASMIC_RETICULUM | 423 | Up | <0.0001 | 0.0001 |
| RARA EIF1AY RPS4Y1 RPS4Y2 CPQ MAPK3 | GO_PEPTIDE_METABOLIC_PROCESS | 1190 | Up | 0.0006 | 0.0333 |
| FABP5 STXBP2 MAPK3 ISG15 RSAD2 | GO_SECRETION | 1645 | Down | 0.0007 | 0.0388 |
| FABP5 STXBP2 RARA | GO_CELL_ACTIVATION_INVOLVED_IN_IMMUNE_RESPONSE | 928 | Down | <0.0001 | 0.0058 |
| RPS4Y1 | GO_PROTEIN_TARGETING_TO_MEMBRANE | 473 | Up | <0.0001 | 0.001 |
| FABP5 STXBP2 | GO_MYELOID_LEUKOCYTE_ACTIVATION | 867 | Down | <0.0001 | 0.0008 |
| RARA EIF1AY RPS4Y1 RPS4Y2 MAPK3 | GO_AMIDE_BIOSYNTHETIC_PROCESS | 1194 | Up | 0.0003 | 0.0237 |
| MAPK3 | GO_GRANULOCYTE_MIGRATION | 113 | Down | <0.0001 | 0.0075 |
| RARA MAPK3 | GO_FACE_DEVELOPMENT | 43 | Down | 0.0029 | 0.0967 |
| RPS4Y1 | GO_ESTABLISHMENT_OF_PROTEIN_LOCALIZATION_TO_MEMBRANE | 626 | Up | <0.0001 | 0.0083 |
| IFI27 ISG15 RSAD2 | GO_VIRAL_GENOME_REPLICATION | 184 | Up | 0.0008 | 0.0431 |
| STXBP2 | GO_REGULATION_OF_REGULATED_SECRETORY_PATHWAY | 137 | Down | 0.0019 | 0.0714 |
| FABP5 STXBP2 | GO_MYELOID_LEUKOCYTE_MEDIATED_IMMUNITY | 760 | Down | <0.0001 | 0.0001 |
| UPB1 RPS4Y1 | GO_ORGANIC_CYCLIC_COMPOUND_CATABOLIC_PROCESS | 968 | Up | 0.0005 | 0.03 |
| MAPK3 | GO_TRACHEA_MORPHOGENESIS | 15 | Down | 0.0002 | 0.0172 |
| RPS4Y1 | GO_NUCLEAR_TRANSCRIBED_MRNA_CATABOLIC_PROCESS_NONSENSE_MEDIATED_DECAY | 411 | Up | <0.0001 | <0.0001 |
| STXBP2 | GO_REGULATION_OF_LEUKOCYTE_DEGRANULATION | 65 | Down | <0.0001 | 0.0017 |
| FABP5 STXBP2 | GO_EXOCYTOSIS | 1042 | Down | <0.0001 | 0.0035 |
| IFI27 ISG15 RSAD2 | GO_REGULATION_OF_VIRAL_GENOME_REPLICATION | 139 | Up | <0.0001 | 0.0009 |
| ISG15 | GO_INTEGRIN_MEDIATED_SIGNALING_PATHWAY | 121 | Down | 0.0012 | 0.0547 |
| ISG15 RSAD2 | GO_NEGATIVE_REGULATION_OF_VIRAL_GENOME_REPLICATION | 81 | Up | <0.0001 | 0.0058 |
| RPS4Y1 | GO_RNA_CATABOLIC_PROCESS | 768 | Up | <0.0001 | 0.0043 |
| SLC9A1 | GO_AMINOGLYCAN_CATABOLIC_PROCESS | 48 | Down | 0.0006 | 0.0368 |
| RARA MAPK3 | GO_PHAGOCYTOSIS | 322 | Down | <0.0001 | 0.0078 |
| RARA | GO_NEGATIVE_REGULATION_OF_TUMOR_NECROSIS_FACTOR_SUPERFAMILY_CYTOKINE_PRODUCTION | 75 | Down | 0.0027 | 0.0911 |
| MAPK3 | GO_THYROID_GLAND_DEVELOPMENT | 15 | Down | 0.0023 | 0.0805 |
| MAPK3 | GO_NEURAL_CREST_CELL_DIFFERENTIATION | 62 | Down | 0.0017 | 0.0689 |
| MAPK3 | GO_ACTIVATION_OF_MAPKK_ACTIVITY | 55 | Down | 0.0009 | 0.0437 |
| STXBP2 | GO_PHAGOCYTIC_VESICLE | 166 | Down | <0.0001 | 0.0028 |
| FABP5 STXBP2 | GO_SECRETORY_GRANULE | 918 | Down | <0.0001 | 0.0032 |
| RPS4Y1 RPS4Y2 | GO_RIBOSOME | 536 | Up | <0.0001 | <0.0001 |
| FABP5 STXBP2 | GO_SECRETORY_VESICLE | 1039 | Down | <0.0001 | 0.0069 |
| FABP5 | GO_VESICLE_LUMEN | 397 | Down | 0.0009 | 0.0443 |
| RPS4Y1 RPS4Y2 | GO_CYTOSOLIC_SMALL_RIBOSOMAL_SUBUNIT | 174 | Up | <0.0001 | 0.0017 |
| RPS4Y1 RPS4Y2 | GO_SMALL_RIBOSOMAL_SUBUNIT | 210 | Up | <0.0001 | 0.0008 |
| STXBP2 | GO_SPECIFIC_GRANULE | 214 | Down | <0.0001 | <0.0001 |
| FABP5 STXBP2 | GO_SECRETORY_GRANULE_MEMBRANE | 361 | Down | <0.0001 | <0.0001 |
| RPS4Y1 | GO_POLYSOME | 179 | Up | <0.0001 | 0.0065 |
| RPS4Y1 RPS4Y2 | GO_RIBOSOMAL_SUBUNIT | 490 | Up | <0.0001 | <0.0001 |
| MAPK3 | GO_PSEUDOPODIUM | 16 | Down | <0.0001 | 0.0059 |
| RPS4Y1 RPS4Y2 | GO_RIBONUCLEOPROTEIN_COMPLEX | 1147 | Up | <0.0001 | 0.0067 |
| RPS4Y1 RPS4Y2 | GO_CYTOSOLIC_RIBOSOME | 384 | Up | <0.0001 | <0.0001 |
| ALPL | GO_ANCHORED_COMPONENT_OF_MEMBRANE | 90 | Down | 0.001 | 0.0471 |
| EIF1AY | GO_TRANSLATION_INITIATION_FACTOR_ACTIVITY | 71 | Up | 0.002 | 0.0753 |
| MAPK3 | GO_MAP_KINASE_ACTIVITY | 16 | Down | <0.0001 | 0.0076 |
| RPS4Y1 RPS4Y2 | GO_STRUCTURAL_CONSTITUENT_OF_RIBOSOME | 462 | Up | <0.0001 | <0.0001 |
| EIF1AY | GO_TRANSLATION_FACTOR_ACTIVITY_RNA_BINDING | 128 | Up | 0.0004 | 0.0262 |
| RSAD2 | GO_METAL_CLUSTER_BINDING | 67 | Up | <0.0001 | 0.0082 |
| RPS4Y1 RPS4Y2 | GO_RRNA_BINDING | 119 | Up | <0.0001 | 0.0017 |
| RARA EIF1AY | GO_TRANSLATION_REGULATOR_ACTIVITY | 200 | Up | 0.0017 | 0.0665 |
| RARA EIF1AY | GO_TRANSLATION_REGULATOR_ACTIVITY_NUCLEIC_ACID_BINDING | 152 | Up | 0.0012 | 0.0553 |
| RPS4Y1 RPS4Y2 | GO_STRUCTURAL_MOLECULE_ACTIVITY | 791 | Up | 0.0001 | 0.0126 |
| RPS4Y1 | GO_NUCLEAR_TRANSCRIBED_MRNA_CATABOLIC_PROCESS | 513 | Up | <0.0001 | <0.0001 |
| MAPK3 | GO_FC_RECEPTOR_MEDIATED_STIMULATORY_SIGNALING_PATHWAY | 127 | Down | 0.0009 | 0.0442 |
| STXBP2 | GO_REGULATION_OF_MYELOID_LEUKOCYTE_MEDIATED_IMMUNITY | 71 | Down | 0.0001 | 0.0116 |
| RPS4Y1 | GO_COTRANSLATIONAL_PROTEIN_TARGETING_TO_MEMBRANE | 382 | Up | <0.0001 | <0.0001 |
| TRIM8 RPS4Y1 | GO_VIRAL_GENE_EXPRESSION | 503 | Up | <0.0001 | 0.0002 |
| RARA EIF1AY RPS4Y1 RPS4Y2 MAPK3 | GO_PEPTIDE_BIOSYNTHETIC_PROCESS | 1052 | Up | <0.0001 | 0.0065 |
| MAPK3 | GO_ANIMAL_ORGAN_FORMATION | 35 | Down | 0.0012 | 0.0543 |
| MAPK3 | GO_BERGMANN_GLIAL_CELL_DIFFERENTIATION | 10 | Down | 0.0007 | 0.0388 |
| MAPK3 | GO_TRACHEA_FORMATION | 13 | Down | 0.0001 | 0.0126 |
| MAPK3 | GO_CARDIAC_NEURAL_CREST_CELL_DIFFERENTIATION_INVOLVED_IN_HEART_DEVELOPMENT | 11 | Down | 0.0004 | 0.025 |
| MAPK3 | GO_CAVEOLIN_MEDIATED_ENDOCYTOSIS | 14 | Down | 0.0014 | 0.0606 |
| IFI27 TRIM8 ISG15 RSAD2 | GO_REGULATION_OF_VIRAL_LIFE_CYCLE | 192 | Up | 0.0003 | 0.022 |
| MAPK3 | GO_REGULATION_OF_METALLOPEPTIDASE_ACTIVITY | 28 | Down | <0.0001 | 0.002 |
| MAPK3 | GO_POSITIVE_REGULATION_OF_METALLOPEPTIDASE_ACTIVITY | 19 | Down | 0.0002 | 0.0161 |
| FABP5 | GO_AZUROPHIL_GRANULE_LUMEN | 121 | Down | 0.0011 | 0.0498 |
| FABP5 STXBP2 | GO_AZUROPHIL_GRANULE | 209 | Down | <0.0001 | 0.0017 |
| STXBP2 | GO_TERTIARY_GRANULE | 224 | Down | <0.0001 | <0.0001 |
| RARA | GO_MRNA_5_UTR_BINDING | 58 | Up | 0.0001 | 0.0116 |
| UPB1 RPS4Y1 | GO_CELLULAR_NITROGEN_COMPOUND_CATABOLIC_PROCESS | 943 | Up | 0.0003 | 0.0201 |
| TRIM8 ISG15 RSAD2 | GO_NEGATIVE_REGULATION_OF_VIRAL_LIFE_CYCLE | 107 | Up | 0.0005 | 0.03 |
| **AMP 21-27** |  |  |  |  |  |
| **GeneSymbols** | **Gene Pathway** | **NGenes** | **Direction** | **PValue** | **FDR** |
| RPS12 RPS4Y1 | GO_ESTABLISHMENT_OF_PROTEIN_LOCALIZATION_TO_ENDOPLASMIC_RETICULUM | 396 | Up | 0.0008 | 0.0914 |
| FTH1 | GO_NEGATIVE_REGULATION_OF_FIBROBLAST_PROLIFERATION | 45 | Up | 0.0005 | 0.0699 |
| FTH1 | GO_SEQUESTERING_OF_METAL_ION | 26 | Up | <0.0001 | 0.0155 |
| HNRNPM | GO_RNA_SPLICING_VIA_TRANSESTERIFICATION_REACTIONS | 448 | Down | 0.0006 | 0.0751 |
| RPS12 RPS4Y1 | GO_NUCLEAR_TRANSCRIBED_MRNA_CATABOLIC_PROCESS_NONSENSE_MEDIATED_DECAY | 411 | Up | 0.0003 | 0.056 |
| USPL1 | GO_CAJAL_BODY | 67 | Down | <0.0001 | 0.0193 |
| SERPING1 | GO_VESICLE_LUMEN | 397 | Down | <0.0001 | 0.0193 |
| RPS12 RPS4Y1 RPS4Y2 | GO_CYTOSOLIC_SMALL_RIBOSOMAL_SUBUNIT | 174 | Up | <0.0001 | 0.0193 |
| STXBP2 | GO_SPECIFIC_GRANULE | 214 | Down | <0.0001 | 0.0222 |
| HNRNPM | GO_CATALYTIC_STEP_2_SPLICEOSOME | 141 | Down | <0.0001 | 0.0057 |
| HNRNPM | GO_SPLICEOSOMAL_COMPLEX | 267 | Down | 0.0001 | 0.0317 |
| TIMM10 | GO_MITOCHONDRIAL_PROTEIN_COMPLEX | 298 | Down | 0.0007 | 0.0836 |
| RPS12 RPS4Y1 RPS4Y2 | GO_CYTOSOLIC_RIBOSOME | 384 | Up | <0.0001 | 0.0218 |
| FTH1 | GO_OXIDOREDUCTASE_ACTIVITY_OXIDIZING_METAL_IONS | 27 | Up | <0.0001 | 0.0016 |
| FTH1 | GO_FERROUS_IRON_BINDING | 34 | Up | <0.0001 | <0.0001 |
| RPS12 RPS4Y1 | GO_COTRANSLATIONAL_PROTEIN_TARGETING_TO_MEMBRANE | 382 | Up | 0.0002 | 0.0462 |
| FTH1 | GO_SEQUESTERING_OF_IRON_ION | 20 | Up | <0.0001 | 0.0016 |
| FTH1 | GO_SECONDARY_LYSOSOME | 37 | Up | <0.0001 | 0.0193 |
| HNRNPM | GO_PARASPECKLES | 10 | Down | <0.0001 | 0.0219 |
| STXBP2 | GO_AZUROPHIL_GRANULE | 209 | Down | 0.0006 | 0.0751 |
| FTH1 | GO_AUTOLYSOSOME | 30 | Up | <0.0001 | 0.0018 |
| HNRNPM | GO_POST_MRNA_RELEASE_SPLICEOSOMAL_COMPLEX | 20 | Down | 0.0001 | 0.03 |
| FTH1 | GO_FERRIC_IRON_BINDING | 17 | Up | <0.0001 | <0.0001 |
| FTH1 | GO_OXIDOREDUCTASE_ACTIVITY_OXIDIZING_METAL_IONS_OXYGEN_AS_ACCEPTOR | 17 | Up | <0.0001 | <0.0001 |
